# Annulation cascade of arylnitriles with alkynes to stable delocalized PAH carbocations *via* intramolecular rhodium migration†
†Electronic supplementary information (ESI) available: Detailed information on experimental procedures, characterization data, computational calculations, crystallographic and spectroscopic data and X-ray crystal structures. CCDC 1813496 (**3ea**) and 1813497 (**3ia**). For ESI and crystallographic data in CIF or other electronic format see DOI: 10.1039/c8sc01963k


**DOI:** 10.1039/c8sc01963k

**Published:** 2018-05-29

**Authors:** Jiangliang Yin, Fulin Zhou, Lei Zhu, Mufan Yang, Yu Lan, Jingsong You

**Affiliations:** a Key Laboratory of Green Chemistry and Technology of Ministry of Education , College of Chemistry , Sichuan University , 29 Wangjiang Road , Chengdu 610064 , PR China . Email: jsyou@scu.edu.cn; b School of Chemistry and Chemical Engineering , Chongqing University , Chongqing 400030 , PR China

## Abstract

Herein, we propose the conception of heteroatom-promoted delocalization of the positive charge of an oxonium ion and thus develop a highly efficient rhodium(iii)-catalyzed hydration and three fold C–H activation/annulation cascade of arylnitriles with alkynes, affording a structurally diverse family of delocalized polycyclic aromatic hydrocarbon (PAH) carbocations. DFT calculations demonstrate that the positive charge mostly locates around the C1 atom and is partly delocalized by ambient N, O1 and C5 atoms. A mechanistic study indicates that the hydration of the arylnitrile and three fold insertion of the alkyne is a successive process rather than a step by step process, wherein a unique intramolecular rhodium migration is probably involved. These novel carbeniums show tunable fluorescence emission, low cytotoxicity and the ability to specifically target lysosomes.

## Abstract 2

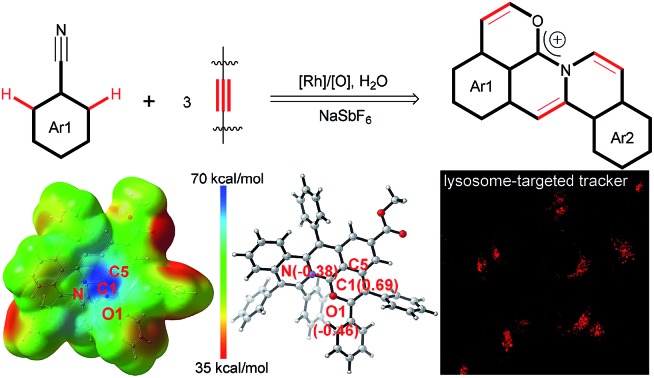
An annulation cascade of arylnitriles with alkynes is disclosed to provide stable delocalized carbocations with the ability to specifically target lysosomes.

## Introduction

**Scheme 1:**
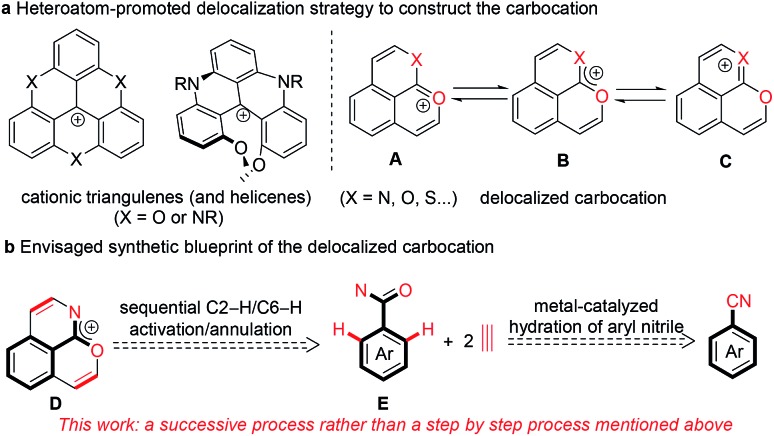
Synthetic blueprint of the positive charge-delocalized carbocation.

Stable polycyclic aromatic hydrocarbon (PAH) cations are of great fundamental interest and also have a wide range of potential applications in the area of organic synthetic chemistry, materials science, biology and photochemistry.^1^–^10^ Among these cationic skeletons, heteroatom cations such as pyridinium and pyrylium have been developed extensively.^6^–^10^ In contrast, due to the uncontrollable stability of carbocations, other examples are mostly confined to cationic triangulenes and helicenes (Scheme 1a), in which the positive charge is stabilized by the donor groups on the aromatic rings.^1^–^5^ Doubtlessly, structural diversity of carbocations is necessary for in-depth research both on the fundamental and application aspects.

In recent years, transition metal-catalyzed C–H transformations of simple arenes have attracted significant attention for accessing functional molecules and exploring novel reaction mechanisms.^11^–^13^ In particular, the rhodium-catalyzed C–H annulation of various arenes with alkynes has allowed great progress for the highly efficient construction of diverse heterocyclic compounds.^14^–^28^ Recently, our group reported a pyrylium cation synthesis *via* a rhodium-catalyzed sequential C–H activation/annulation process of naphthalene-type aldehydes with alkynes, in which the conjugated phenalenyl counterpart is conducive to stabilizing the pyrylium cation.^25^ The replacement of the carbon atom with other isostructural atoms at desired positions is currently considered as a versatile strategy to modify the charge carrier and chemical properties of PAHs. We envisaged that introducing a heteroatom like oxygen, sulfur or nitrogen at the *ortho* position of the adjacent ring of the pyrylium could enhance the molecular stability due to the interaction between a lone pair of electrons of the introduced heteroatom and an empty p orbital of the oxonium (Scheme 1a, **A**), and thus the resulting new delocalized carbocation could exhibit some intriguing properties. Herein we proposed that the positive charge delocalized carbocation **D** could be forged in one step directly by cascade [4 + 2] annulations of an arylamide with two equivalents of an alkyne through rhodium-catalyzed sequential C2–H/C6–H cleavages (Scheme 1b).^25^–^28^

To meet the demand for efficiency and economy, the development of more general, simple methods to access to complex functional skeletons using simple and easily available arenes as the substrate is highly desirable. Arylnitriles are one of the most basic units for diverse chemical transformations.^29^–^31^ Compared to conventional strong base or acid-promoted conversions, metal-catalyzed hydrolysis of organonitriles is an area of focus due to good selectivity in the preparation of compounds of synthetic and pharmacological significance and wide tolerance of sensitive functional groups.^30^ For the synthesis of organoamides from organonitriles, ruthenium and copper are usually used as the catalyst.^29^,^32^,^33^ Despite rare investigation, rhodium has also been demonstrated as a highly efficient catalyst for hydration of organonitriles.^34^ Thus, using structurally diverse and easily available arylnitriles as the starting material instead of arylamides would be a more advantageous route to the delocalized carbocation **D** (Scheme 1b). However, developing this strategy would be a conceptual and practical challenge. Firstly, the stability of the desired carbocation and their compatibility with the catalytic system is a big issue. Secondly, it is challenging to realize excellent selectivity and high efficiency in sequential hydration and C2–H/C6–H annulations of arylnitriles. Thirdly, it is difficult to implement two different heteroatom directed C–H activation processes in one catalytic system.

## Results and discussion

### Optimization of the reaction conditions

With these considerations in mind, we began our investigation using benzonitrile and diphenylacetylene as the standard substrates, [Cp*RhCl_2_]_2_ (5 mol%)/AgSbF_6_ (20 mol%) as the catalyst, AgOAc as the oxidant and CH_3_COOH as the additive in DCE (0.5 mL) at 120 °C under an N_2_ atmosphere (Table S1†). Gratifyingly, an orange solid product was present in 7% yield (Table S1,† entry 1). Subsequently, two equivalents of NaSbF_6_ were added into the reaction system as the counteranion source, obviously improving the yield of **3aa** to 39% (Table S1,† entry 2). There were almost no obvious by-products except the unreacted starting materials and the alkyne could be recovered in 52% yield. Considering the hydration of the arylnitrile, eight equivalents of water were next added, slightly improving the yield of **3aa** from 39% to 45% (Table S1,† entry 4). Other solvents such as THF, 1,4-dioxane and toluene led to poor yields of **3aa** or did not give any desired product (Table S1,† entries 6–8). After a careful screening of oxidants, Ag_2_O was found to be more efficient to the reaction, dramatically delivering the target molecule in 79% yield (Table S1,† entry 10). The yield could reach 88% by decreasing the amount of benzonitrile from 0.3 mmol to 0.2 mmol (Table S1,† entry 11). When the amount of NaSbF_6_ was decreased to 1.5 equivalents, the yield of **3aa** was almost kept at the same level (86%) (Table S1,† entry 12).

### Scope of arylnitriles for the synthesis of the delocalized carbocation

**Scheme 2:**
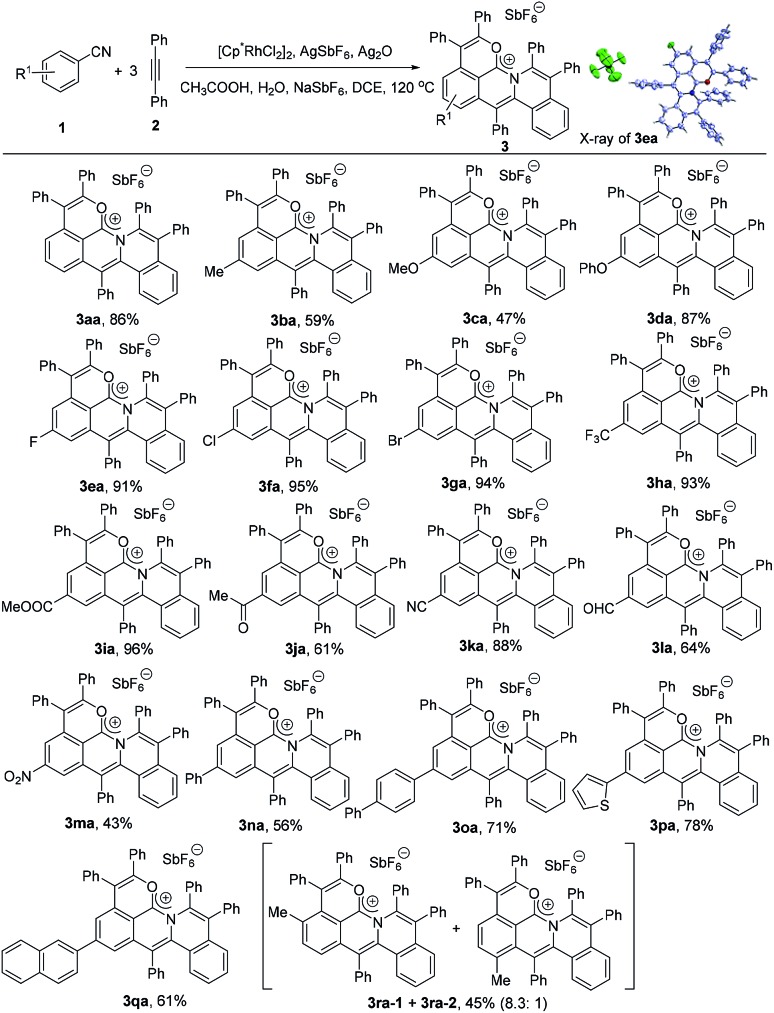
Scope of arylnitriles. Reaction conditions: **1** (0.2 mmol), **2a** (0.3 mmol), [Cp*RhCl_2_]_2_ (5 mol%), AgSbF_6_ (20 mol%), Ag_2_O (0.3 mmol), CH_3_COOH (6.0 equiv.), H_2_O (8.0 equiv.), NaSbF_6_ (1.5 equiv.) and DCE (0.5 mL) under N_2_ for 12 h.

To explore the substrate scope, the reactions of various arylnitriles with diphenylacetylene (**2a**) were first investigated under the optimized reaction conditions. As shown in Scheme 2, the annulations of arylnitriles allowed a wide scope of arylnitriles, producing a family of cations in moderate to excellent yields. First, arylnitriles with electron-donating groups such as methyl, methoxy and phenoxy at the *para* position gave 59%, 47% and 87% yields, respectively (Scheme 2, **3ba–3da**). Secondly, arylnitriles with electron-withdrawing groups at the *para*-position such as halide, trifluoromethyl, ester, aldehyde, ketone, nitro and even cyano could also smoothly undergo this annulation in 43% to 96% yields (Scheme 2, **3ea–3ma**). Thirdly, this protocol was also efficient for aryl and heteroaryl substituted arylnitriles (Scheme 2, **3na–3qa**). Finally, a *m*-methyl substituted arylnitrile gave 45% total yield with a high selectivity ratio of 8.3 : 1 according to the ^1^H NMR spectrum (Scheme 2, **3ra**).

### Scope of alkynes for the synthesis of the delocalized carbocations

**Scheme 3:**
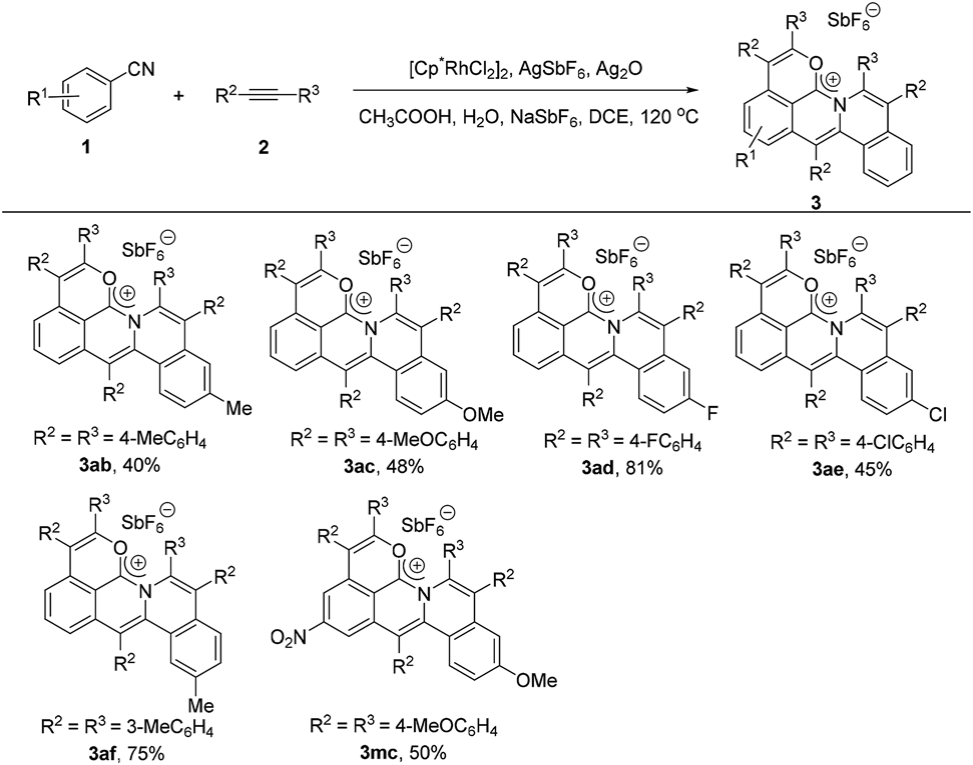
Scope of alkynes under standard reaction conditions.

Next, we investigated the scope of the alkyne derivatives. Both the electron-donating groups such as methyl and methoxy and the electron-withdrawing groups such as fluoro and chloro could be tolerated, giving the corresponding cations in moderate to good yields (Scheme 3, **3ab–3ae**). The *meta*-methyl substituted alkyne (**2f**) also proceeded well to afford **3af** in 75% yield with complete regioselectivity (Scheme 3, **3af**).

### X-ray single crystal diffraction analysis and DFT calculations

**Fig. 1:**
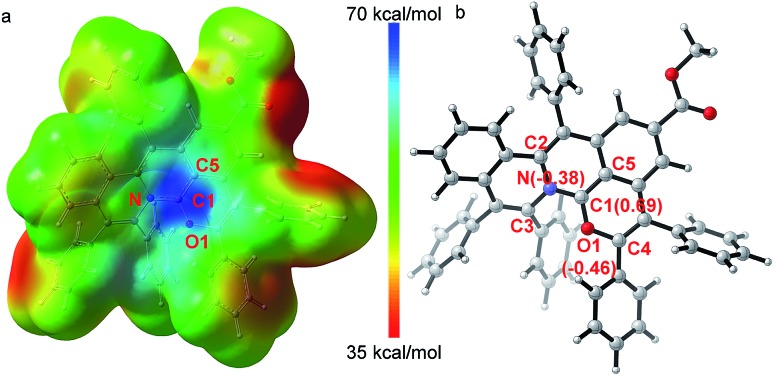
X-ray single crystal diffraction analysis and DFT calculations to demonstrate the salient cationic peculiarity of C1 atom. (a) Electrostatic potential maps. (b) Calculated NBO atomic charge distribution of cationic product **3ia**.

The X-ray single crystal diffraction of **3ea** and **3ia** further confirmeded the proposed product structures (Scheme 2, Tables S3 and S4†). As shown in Fig. 1, X-ray single crystal analysis of **3ia** shows that the cationic skeleton exhibits a twisted (helical) conformation. The length of the C1–N bond (1.348(6) Å) is clearly shorter than those of the C2–N bond (1.423(6) Å) and the C3–N bond (1.431(6) Å). The length of the C1–O1 bond (1.320(6) Å) is close to those of the previously reported C=O^+^ bonds (1.320–1.380 Å),^25^ obviously shorter than the C4–O1 single bond (1.397(5) Å). Furthermore, DFT calculations were employed to evaluate the intrinsic characteristics of cationic product **3ia**.^35^,^36^ As shown in Fig. 1a, the natural population analysis illustrates that the positive charge mostly locates around the C1 atom and is partly delocalized by ambient N, O1 and C5 atoms. Such a charge delocalization could contribute to the good stability of the desired carbocation. Fig. 1b shows that the variation of the NBO charges on the C1, O1 and N atoms are 0.69, –0.46 and –0.38, respectively, further demonstrating that the C1 atom exhibits salient cationic peculiarity.
1

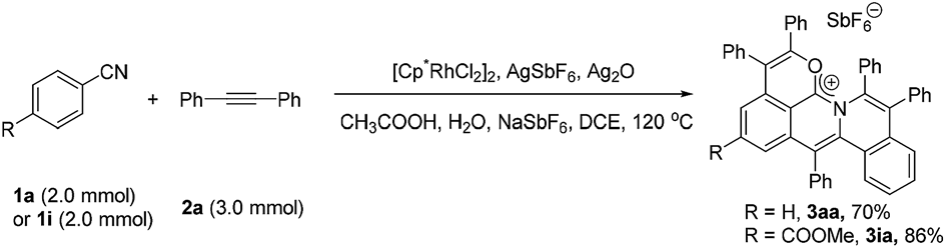




### Scale-up reactions

To further demonstrate the practicality and efficiency of this protocol, a scale-up pattern was investigated (eqn (1)). A 1.0 mmol scale reaction of benzonitrile (**1a**) or methyl 4-cyanobenzoate (**1i**) with diphenylacetylene (**2a**) was performed to give 70% and 86% yields, respectively (for details see the ESI†).

### Mechanistic investigation

**Scheme 4:**
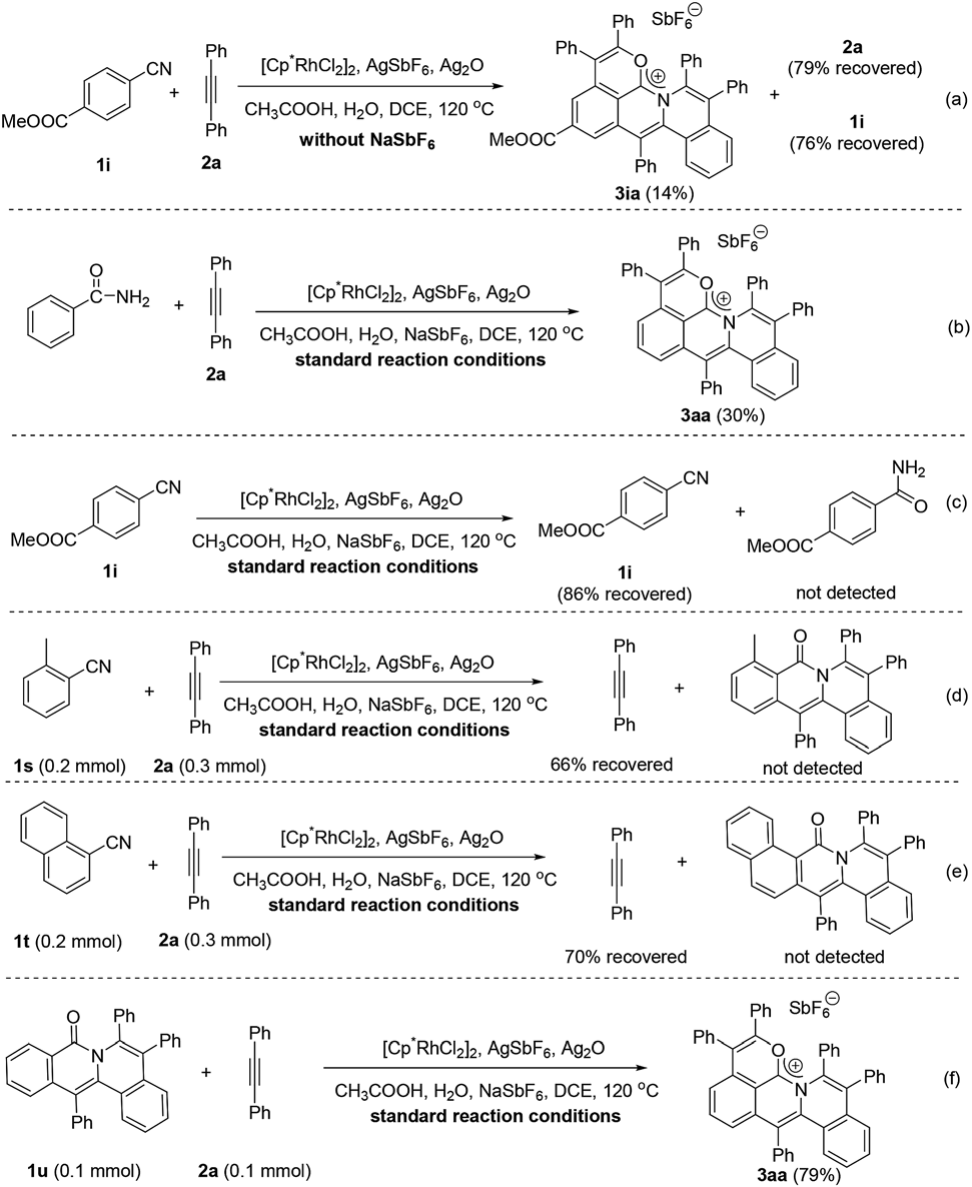
Control experiments for the reaction mechanism.

To gain more insight into the mechanism of this cascade protocol, control experiments were conducted (Scheme 4). Firstly, the reaction of methyl 4-cyanobenzoate (**1i**) with diphenylacetylene (**2a**) was performed without the addition of the extra anion source, NaSbF_6_, and **2a** and **1i** were recovered with 79% and 76% yields, respectively (Scheme 4a). The originally proposed intermediate amide, the 1 : 1 annulation product and the 1 : 2 annulation product were all not detected except a 14% yield of the 1 : 3 annulation product **3ia** (Scheme 4a). Benzamide could also be used as the starting material, but the yield of the three-fold annulation product **3aa** is low (only 30% yield) (Scheme 4b). In the absence of the alkyne **2a**, the reaction of **1i** did not lead to the formation of the benzamide derivative under the standard reaction conditions (Scheme 4c). The reactions of *ortho*-substituted aryl nitriles **1s** and **1t** with **2a** were performed under the standard reaction conditions, giving alkyne recovery rates of 66% and 70%, respectively, and the 1 : 2 annulation products were both not detected (Scheme 4d and e). In addition, the doubly annulated compound **1u** could react with **2a** to produce **3aa** in 79% yield (Scheme 4f), but we did not detect the 1 : 1 and 1 : 2 annulation products in our standard reactions of arylnitriles (Fig. S4†). These results indicate that the hydration of the arylnitrile and the three-fold insertion of alkyne is a successive transformation rather than a step by step process. It is reasonable to assume that a unique intramolecular rhodium migration may impel the occurrence of this successive transformation.^37^,^38^

2

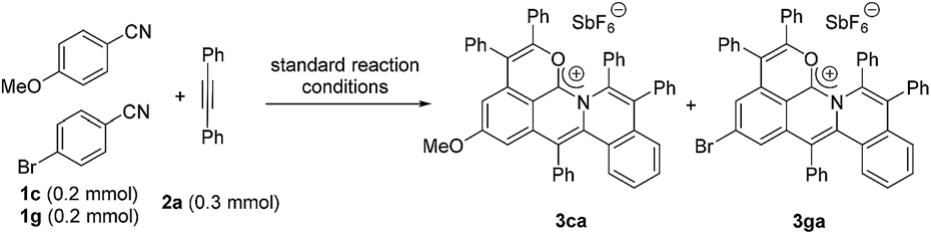




Next, an intermolecular competition experiment was conducted among equimolar amounts of electronically differentiated **1c** and **1g** with one equivalent of **2a** (eqn (2)). The desired products **3ca** and **3ga** were obtained with a ratio of 1 : 3.3, indicating that the more electron-deficient arylnitrile is favorable to the cascade reaction (Fig. S1†).^39^

**Scheme 5:**
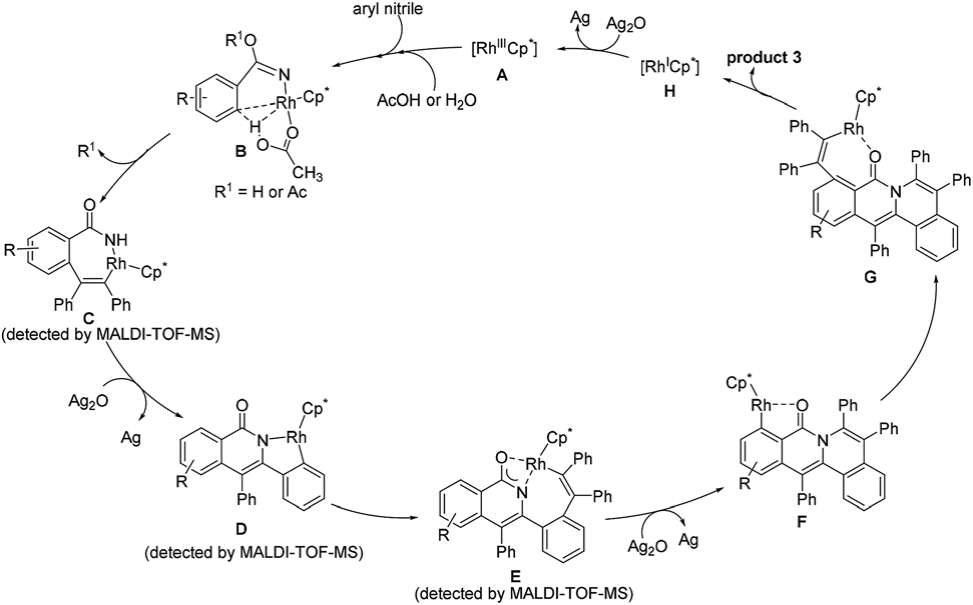
Proposed catalytic cycle.

In light of the experimental results and the known reports,^25^,^29^,^37^–^39^ we proposed the reaction mechanism (Scheme 5). Firstly, the CN group coordinates to the rhodium center to generate a highly electrophilic species, which is more susceptible to be attacked by a nucleophile such as acetic acid and water.^30^,^34^ Subsequently, C2–H activation takes place *via* an acetate-assisted C–H bond activation process to form the five-membered cyclic rhodium intermediate, followed by alkyne insertion into the C2–Rh bond to form a seven-membered intermediate **C**. The reductive elimination of **C** forms a N–Rh(i)-containing intermediate, which is rapidly oxidized to a N–Rh(iii)-containing species prior to the dissociation of the rhodium(i) species, followed by C–H activation to yield the intermediate **D**. Then, the second alkyne inserts into the Rh–C bond, and meanwhile the oxygen atom coordinates to the rhodium center to form the intermediate **E**. Notably, **E** does not undergo the reductive elimination process occurring typically in the literature to generate the double annulation product.^40^–^42^ We speculate that the generation of the three-fold annulation product could be related to the different reaction conditions associated with the oxidant and solvent. Under the current reaction conditions, after the reductive elimination of **E**, the resulting Rh(i) intermediate is rapidly oxidized to a Rh(iii) species and undergoes an oxygen atom-directed C6–H activation to form the intermediate **F**, which probably involves a unique intramolecular rhodium migration. Finally, the desired cation is obtained after the third alkyne insertion and reductive elimination. The active Rh(iii) species is regenerated by oxidizing Rh(i) with Ag_2_O.

### Photophysical properties of the representative products

The photophysical properties of the representative products were then measured. As shown in Table S2,† these carbocations feature tunable emission wavelengths and the substituents on the arylnitriles have a significant influence on the emission wavelengths. Generally, an increase in the electron-withdrawing ability from methoxy to nitro at the *para*-position of the arylnitrile enables bathochromic shifts from 549 nm to 622 nm in CH_2_Cl_2_. The emission of **3mc** in CH_2_Cl_2_ could shift to the near-infrared (NIR) region (674 nm) *via* regulating the substituent of the aryl nitrile and the alkyne.

### Biological application

**Fig. 2:**
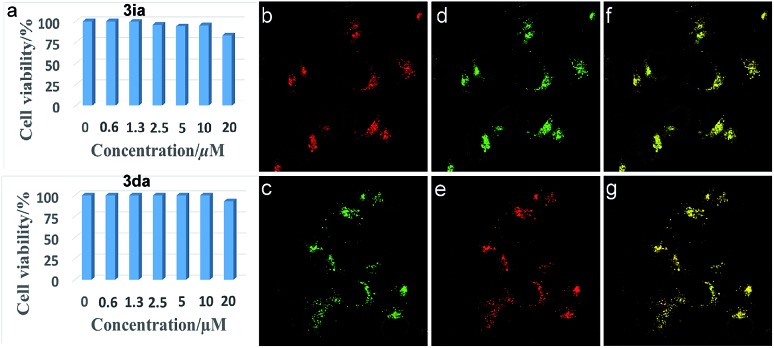
(a) Cell viability values (%) estimated by a CellTiler 96®AQueous One Solution Cell Proliferation Assay employing HepG2 cells, stained with 0–20 μM of **3ia** and **3da**, at 37 °C for 24 h. (b) and (c) Fluorescent images of HepG2 cells cultured with **3ia** (1.0 μM, *λ*_ex_ = 552 nm, *λ*_em_ = 550–650 nm) (top) and **3da** (1.0 μM, *λ*_ex_ = 488 nm, *λ*_em_ = 500–600 nm) (bottom). (d) and (e) Fluorescent images of HepG2 cells cultured with commercially available lysosome-targeted trackers, LTG (1.0 μM, *λ*_ex_ = 488 nm, *λ*_em_ = 460–560 nm) (top) and LTR (1.0 μM, *λ*_ex_ = 546 nm, *λ*_em_ = 550–650 nm) (bottom). (f) and (g) Merged images of (b and d) (top) and (c and e) (bottom).

Inspired by the potential biological applications of the carbocations,^1^–^3^,^43^ cytotoxicity experiments of **3ia** and **3da** were conducted and both exhibited almost no toxicity to cultured HepG2 cells as shown in Fig. 2a. Even with the higher concentration of **3ia** or **3da** at 20 μM, little variation of cell viability was detected. Subcellular localization experiments disclosed that both **3ia** and **3da** had the ability to specifically target lysosomes, which are the primary digestive component of the cell (Fig. 2b and c). The Pearson’s coefficient (*R*_r_ = 0.93 and 0.96, respectively), calculated using Image-Pro Plus software, further demonstrated the highly specific accumulation of **3ia** and **3da** in the lysosomes of living cells.

## Conclusions

In summary, on the basis of the conception of heteroatom-promoted delocalization of the positive charge of oxonium, we have developed a highly efficient rhodium(iii)-catalyzed hydration and three fold C–H activation/annulation cascade of arylnitriles with alkynes, which rapidly assembles a large library of stable delocalized carbocations. This protocol enables a good tolerance of sensitive yet synthetically useful functional groups such as halide, aldehyde, ketone, cyano, ester and nitro. Because both arylnitriles and alkynes are structurally diverse and easily available, the structures of the delocalized carbocations are readily amenable to chemical modification, and their properties are tailored handily by the option of substituent variation. These cations exhibit tunable fluorescence and low cytotoxicity, and are able to be localized in lysosomes. The rapid gateway toward stable delocalized carbocations developed herein has exemplified the power of C–H activation in the discovery of new organic functional materials. Future work in our laboratory will focus on the highly specific targeting mechanism for lysosomes and on the development of more diverse PAH carbocations.

## Conflicts of interest

There are no conflicts to declare.

## Supplementary Material

Supplementary information

Crystal structure data
