# Supplementary material for: Annulation cascade of arylnitriles with alkynes to stable delocalized PAH carbocations via intramolecular rhodium migration
Source: Chem Sci. 2018 May 29;9(24):5488–93. doi: 10.1039/c8sc01963k (PMC6009538; doi:10.1039/c8sc01963k)

Electronic Supplementary Material (ESI) for Chemical Science.
This journal is © The Royal Society of Chemistry 2018

Supporting Information

Annulation Cascade of Arylnitriles with Alkynes to Stable Delocalized PAH Carbocations via Intramolecular Rhodium Migration

Jiangliang Yin,[a] Fulin Zhou,[a] Lei Zhu,[b] Mufan Yang,[a] Yu Lan,[b] and Jingsong You*[a]

[a]Key Laboratory of Green Chemistry and Technology of Ministry of Education, College of Chemistry, Sichuan University, 29 Wangjiang Road, Chengdu 610064, PR China.

[b]School of Chemistry and Chemical Engineering, Chongqing University, Chongqing 400030, PR China

*e-mail: jsyou@scu.edu.cn

Table of Contents

I. General remarks S3

II. Optimization of the oxidative annulation of arylnitriles with alkynes S3

III. General procedure for the synthesis of delocalized carbocation..........................S5

IV. General procedure for the preparation of aryl nitriles …………………..….…..…... S5

V. General procedure for the preparation of alkynes ……………….…………..…. S6

VI. Preparation and characterization of the described substances S7

VII. Representative experiments on a 1.0 mmol scale ………….....................…… S24

VIII. Mechanistic study ……………………………………...…………….…..……. S24

IX. Photophysical properties of the representative products ………..…...………. S30

X. Cytotoxicity assay and cell imaging experiment …………..……..…….……. S35

XI. Single crystal X-ray structures of **3ea** and **3ia** …………………..……..…....….. S39

XII. DFT calculation ………………………..…………………..………..……...S42

XIII. References ……………………………………..………………......……. S44

XIV. Copies of NMR spectra …………………………………………………..…… S46

I. General remarks

NMR spectra were recorded on a Varian Inova 400 spectrometer. The ^{1}H NMR (400 MHz) chemical shifts were recorded relative to $CDCl_3$ or CD_3CN as the internal reference ($CDCl_3$: δ_H = 7.26 ppm; CD_3CN: δ_H = 1.94 ppm). The ^{13}C NMR (100 MHz) chemical shifts were given using $CDCl_3$ or CD_3CN as the internal standard ($CDCl_3$: δ_C = 77.16 ppm; CD_3CN: δ_C = 118.26 ppm). High-resolution mass spectra (HRMS) were obtained with a Shimadzu LCMS-IT-TOF (ESI) or a Waters-Q-TOF-Premier (ESI). X-Ray single-crystal diffraction data were collected on an Oxford Xcalibur E single crystal diffractometer. UV/Vis spectra experiments were conducted on a HITACHI U-2910. Absolute quantum yields and fluorescence spectra were collected on a Horiba Jobin Yvon-Edison Fluoromax-4 fluorescence spectrometer with a calibrated integrating sphere system. The HepG2 (human hepatoma cell line) was purchased from Shanghai Institute of Biochemistry and Cell Biology, Chinese Academy of Sciences. The confocal imaging measurements were conducted on a LSM 780 (Zeiss) confocal fluorescent microscope.

Unless otherwise noted, all reagents were obtained from commercial suppliers and used without further purification. $[Cp^*RhCl_2]_2$[1] were prepared according to the literature procedures. The solvents were purified and dried using an Innovative Technology PS-MD-5 Solvent Purification System. $RhCl_3 \cdot xH_2O$ were purchased from Shanxi Kaida Chemical Engineering (China) CO., Ltd. $AgSbF_6$ was purchased from Alfa Aesar.

II. Optimization of the oxidative annulation of arylnitriles with alkynes

A Schlenk tube with a magnetic stir bar was charged with metal complex (5.0 μmol, 5.0 mol %), $AgSbF_6$ (20 μmol, 20 mol %, if required), oxidant, additives, benzonitrile (30.9 mg, 0.3 mmol), diphenylacetylene (53.5 mg, 0.3 mmol), and solvent (0.5 mL) under an N_2 atmosphere. The resulting solution was stirred at room temperature for 10 min and then at the indicated temperature for 12 h. Subsequently, it was diluted with 10 mL of dichloromethane. The mixture was evaporated under reduced pressure and

the residue was absorbed into small amounts of silica gel. Purification was performed by column chromatography on silica gel (dichloromethane/ethyl acetate = 20:1, v/v, then dichloromethane/methanol = 20:1, v/v) to provide **3aa**.

Table S1. **Optimization for the synthesis of delocalized carbocation**[a]

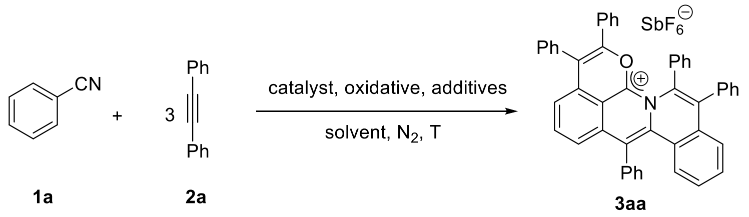

Entry	Metal complex	Oxidant (equiv)	Additive **1** (equiv)	Additive **2** (equiv)	Solvent (mL)	Yield[b] (%)
1	$[Cp^*RhCl_2]_2$	AgOAc (3.0)	-	CH_3COOH (6.0)	DCE (0.5)	7
2	$[Cp^*RhCl_2]_2$	AgOAc (3.0)	$NaSbF_6$ (2.0)	CH_3COOH (6.0)	DCE (0.5)	39
3	$[Cp^*RhCl_2]_2$	AgOAc (3.0)	$NaSbF_6$ (2.0)	CH_3COOH (8.0)	DCE (0.5)	21
4	$[Cp^*RhCl_2]_2$	AgOAc (3.0)	$NaSbF_6$ (2.0)	CH_3COOH/H_2O (6.0/8.0)	DCE (0.5)	45
5	$[Cp^*RhCl_2]_2$	AgOAc (3.0)	$NaSbF_6$ (2.0)	CH_3COOH/H_2O (6.0/10.0)	DCE (0.5)	43
6	$[Cp^*RhCl_2]_2$	AgOAc (3.0)	$NaSbF_6$ (2.0)	CH_3COOH/H_2O (6.0/8.0)	toluene (0.5)	25
7	$[Cp^*RhCl_2]_2$	AgOAc (3.0)	$NaSbF_6$ (2.0)	CH_3COOH/H_2O (6.0/8.0)	1,4-dioxane (0.5)	trace
8	$[Cp^*RhCl_2]_2$	AgOAc (3.0)	$NaSbF_6$ (2.0)	CH_3COOH/H_2O (6.0/8.0)	THF (0.5)	-
9	$[Cp^*RhCl_2]_2$	$Cu(OAc)_2$ (3.0)	$NaSbF_6$ (2.0)	CH_3COOH/H_2O (6.0/8.0)	DCE (0.5)	32
10	$[Cp^*RhCl_2]_2$	Ag_2O (3.0)	$NaSbF_6$ (2.0)	CH_3COOH/H_2O (6.0/8.0)	DCE (0.5)	79
11[c]	$[Cp^*RhCl_2]_2$	Ag_2O (3.0)	$NaSbF_6$ (2.0)	CH_3COOH/H_2O (6.0/8.0)	DCE (0.5)	88

12[c]	$[Cp^*RhCl_2]_2$	Ag_2O (3.0)	$NaSbF_6$ (1.5)	CH_3COOH/H_2O (6.0/8.0)	DCE (0.5)	86
13	$[Cp^*RhCl_2]_2$	Ag_2O (2.5)	$NaSbF_6$ (1.5)	CH_3COOH/H_2O (6.0/8.0)	DCE (0.5)	80
14[d]	$[Cp^*RhCl_2]_2$	Ag_2O (3.0)	$NaSbF_6$ (1.5)	CH_3COOH/H_2O (6.0/8.0)	DCE (0.5)	83
15	$[Cp^*IrCl_2]_2$	Ag_2O (3.0)	$NaSbF_6$ (1.5)	CH_3COOH/H_2O (6.0/8.0)	DCE (0.5)	ND
16	$[\{RuCl_2(p\text{-cymene})\}_2]$	Ag_2O (3.0)	$NaSbF_6$ (1.5)	CH_3COOH/H_2O (6.0/8.0)	DCE (0.5)	ND

[a] Reaction conditions: **1a** (0.3 mmol), **2a** (0.3 mmol), $[Cp^*RhCl_2]_2$ (5 mol %), $AgSbF_6$ (20 mol %), oxidant (x equiv), additives (x equiv), and solvent at 120 °C under N_2 for 12 h. [b] Isolated yield. [c] Benzonitrile was used with 0.2 mmol. [d] The reaction was carried out for 18 h.

III. General procedure for the synthesis of delocalized carbocation

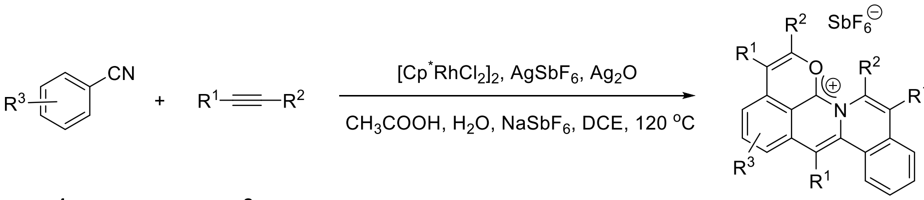

1 **2** **3**

A Schlenk tube with a magnetic stir bar was charged with $AgSbF_6$ (6.9 mg, 20 μmol), $NaSbF_6$ (38.9 mg, 0.15 mmol), $[Cp^*RhCl_2]_2$ (3.1 mg, 5.0 μmol), Ag_2O (69.5 mg, 0.30 mmol), CH_3COOH (36 μL, 0.6 mmol), H_2O (14.4 μL, 0.8 mmol), arylnitrile (0.20 mmol), alkyne (0.30 mmol), and DCE (0.5 mL) under an N_2 atmosphere. The resulting mixture was stirred at room temperature for 10 min and then at the indicated temperature for appropriate time. Subsequently, it was diluted with 10 mL of dichloromethane. The mixture was evaporated under reduced pressure and the residue was absorbed into small amounts of silica gel. The purification was performed by column chromatography on silica gel to provide the desired product **3**.

IV. General procedure for the preparation of aryl nitriles

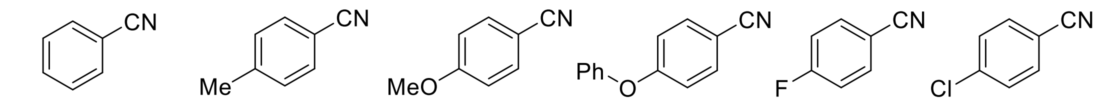

1a **1b** **1c** **1d** **1e** **1f**

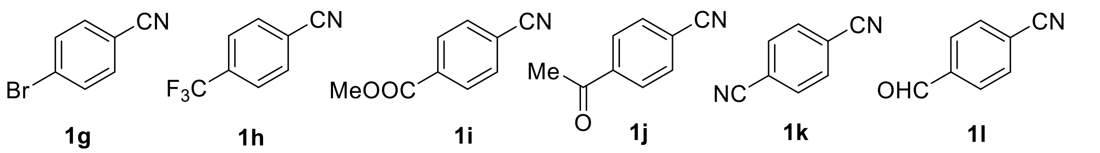

1g **1h** **1i** **1j** **1k** **1l**

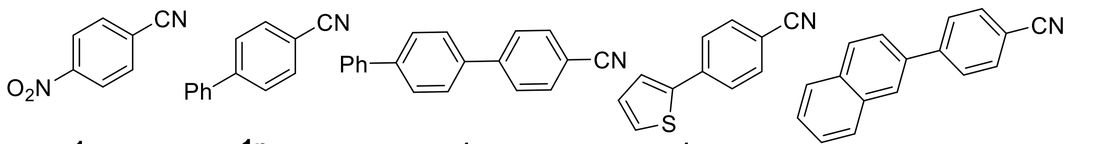

1m **1n** **1o** **1p** **1q**

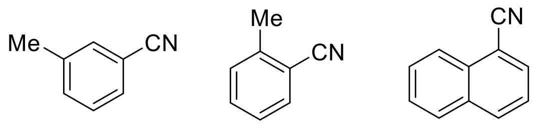

1r **1s** **1t**

Aryl nitriles **1a**, **1b, 1c, 1d, 1e, 1f, 1g, 1h, 1i, 1j, 1k, 1l, 1m, 1r, 1s, 1t** were purchased from the commercial suppliers and used without any further purification. The preparation of aryl nitriles **1n, 1o, 1p** and **1q** was conducted as the following procedure.[2]

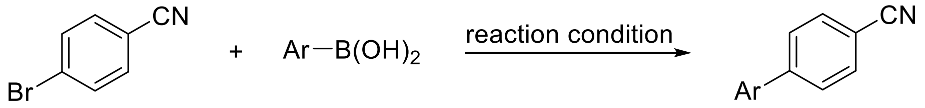

A Schlenk tube with a magnetic stir bar was charged with $Pd_2(dba)_3$ (9.6 mg, 0.01 mmol), $P^tBu_3BF_4$ (12.2 mg, 0.04 mmol), Na_2CO_3 (1.0 mol/L in water, 1.8 mL), 4-bromobenzonitrile (127.4 mg, 0.7 mmol), arylboronic acid (1.4 mmol), and dry toluene (2.0 mL) under an N_2 atmosphere. The resulting mixture was stirred at 100 ^oC for 12 h. Purification was conducted by column chromatography on silica gel to provide the desired products **1n**, **1o**, **1p** and **1q**.

V. General procedure for the preparation of alkynes

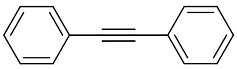

2a

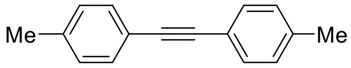

2b

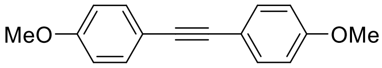

2c

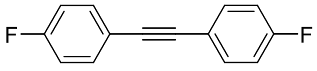

2d

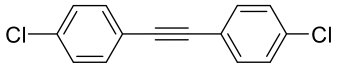

2e

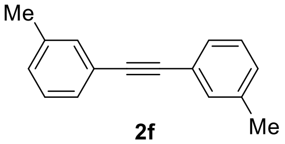

2f

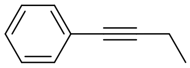

2g

Alkynes **2a** and **2g** were purchases from the commercial suppliers directly and were used without any further purification. Alkynes **2b**, **2c**, **2d**, **2e** and **2f** were synthesized by following the literature procedures.[3]

VI. Preparation and characterization of the described substances

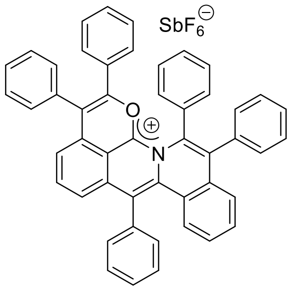

2,3,7,12,13-Pentaphenylisoquinolino[2,1-*b*]pyrano[4,3,2-*ij*]isoquinolin-1-ium hexafluoroantimonate (3aa)

Following the general procedure. Benzonitrile (20.6 mg, 0.2 mmol) and diphenylacetylene (53.5 mg, 0.3 mmol) were used. Purification via column chromatography on silica gel (dichloromethane/ethyl acetate = 10:1, v/v, then dichloromethane/methanol = 20:1, v/v) afforded **3aa** as an orange solid (76.2 mg, 86% yield). ^{1}H NMR ($CDCl_3$, 400 MHz): δ (ppm) 8.00 (t, J = 7.6 Hz, 1H), 7.66-7.63 (m, 6H), 7.45 (d, J = 8.0 Hz, 1H), 7.38-7.27 (m, 9H), 7.22-7.20 (m, 5H), 7.13-7.08 (m, 3H), 6.97 (d, J = 6.4 Hz, 2H), 6.85 (d, J = 7.2 Hz, 2H), 6.72-6.70 (m, 3H). ^{13}C NMR ($CDCl_3$, 100 MHz): δ (ppm) 155.9, 152.2, 138.4, 137.8, 136.0, 135.6, 135.3, 134.3, 134.1, 133.2, 131.51, 131.48, 131.1, 130.85, 130.79, 130.6, 130.5, 130.02, 130.00, 129.9, 129.6, 129.4, 129.1, 128.8, 128.6, 128.5, 128.4, 128.2, 128.0, 127.8, 127.5,

127.4, 126.0, 124.2, 122.4, 122.2, 115.0. HRMS (ESI^+): calcd for $C_{49}H_{32}NO^+$, 650.2478; found 650.2470.

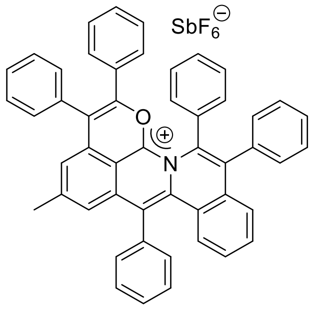

5-Methyl-2,3,7,12,13-pentaphenylisoquinolino[2,1-*b*]pyrano[4,3,2-*ij*]isoquinolin-1-ium hexafluoroantimonate (3ba)

Following the general procedure. 4-Methylbenzonitrile (23.4 mg, 0.2 mmol) and diphenylacetylene (53.5 mg, 0.3 mmol) were used. Purification via column chromatography on silica gel (dichloromethane/ethyl acetate = 10:1, v/v, then dichloromethane/methanol = 20:1, v/v) afforded **3ba** as a brown solid (52.7 mg, 59% yield). ^{1}H NMR ($CDCl_3$, 400 MHz): δ (ppm) 7.71-7.64 (m, 5H), 7.41-7.37 (m, 6H), 7.33-7.27 (m, 4H), 7.21-7.19 (m, 5H), 7.12-7.07 (m, 4H), 6.96 (d, J = 7.2 Hz, 2H), 6.81 (d, J = 7.6 Hz, 2H), 6.76-6.70 (m, 3H), 2.50 (s, 3H). ^{13}C NMR ($CDCl_3$, 100 MHz): δ (ppm) 155.6, 152.3, 151.4, 137.9, 136.0, 135.6, 135.1, 134.3, 134.2, 133.8, 133.2, 131.6, 131.5, 131.2, 130.8, 130.74, 130.66, 130.5, 130.0, 129.8, 129.7, 129.5, 129.4, 129.1, 128.8, 128.52, 128.47, 128.44, 128.1, 128.0, 127.7, 127.3, 126.8, 126.1, 124.3, 123.8, 121.9, 113.5, 23.8. HRMS (ESI^+): calcd for $C_{50}H_{34}NO^+$, 664.2635; found 664.2632.

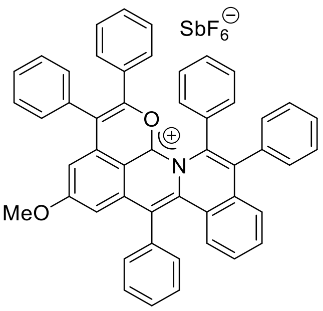

5-Methoxy-2,3,7,12,13-pentaphenylisoquinolino[2,1-*b*]pyrano[4,3,2-*ij*]isoquinolin-1-ium hexafluoroantimonate (3ca)

Following the general procedure. 4-Methoxybenzonitrile (26.6 mg, 0.2 mmol) and diphenylacetylene (53.5 mg, 0.3 mmol) were used. Purification via column chromatography on silica gel (dichloromethane/ethyl acetate = 10:1, v/v, then dichloromethane/methanol = 20:1, v/v) afforded **3ca** as a brown solid (43.0 mg, 47% yield). ^{1}H NMR ($CDCl_3$, 400 MHz): δ (ppm) 7.69-7.64 (m, 5H), 7.40-7.35 (m, 5H), 7.32-7.29 (m, 2H), 7.28-7.26 (m, 2H), 7.21-7.19 (m, 4H), 7.11-7.06 (m, 4H), 6.98-6.96 (m, 3H), 6.82-6.73 (m, 6H), 3.80 (s, 3H). ^{13}C NMR ($CDCl_3$, 100 MHz): δ (ppm) 168.2, 154.6, 152.3, 140.7, 137.6, 136.1, 136.0, 134.3, 134.2, 133.3, 133.0, 131.7, 131.4, 131.0, 130.9, 130.8, 130.7, 130.4, 130.0, 129.72, 129.65, 129.5, 129.4, 129.1, 128.8, 128.4, 128.1, 128.0, 127.7, 127.2, 126.1, 125.893, 125.889, 121.5, 111.6, 110.4, 110.1, 107.1, 56.4. HRMS (ESI$^+$): calcd for $C_{50}H_{34}NO_2^+$, 680.2584; found 680.2580.

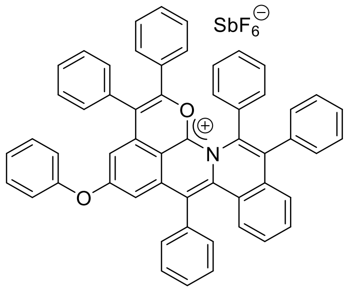

5-Phenoxy-2,3,7,12,13-pentaphenylisoquinolino[2,1-*b*]pyrano[4,3,2-*ij*]isoquinolin-1-ium hexafluoroantimonate (3da)

Following the general procedure. 4-Phenoxybenzonitrile (39.0 mg, 0.2 mmol) and diphenylacetylene (53.5 mg, 0.3 mmol) were used. Purification via column chromatography on silica gel (dichloromethane/ethyl acetate = 10:1, v/v, then dichloromethane/methanol = 20:1, v/v) afforded **3da** as a yellow solid (85.6 mg, 87% yield). ^{1}H NMR (CD_3CN, 400 MHz): δ (ppm) 7.63-7.60 (m, 5H), 7.47-7.42 (m, 4H), 7.40-7.38 (m, 3H), 7.34-7.31 (m, 4H), 7.29-7.26 (m, 4H), 7.21-7.19 (m, 2H), 7.18-7.14 (m, 3H), 7.12-7.10 (m, 4H), 6.90 (d, J = 2.0 Hz, 1H), 6.86-6.84 (m, 2H), 6.81 (d, J = 2.0 Hz, 1H), 6.79-6.77 (m, 1H), 6.73-6.69 (m, 2H). ^{13}C NMR ($CDCl_3$, 100 MHz): δ (ppm) 167.5, 155.5, 154.4, 153.1, 141.1, 139.2, 137.4, 136.8, 135.6, 135.0, 134.4, 133.8, 132.9, 132.3, 131.9, 131.7, 131.5, 131.4, 131.3, 131.09, 131.07,

130.63, 130.59, 130.4, 130.2, 130.1, 130.0, 129.20, 129.18, 129.0, 128.9, 128.6, 128.5, 127.8, 127.0, 126.9, 126.6, 122.0, 121.5, 112.6, 111.7, 109.9. HRMS (ESI$^+$): calcd for $C_{55}H_{36}NO_2^+$, 742.2741; found 742.2742.

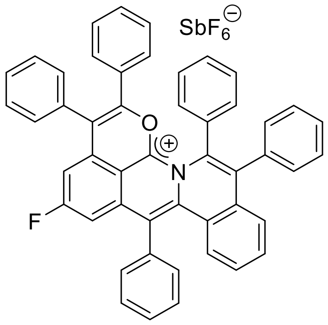

5-Fluoro-2,3,7,12,13-pentaphenylisoquinolino[2,1-*b*]pyrano[4,3,2-*ij*]isoquinolin-1-ium hexafluoroantimonate (3ea)

Following the general procedure. 4-Fluorobenzonitrile (24.2 mg, 0.2 mmol) and diphenylacetylene (53.5 mg, 0.3 mmol) were used. Purification via column chromatography on silica gel (dichloromethane/ethyl acetate = 10:1, v/v, then dichloromethane/methanol = 20:1, v/v) afforded **3ea** as an orange solid (82.0 mg, 91% yield). ^{1}H NMR ($CDCl_3$, 400 MHz): δ (ppm) 7.69-7.66 (m, 5H), 7.46 (d, J = 8.4 Hz, 1H), 7.41-7.33 (m, 5H), 7.29-7.27 (m, 3H), 7.24-7.21 (m, 6H), 7.14-7.07 (m, 3H), 6.97-6.95 (m, 3H), 6.85 (d, J = 7.6 Hz, 2H), 6.74-6.68 (m, 3H). ^{13}C NMR ($CDCl_3$, 100 MHz): δ (ppm) 168.9 (d, J_{C-F} = 260.3 Hz), 155.2, 153.2, 141.2, 141.1, 139.5, 139.3, 136.6, 135.9, 134.2, 134.1, 134.0, 133.2, 131.7, 131.1 (d, J_{C-F} = 1.9 Hz), 130.95, 130.94, 130.6, 130.4, 130.2 (d, J_{C-F} = 8.5 Hz), 129.9, 129.62, 129.56, 129.3 (d, J_{C-F} = 7.8 Hz), 128.8, 128.6, 128.44 (d, J_{C-F} = 2.0 Hz), 128.4, 128.1, 128.0, 127.8, 127.3, 126.7 (d, J_{C-F} = 5.0 Hz), 125.64, 121.76, 121.73, 112.47, 111.1 (d, J_{C-F} = 27.0 Hz), 110.1 (d, J_{C-F} = 25.6 Hz). ^{19}F NMR ($CDCl_3$, 400 MHz): δ (ppm) -89.99–-90.05 (m), -107.76– -107.83 (m), -154.28, -154.33. HRMS (ESI$^+$): calcd for $C_{49}H_{31}FNO^+$, 668.2384; found 668.2381.

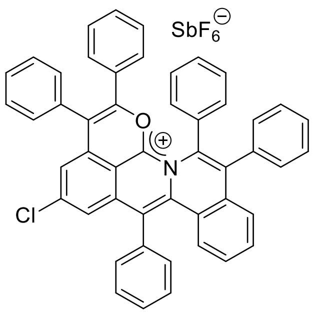

5-Chloro-2,3,7,12,13-pentaphenylisoquinolino[2,1-*b*]pyrano[4,3,2-*ij*]isoquinolin-1-ium hexafluoroantimonate (3fa)

Following the general procedure. 4-Chlorobenzonitrile (27.5 mg, 0.2 mmol) and diphenylacetylene (53.5 mg, 0.3 mmol) were used. Purification via column chromatography on silica gel (dichloromethane/ethyl acetate = 10:1, v/v, then dichloromethane/methanol = 20:1, v/v) afforded **3fa** as an orange solid (87.6 mg, 95% yield). ^{1}H NMR (CDCl$_3$, 400 MHz): δ (ppm) 7.67-7.65 (m, 5H), 7.51 (s, 1H), 7.44-7.35 (m, 9H), 7.20-7.19 (m, 6H), 7.12-7.06 (m, 3H), 6.95 (d, *J* = 6.0 Hz, 2H), 6.83 (d, *J* = 6.8 Hz, 2H), 6.71-6.69 (m, 3H). ^{13}C NMR (CDCl$_3$, 100 MHz): δ (ppm) 155.4, 153.3, 146.1, 138.9, 137.0, 136.8, 135.6, 134.3, 134.1, 134.0, 133.2, 131.6, 131.1, 131.0, 130.9, 130.6, 130.4, 130.2, 130.1, 129.8, 129.5, 129.4, 129.2, 128.8, 128.6, 128.5, 128.4, 128.1, 128.0, 127.7, 127.3, 126.4, 125.7, 123.3, 122.3, 121.3, 113.4. HRMS (ESI$^+$): calcd for $C_{49}H_{31}ClNO^+$, 684.2089; found 684.2093.

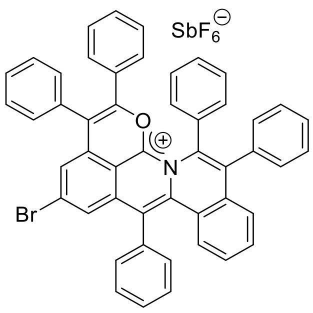

5-Bromo-2,3,7,12,13-pentaphenylisoquinolino[2,1-*b*]pyrano[4,3,2-*ij*]isoquinolin-1-ium hexafluoroantimonate (3ga)

Following the general procedure. 4-Bromobenzonitrile (36.4 mg, 0.2 mmol) and diphenylacetylene (53.5 mg, 0.3 mmol) were used. Purification via column chromatography on silica gel (dichloromethane/ethyl acetate = 10:1, v/v, then

dichloromethane/methanol = 20:1, v/v) afforded **3ga** as an orange solid (90.4 mg, 94% yield). ^{1}H NMR ($CDCl_3$, 400 MHz): δ (ppm) 7.68-7.65 (m, 6H), 7.43-7.34 (m, 7H), 7.26-7.19 (m, 8H), 7.12-7.06 (m, 3H), 6.95 (d, J = 6.4 Hz, 2H), 6.83 (d, J = 7.2 Hz, 2H), 6.71-6.69 (m, 3H). ^{13}C NMR ($CDCl_3$, 100 MHz): δ (ppm) 155.5, 153.3, 138.7, 136.9, 136.7, 135.6, 135.3, 134.4, 134.1, 134.0, 133.2, 131.7, 131.1, 131.02, 130.96, 130.94, 130.6, 130.4, 130.2, 129.9, 129.6, 129.4, 129.3, 128.8, 128.6, 128.5, 128.4, 128.2, 128.0, 127.7, 127.3, 126.4, 126.2, 125.8, 125.1, 121.1, 113.7. HRMS (ESI$^+$): calcd for $C_{49}H_{31}BrNO^+$, 728.1584; found 728.1576.

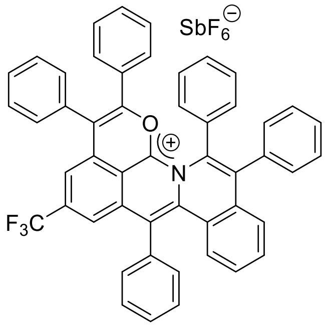

2,3,7,12,13-Pentaphenyl-5-(trifluoromethyl)isoquinolino[2,1-*b*]pyrano[4,3,2-*ij*]isoquinolin-1-ium hexafluoroantimonate (3ha)

Following the general procedure. 4-(Trifluoromethyl)benzonitrile (34.2 mg, 0.2 mmol) and diphenylacetylene (53.5 mg, 0.3 mmol) were used. Purification via column chromatography on silica gel (dichloromethane/ethyl acetate = 10:1, v/v, then dichloromethane/methanol = 20:1, v/v) afforded **3ha** as a red solid (88.6 mg, 93% yield). ^{1}H NMR ($CDCl_3$, 400 MHz): δ (ppm) 7.93 (d, J = 8.0 Hz, 1H), 7.80 (s, 1H), 7.69-7.67 (m, 6H), 7.49 (d, J = 8.4 Hz, 1H), 7.44-7.37 (m, 6H), 7.27-7.21 (m, 6H), 7.16-7.08 (m, 3H), 6.96 (d, J = 6.8 Hz, 2H), 6.88 (d, J = 7.2 Hz, 2H), 6.75-6.68 (m, 3H). ^{13}C NMR ($CDCl_3$, 100 MHz): δ (ppm) 168.0, 155.5, 153.5, 138.6 (q, J = 33.0 Hz), 138.1, 137.08, 136.8, 136.7, 135.5, 135.2, 134.0, 133.9, 133.2, 131.6, 131.3, 131.0, 130.98, 130.8, 130.5, 130.4, 130.3, 129.9, 129.7, 129.4, 129.3, 128.8, 128.6, 128.4, 128.3, 128.1, 128.0, 127. 8, 127.4, 125.7 (q, J = 3.7 Hz), 122.8 (q, J = 272.9 Hz), 121.90, 120.4 (q, J = 4.5 Hz), 117.5 (q, J = 3.4 Hz), 116.0. ^{19}F NMR ($CDCl_3$, 400 MHz): δ (ppm) -63.00, -63.42, -154.14, -154.19. HRMS (ESI$^+$): calcd for $C_{50}H_{31}F_3NO^+$, 718.2352; found 718.2355.

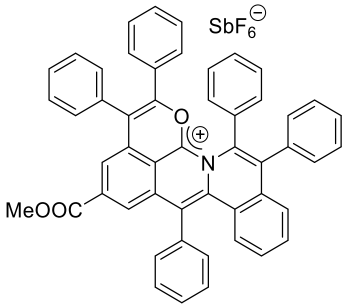

5-(Methoxycarbonyl)-2,3,7,12,13-pentaphenylisoquinolino[2,1-*b*]pyrano[4,3,2-*ij*]isoquinolin-1-ium hexafluoroantimonate (3ia)

Following the general procedure. Methyl 4-cyanobenzoate (32.2 mg, 0.2 mmol) and diphenylacetylene (53.5 mg, 0.3 mmol) were used. Purification via column chromatography on silica gel (dichloromethane/ethyl acetate = 10:1, v/v, then dichloromethane/methanol = 20:1, v/v) afforded **3ia** as a red solid (90.5 mg, 96% yield). ^{1}H NMR ($CDCl_3$, 400 MHz): δ (ppm) 8.24 (s, 1H), 7.82 (s, 1H), 7.67 (m, 5H), 7.44-7.20 (m, 14H), 7.13-7.05 (m, 3H), 6.95 (d, J = 6.8 Hz, 2H), 6.83 (d, J = 7.2 Hz, 2H), 6.73-6.66 (m, 3H), 3.85 (s, 3H). ^{13}C NMR ($CDCl_3$, 100 MHz): δ (ppm) 165.3, 155.6, 152.9, 138.2, 137.9, 136.4, 135.9, 135.7, 134.9, 134.1, 133.9, 133.2, 131.5, 131.12, 131.07, 130.9, 130.6, 130.5, 130.2, 130.1, 129.9, 129.6, 129.51, 129.48, 129.2, 128.8, 128.7, 128.5, 128.4, 128.3, 128.2, 128.0, 127.7, 127.3, 125.9, 125.1, 122.3, 121.7, 116.4, 53.3. HRMS (ESI$^+$): calcd for $C_{51}H_{34}NO_3^+$, 708.2533; found 708.2530.

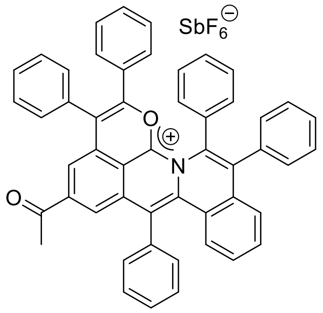

5-Acetyl-2,3,7,12,13-pentaphenylisoquinolino[2,1-*b*]pyrano[4,3,2-*ij*]isoquinolin-1-ium hexafluoroantimonate (3ja)

Following the general procedure. 4-Acetylbenzonitrile (29.0 mg, 0.2 mmol) and diphenylacetylene (53.5 mg, 0.3 mmol) were used. Purification via column chromatography on silica gel (dichloromethane/ethyl acetate = 10:1, v/v, then

dichloromethane/methanol = 20:1, v/v) afforded **3ja** as a red solid (57.0 mg, 61% yield). ^{1}H NMR ($CDCl_3$, 400 MHz): δ (ppm) 8.06 (s, 1H), 7.70-7.69 (m, 6H), 7.47 (d, J = 8.4 Hz, 1H), 7.43-7.36 (m, 6H), 7.29-7.28 (m, 2H), 7.23-7.20 (m, 5H), 7.14 (t, J = 7.8 Hz, 1H), 7.09 (t, J = 7.8 Hz, 2H), 6.97 (d, J = 6.8 Hz, 2H), 6.84 (d, J = 8.0 Hz, 2H), 6.74-6.69 (m, 3H), 2.49 (s, 3H). ^{13}C NMR ($CDCl_3$, 100 MHz): δ (ppm) 197.4, 155.6, 152.7, 144.1, 138.3, 136.2, 136.1, 135.7, 134.9, 134.1, 133.9, 133.4, 131.5, 131.2, 131.1, 130.9, 130.6, 130.5, 130.3, 130.1, 129.9, 129.53, 129.51, 129.3, 128.8, 128.7, 128.6, 128.5, 128.45, 128.2, 128.1, 127.7, 127.4, 125.9, 123.7, 122.5, 120.3, 116.3, 27.2. HRMS (ESI$^+$): calcd for $C_{51}H_{34}NO_2^+$, 692.2584; found 692.2584.

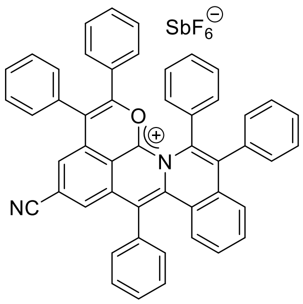

5-Cyano-2,3,7,12,13-pentaphenylisoquinolino[2,1-*b*]pyrano[4,3,2-*ij*]isoquinolin-1-ium hexafluoroantimonate (3ka)

Following the general procedure. Terephthalonitrile (25.6 mg, 0.2 mmol), diphenylacetylene (53.5 mg, 0.3 mmol) were used. Purification via column chromatography on silica gel (dichloromethane/ethyl acetate = 10:1, v/v, then dichloromethane/methanol = 20:1, v/v) afforded **3ka** as a red solid (80.0 mg, 88% yield). ^{1}H NMR ($CDCl_3$, 400 MHz): δ (ppm) 7.79 (s, 1H), 7.70-7.63 (m, 5H), 7.50 (d, J = 8.8 Hz, 1H), 7.44-7.33 (m, 6H), 7.29-7.28 (m, 3H), 7.23-7.13 (m, 6H), 7.09 (t, J = 7.6 Hz, 2H), 6.95 (d, J = 7.2 Hz, 2H), 6.86 (d, J = 7.6 Hz, 2H), 6.76-6.67 (m, 3H). ^{13}C NMR ($CDCl_3$, 100 MHz): δ (ppm) 155.3, 153.7, 137.8, 137.3, 136.6, 135.37. 135.35, 134.0, 133.7, 133.3, 131.6, 131.5, 131.1, 130.9, 130.6, 130.5, 130.43, 130.36, 130.34, 129.9, 129.7, 129.4, 129.2, 128.9, 128.8, 128.6, 128.5, 128.3, 128.1, 127.8, 127.47, 127.46, 125.6, 122.7, 121.5, 120.7, 117.5, 115.8, 110.1. HRMS (ESI$^+$): calcd for $C_{50}H_{31}N_2O^+$, 675.2431; found 675.2430.

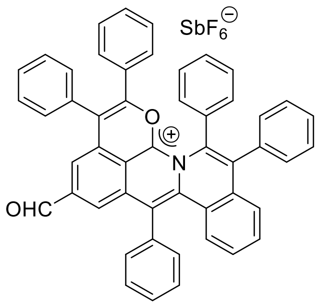

5-Formyl-2,3,7,12,13-pentaphenylisoquinolino[2,1-*b*]pyrano[4,3,2-*ij*]isoquinolin-1-ium hexafluoroantimonate (3la)

Following the general procedure. 4-Formylbenzonitrile (26.2 mg, 0.2 mmol) and diphenylacetylene (53.5 mg, 0.3 mmol) were used. Purification via column chromatography on silica gel (dichloromethane/ethyl acetate = 10:1, v/v, then dichloromethane/methanol = 20:1, v/v) afforded **3la** as a red solid (58.9 mg, 64% yield). ^{1}H NMR ($CDCl_3$, 400 MHz): δ (ppm) 10.04 (s, 1H), 8.03 (s, 1H), 7.71-7.66 (m, 5H), 7.50 (d, J = 8.4 Hz, 1H), 7.45-7.37 (m, 6H), 7.32-7.29 (m, 3H), 7.22-7.08 (m, 8H), 6.97 (d, J = 6.8 Hz, 2H), 6.86 (d, J = 7.2 Hz, 2H), 6.78-6.71 (m, 3H). ^{13}C NMR ($CDCl_3$, 100 MHz): δ (ppm) 191.1, 155.7, 153.1, 142.0, 138.6, 136.7, 136.6, 135.7, 135.2, 134.1, 134.0, 133.4, 131.6, 131.3, 131.1, 131.0, 130.6, 130.5, 130.4, 130.3, 130.2, 129.9, 129.7, 129.6, 129.5, 129.3, 128.9, 128.8, 128.60, 128.55, 128.47, 128.3, 128.1, 127.8, 127.4, 126.1, 125.9, 122.5, 120.1, 117.1. HRMS (ESI$^+$): calcd for $C_{50}H_{32}NO_2^+$, 678.2428; found 678.2429.

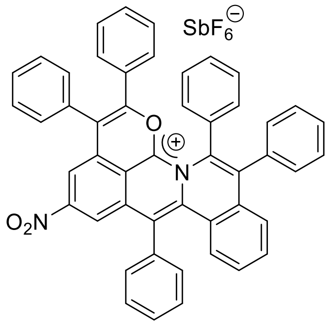

5-Nitro-2,3,7,12,13-pentaphenylisoquinolino[2,1-*b*]pyrano[4,3,2-*ij*]isoquinolin-1-ium hexafluoroantimonate (3ma)

Following the general procedure. 4-Nitrobenzonitrile (29.6 mg, 0.2 mmol) and diphenylacetylene (53.5 mg, 0.3 mmol) were used. Purification via column

chromatography on silica gel (dichloromethane/ethyl acetate = 10:1, v/v, then dichloromethane/methanol = 20:1, v/v) afforded **3ma** as a red solid (40.5 mg, 43% yield). ^{1}H NMR (CDCl$_3$, 400 MHz): δ (ppm) 8.33 (s, 1H), 7.88 (s, 1H), 7.69-7.67 (m, 5H), 7.51 (d, J = 8.4 Hz, 1H), 7.44-7.36 (m, 6H), 7.27-7.13 (m, 8H), 7.09 (t, J = 7.2 Hz, 2H), 6.95 (d, J = 7.2 Hz, 2H), 6.86 (d, J = 7.6 Hz, 2H), 6.74-6.68 (m, 3H). ^{13}C NMR (CDCl$_3$, 100 MHz): δ (ppm) 155.4, 153.9, 153.1, 138.9, 138.0, 137.5, 135.6, 135.4, 134.0, 133.7, 133.4, 131.7, 131.5, 131.1, 131.0, 130.7, 130.52, 130.49, 130.40, 130.36, 129.9, 129.8, 129.7, 129.5, 129.2, 128.9, 128.80, 128.76, 128.7, 128.5, 128.3, 128.1, 127.8, 127.4, 125.6, 122.3, 118.5, 116.7, 115.0. HRMS (ESI$^+$): calcd for $C_{49}H_{31}N_2O_3^+$, 695.2329; found 695.2326.

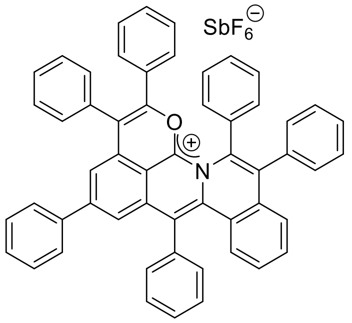

2,3,5,7,12,13-Hexaphenylisoquinolino[2,1-*b*]pyrano[4,3,2-*ij*]isoquinolin-1-ium hexafluoroantimonate (3na)

Following the general procedure. [1,1'-Biphenyl]-4-carbonitrile (35.8 mg, 0.2 mmol) and diphenylacetylene (53.5 mg, 0.3 mmol) were used. Purification via column chromatography on silica gel (dichloromethane/ethyl acetate = 10:1, v/v, then dichloromethane/methanol = 20:1, v/v) afforded **3na** as a yellow solid (53.8 mg, 56% yield). ^{1}H NMR (CDCl$_3$, 400 MHz): δ (ppm) 7.77 (s, 1H), 7.691-7.688 (m, 5H), 7.48-7.35 (m, 12H), 7.27-7.21 (m, 8H), 7.11-7.07 (m, 3H), 7.00 (d, J = 5.2 Hz, 2H), 6.84 (d, J = 7.2 Hz, 2H), 6.74-6.72 (m, 3H). ^{13}C NMR (CDCl$_3$, 100 MHz): δ (ppm) 155.5, 152.5, 151.6, 139.2, 138.2, 135.99, 135.94, 135.8, 134.2, 134.1, 134.0, 133.3, 131.6, 131.4, 131.1, 130.9, 130.6, 130.5, 130.1, 130.0, 129.8, 129.7, 129.6, 129.5, 129.43, 129.39, 129.1, 128.8, 128.6, 128.5, 128.4, 128.2, 128.1, 128.0, 127.7, 127.4, 127.3, 126.0, 122.4, 122.1, 121.4, 113.9. HRMS (ESI$^+$): calcd for $C_{55}H_{36}NO^+$, 726.2791; found 726.2797.

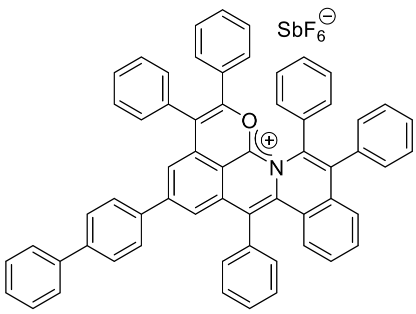

5-([1,1'-Biphenyl]-4-yl)-2,3,7,12,13-pentaphenylisoquinolino[2,1-*b*]pyrano[4,3,2-*ij*]isoquinolin-1-ium hexafluoroantimonate (3oa)

Following the general procedure. [1,1':4',1"-Terphenyl]-4-carbonitrile (51.1 mg, 0.2 mmol) and diphenylacetylene (53.5 mg, 0.3 mmol) were used. Purification via column chromatography on silica gel (dichloromethane/ethyl acetate = 10:1, v/v, then dichloromethane/methanol = 20:1, v/v) afforded **3oa** as a yellow solid (73.9 mg, 71% yield). ^{1}H NMR ($CDCl_3$, 400 MHz): δ (ppm) 7.813-7.810 (m, 1H), 7.72-7.71 (m, 4H), 7.66-7.64 (m, 2H), 7.59-7.57 (m, 2H), 7.54-7.53 (m, 3H), 7.47-7.41 (m, 4H), 7.39-7.33 (m, 6H), 7.30-7.27 (m, 5H), 7.24-7.21 (m, 3H), 7.15-7.08 (m, 3H), 7.03-7.00 (m, 2H), 6.87-6.85 (m, 2H), 6.77-6.71 (m, 3H). ^{13}C NMR ($CDCl_3$, 100 MHz): δ (ppm) 155.5, 152.6, 151.1, 142.7, 140.1, 138.3, 138.1, 136.2, 136.0, 135.9, 134.4, 134.2, 134.0, 133.3, 131.7, 131.6, 131.2, 130.9, 130.8, 130.7, 130.6, 130.1, 130.0, 129.9, 129.7, 129.54, 129.52, 129.2, 129.1, 128.9, 128.64, 128.57, 128.4, 128.12, 128.10, 128.0, 127.8, 127.4, 127.3, 127.2, 126.1, 122.21, 122.20, 121.2, 114.1. HRMS (ESI$^+$): calcd for $C_{61}H_{40}NO^+$, 802.3104; found 802.3098.

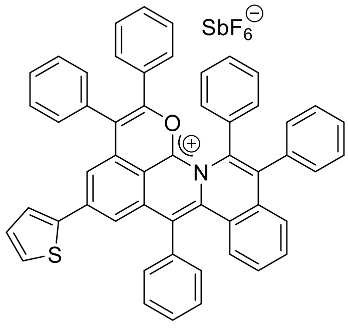

2,3,7,12,13-Pentaphenyl-5-(thiophen-2-yl)isoquinolino[2,1-*b*]pyrano[4,3,2-*ij*]isoquinolin-1-ium hexafluoroantimonate (3pa)

Following the general procedure **A**. 4-(Thiophen-2-yl)benzonitrile (37.1 mg, 0.2

mmol) and diphenylacetylene (53.5 mg, 0.3 mmol) were used. Purification via column chromatography on silica gel (dichloromethane/ethyl acetate = 10:1, v/v, then dichloromethane/methanol = 20:1, v/v) afforded **3pa** as a yellow solid (75.2 mg, 78% yield). ^{1}H NMR ($CDCl_3$, 400 MHz): δ (ppm) 7.74-7.66 (m, 5H), 7.46-7.37 (m, 7H), 7.35-7.28 (m, 5H), 7.25-7.21 (m, 5H), 7.14-7.07 (m, 5H), 6.99 (d, J = 6.8 Hz, 2H), 6.84 (d, J = 7.2 Hz, 2H), 6.76-6.70 (m, 3H). ^{13}C NMR ($CDCl_3$, 100 MHz): δ (ppm) 155.0, 152.6, 144.1, 142.0, 138.5, 136.2, 136.0, 135.9, 134.3, 134.2, 133.9, 133.2, 131.7, 131.4, 131.2, 130.9, 130.8, 130.7, 130.5, 130.12, 130.06, 129.8, 129.7, 129.6, 129.5, 129.23, 129.20, 128.8, 128.6, 128.5, 128.4, 128.1, 128.0, 127.8, 127.3, 127.0, 125.9, 121.8, 120.0, 119.2, 113.7. HRMS (ESI$^+$): calcd for $C_{53}H_{34}NOS^+$, 732.2356; found 732.2358.

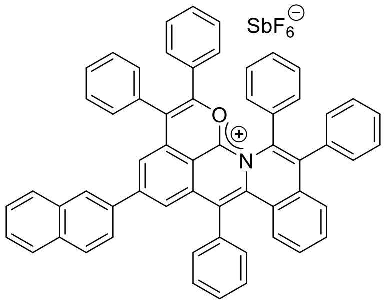

5-(Naphthalen-2-yl)-2,3,7,12,13-pentaphenylisoquinolino[2,1-*b*]pyrano[4,3,2-*ij*]isoquinolin-1-ium hexafluoroantimonate (3qa)

Following the general procedure. 4-(Naphthalen-2-yl)benzonitrile (45.9 mg, 0.2 mmol) and diphenylacetylene (53.5 mg, 0.3 mmol) were used. Purification via column chromatography on silica gel (dichloromethane/ethyl acetate = 10:1, v/v, then dichloromethane/methanol = 20:1, v/v) afforded **3qa** as a yellow solid (62.1 mg, 61% yield). ^{1}H NMR ($CDCl_3$, 400 MHz): δ (ppm) 7.91-7.82 (m, 5H), 7.74-7.66 (m, 4H), 7.6-7.597 (m, 1H), 7.55-7.50 (m, 3H), 7.45 (d, J = 8.4 Hz, 1H), 7.41-7.34 (m, 5H), 7.30-7.27 (m, 5H), 7.24-7.21 (m, 3H), 7.15-7.09 (m, 4H), 7.03-7.01 (m, 2H), 6.87 (d, J = 7.6 Hz, 2H), 6.77-6.71 (m, 3H). ^{13}C NMR ($CDCl_3$, 100 MHz): δ (ppm) 155.6, 152.6, 151.6, 138.3, 136.6, 136.1, 136.0, 135.8, 134.3, 134.2, 134.0, 133.6, 133.4, 133.3, 131.7, 131.5, 131.2, 130.9, 130.7, 130.6, 130.1, 130.0, 129.9, 129.7, 129.5, 129.3, 129.2, 128.9, 128.8, 128.6, 128.5, 128.4, 128.1, 128.08, 128.05, 127.8, 127.77,

127.5, 127.4, 127.3, 127.0, 126.1, 125.3, 122.7, 122.2, 121.6, 114.0. HRMS (ESI$^+$): calcd for $C_{59}H_{38}NO^+$, 776.2948; found 776.2941.

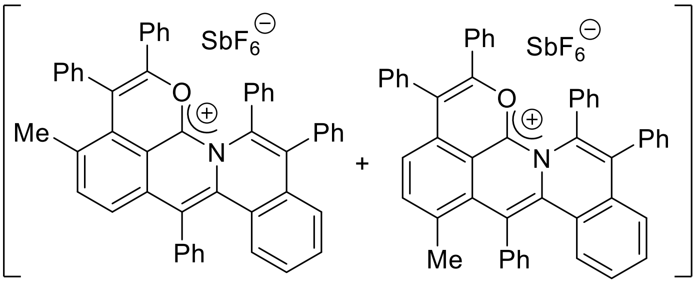

3ra-1 + **3ra-2**, 45% (8.3: 1)

4-Methyl-2,3,7,12,13-pentaphenylisoquinolino[2,1-*b*]pyrano[4,3,2-*ij*]isoquinolin-1-ium hexafluoroantimonate (3ra-1) and its isomer **(3ra-2)**

Following the general procedure. 3-Methylbenzonitrile (23.4 mg, 0.2 mmol) and diphenylacetylene (53.5 mg, 0.3 mmol) were used. Purification via column chromatography on silica gel (dichloromethane/ethyl acetate = 10:1, v/v, then dichloromethane/methanol = 20:1, v/v) afforded **3ra-1** and its isomer **3ra-2** (8.3: 1 from ^{1}H NMR spectrum) as a yellow solid (40.6 mg, 45% yield). HRMS (ESI$^+$): calcd for $C_{50}H_{34}NO^+$, 664.2635; found 664.2636.

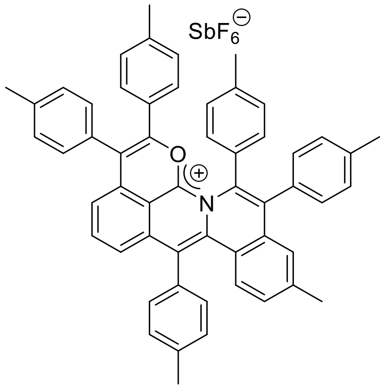

10-Methyl-2,3,7,12,13-penta-p-tolylisoquinolino[2,1-*b*]pyrano[4,3,2-*ij*]isoquinolin-1-ium hexafluoroantimonate (3ab)

Following the general procedure. Benzonitrile (20.6 mg, 0.2 mmol) and 1,2-di-p-tolylethyne (61.9 mg, 0.3 mmol) were used. Purification via column chromatography on silica gel (dichloromethane/ethyl acetate = 10:1, v/v, then dichloromethane/methanol = 20:1, v/v) afforded **3ab** as an orange solid (38.6 mg, 40% yield). ^{1}H NMR ($CDCl_3$, 400 MHz): δ (ppm) 7.97 (t, J = 8.0 Hz, 1H), 7.63 (d, J = 8.4

Hz, 1H), 7.48 (m, 3H), 7.37 (d, J = 8.8 Hz, 1H), 7.30 (d, J = 7.6 Hz, 1H), 7.18 (d, J = 7.6 Hz, 2H), 7.12-7.08 (m, 7H), 6.98-6.92 (m, 4H), 6.79 (d, J = 7.6 Hz, 2H), 6.71 (d, J = 8.0 Hz, 2H), 6.50 (d, J = 7.6 Hz, 2H), 2.55 (s, 3H), 2.35-2.33 (m, 6H), 2.29-2.27 (m, 6H), 1.94 (s, 3H). ^{13}C NMR ($CDCl_3$, 100 MHz): δ (ppm) 155.6, 152.4, 141.6, 140.2, 140.1, 139.0, 138.4, 138.2, 137.9, 137.8, 135.9, 135.4, 134.1, 133.4, 133.1, 131.8, 131.5, 131.4, 131.3, 130.9, 130.5, 130.3, 130.2, 129.9, 129.6, 129.2, 129.1, 128.8, 128.6, 128.4, 127.4, 127.1, 126.6, 123.9, 123.7, 122.0, 121.6, 114.5, 21.70, 21.68, 21.49, 21.48, 21.44, 21.3. HRMS (ESI$^+$): calcd for $C_{55}H_{44}NO^+$, 734.3417; found 734.3418.

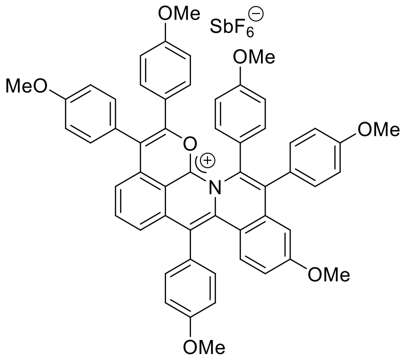

10-Methoxy-2,3,7,12,13-pentakis(4-methoxyphenyl)isoquinolino[2,1-*b*]pyrano[4,3,2-*ij*]isoquinolin-1-ium hexafluoroantimonate (3ac)

Following the general procedure. Benzonitrile (20.6 mg, 0.2 mmol) and 1,2-bis(4-methoxyphenyl)ethyne (71.5 mg, 0.3 mmol) were used. Purification via column chromatography on silica gel (dichloromethane/ethyl acetate = 10:1, v/v, then dichloromethane/methanol = 20:1, v/v) afforded **3ac** as a red solid (50.7 mg, 48% yield). ^{1}H NMR ($CDCl_3$, 400 MHz): δ (ppm) 7.96 (t, J = 8.0 Hz, 1H), 7.62 (d, J = 8.4 Hz, 1H), 7.50 (d, J = 8.4 Hz, 2H), 7.41 (d, J = 9.6 Hz, 1H), 7.29 (d, J = 7.6 Hz, 1H), 7.20 (d, J = 8.4 Hz, 2H), 7.14-7.11 (m, 3H), 6.92-6.81 (m, 9H), 6.74-6.71 (m, 2H), 6.66 (d, J = 8.4 Hz, 2H), 6.26 (d, J = 8.8 Hz, 2H), 3.96 (s, 3H), 3.81-3.78 (m, 9H), 3.70 (s, 3H), 3.47 (s, 3H). ^{13}C NMR ($CDCl_3$, 100 MHz): δ (ppm) 161.1, 160.8, 160.7, 159.9, 159.3, 159.2, 155.5, 152.3, 138.1, 138.0, 136.2, 135.6, 134.2, 134.0, 133.6, 132.4, 131.7, 131.3, 131.0, 130.1, 128.0, 126.38, 126.36, 125.1, 123.6, 123.4, 122.3, 121.5, 120.7, 119.2, 116.2, 115.7, 115.0, 114.04, 113.99, 113.5, 113.3, 111.0, 110.1,

55.7, 55.6, 55.5, 55.44, 55.35, 55.0. HRMS (ESI$^+$): calcd for $C_{55}H_{44}NO_7^+$, 830.3112; found 830.3112.

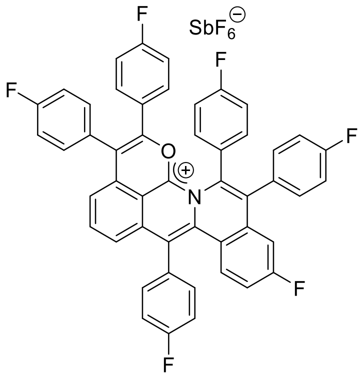

10-Fluoro-2,3,7,12,13-pentakis(4-fluorophenyl)isoquinolino[2,1-*b*]pyrano[4,3,2-*ij*] isoquinolin-1-ium hexafluoroantimonate (3ad)

Following the general procedure. Benzonitrile (20.6 mg, 0.2 mmol) and 1, 2-bis(4-fluorophenyl)ethyne (64.3 mg, 0.3 mmol) were used. Purification via column chromatography on silica gel (dichloromethane/ethyl acetate = 10:1, v/v, then dichloromethane/methanol = 20:1, v/v) afforded **3ad** as a yellow solid (80.2 mg, 81% yield). ^{1}H NMR ($CDCl_3$, 400 MHz): δ (ppm) 8.02 (t, J = 8.0 Hz, 1H), 7.64-7.59 (m, 3H), 7.46-7.42 (m, 1H), 7.37 (t, J = 8.4 Hz, 2H), 7.30 (d, J = 7.6 Hz, 1H), 7.22-7.16 (m, 4H), 7.06 (t, J = 8.4 Hz, 2H), 7.02-6.93 (m, 7H), 6.90-6.85 (m, 3H), 6.42 (t, J = 8.2 Hz, 2H). ^{13}C NMR ($CDCl_3$, 100 MHz): δ (ppm) 163.7 (d, *J* = 250.2 Hz), 163.6 (d, *J* = 251.4 Hz), 163.3 (d, *J* = 254.0 Hz), 163.0 (d, *J* = 248.5 Hz), 162.5 (d, *J* = 247.7 Hz), 162.2 (d, *J* = 249.3 Hz), 155.6, 151.4, 138.6, 137.96, 135.3, 135.1, 134.2 (d, *J* = 8.8 Hz), 133.8, 133.1 (d, *J* = 8.1 Hz), 132.9 (d, *J* = 2.6 Hz), 132.4 (d, *J* = 7.8 Hz), 132.3 (d, *J* = 7.8 Hz), 132.1 (d, *J* = 10.0 Hz), 132.0 (d, *J* = 8.9 Hz), 131.7 (d, *J* = 3.6 Hz), 131.0 (d, *J* = 8.4 Hz), 130.0 (d, *J* = 3.5 Hz), 129.5 (d, *J* = 3.5 Hz), 127.0 (d, *J* = 3.5 Hz), 126.1, 125.7 (d, *J* = 3.4 Hz), 124.0, 122.3, 122.26, 121.6, 118.2 (d, *J* = 21.5 Hz), 116.80 (d, *J* = 21.5 Hz), 116.78 (d, *J* = 22.0 Hz), 116.0 (d, *J* = 21.6 Hz), 115.4 (d, *J* = 21.9 Hz), 115.3 (d, *J* = 21.9 Hz), 114.8, 113.2 (d, *J* = 23.7 Hz). ^{19}F NMR ($CDCl_3$, 400 MHz): δ (ppm) -105.48, -108.20, -109.70, -110.59, -111.01, -111.98, -154.22, -154.28. HRMS (ESI$^+$): calcd for $C_{49}H_{26}F_6NO^+$, 758.1913; found 758.1915.

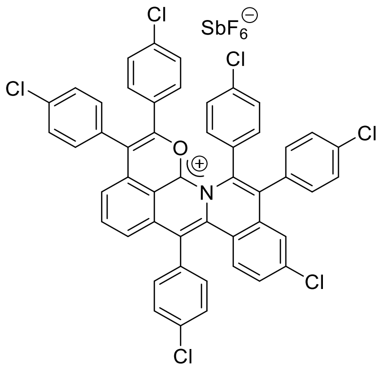

10-Chloro-2,3,7,12,13-pentakis(4-chlorophenyl)isoquinolino[2,1-*b*]pyrano[4,3,2-*ij*]isoquinolin-1-ium hexafluoroantimonate (3ae)

Following the general procedure. Benzonitrile (20.6 mg, 0.2 mmol) and 1, 2-bis(4-chlorophenyl)ethyne (74.1 mg, 0.3 mmol) were used. Purification via column chromatography on silica gel (dichloromethane/ethyl acetate = 10:1, v/v, then dichloromethane/methanol = 20:1, v/v) afforded **3ae** as a yellow solid (49.2 mg, 45% yield). ^{1}H NMR (CDCl$_3$, 400 MHz): δ (ppm) 8.01 (t, J = 7.8 Hz, 1H), 7.65-7.55 (m, 5H), 7.39-7.28 (m, 6H), 7.19-7.11 (m, 8H), 6.91 (d, J = 7.6 Hz, 2H), 6.85 (d, J = 7.6 Hz, 2H), 6.69 (d, J = 7.6 Hz, 2H). ^{13}C NMR (CDCl$_3$, 100 MHz): δ (ppm) 155.6, 151.3, 138.8, 137.8, 137.7, 136.8, 136.5, 135.6, 135.04, 135.00, 134.94, 134.86, 134.1, 133.5, 132.7, 132.6, 132.5, 132.3, 131.8, 131.7, 131.3, 131.0, 130.8, 130.2, 130.0, 129.3, 129.24, 129.22, 128.7, 128.6, 127.8, 126.63, 126.60, 124.3, 124.2, 122.564, 122.556, 121.9, 115.0. HRMS (ESI$^+$): calcd for $C_{49}H_{26}Cl_6NO^+$, 854.0140; found 854.0141.

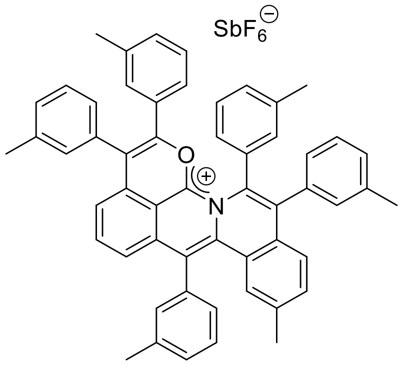

9-Methyl-2,3,7,12,13-penta-m-tolylisoquinolino[2,1-*b*]pyrano[4,3,2-*ij*]isoquinolin-1-ium hexafluoroantimonate (3af)

Following the general procedure. Benzonitrile (20.6 mg, 0.2 mmol) and 1, 2-di-*m*-tolylethyne (61.9 mg, 0.3 mmol) were used. Purification via column

chromatography on silica gel (dichloromethane/ethyl acetate = 10:1, v/v, then dichloromethane/methanol = 20:1, v/v) afforded **3af** as an orange solid (73.0 mg, 75% yield). ^{1}H NMR ($CDCl_3$, 400 MHz): δ (ppm) 8.01 (t, J = 7.8 Hz, 1H), 7.68-7.59 (m, 2H), 7.50-7.43 (m, 3H), 7.30 (d, J = 7.6 Hz, 1H), 7.24-7.15 (m, 6H), 7.08-6.95 (m, 7H), 6.78-6.72 (m, 4H), 6.61-6.56 (m, 2H), 2.49 (s, 3H), 2.30-2.21 (m, 9H), 2.05 (s, 3H), 1.73 (s, 3H). ^{13}C NMR ($CDCl_3$, 100 MHz): δ (ppm) 155.7, 152.3, 138.8, 138.2, 137.8, 137.6, 137.4, 136.1, 135.5, 135.2, 134.4, 134.3, 134.2, 132.4, 132.0, 131.5, 130.80, 130.75, 130.6, 130.4, 130.39, 130.38, 130.36, 130.1, 129.9, 129.83, 129.80, 129.4, 129.3, 129.0, 128.73, 128.71, 128.016, 128.005, 127.98, 127.6, 127.4, 127.3, 126.7, 125.8, 124.60, 124.58, 124.57, 124.55, 124.2, 122.3, 122.1, 114.8, 110.1, 21.8, 21.7, 21.5, 21.44, 21.36, 20.9. HRMS (ESI^{+}): calcd for $C_{55}H_{44}NO^{+}$, 734.3417; found 734.3418.

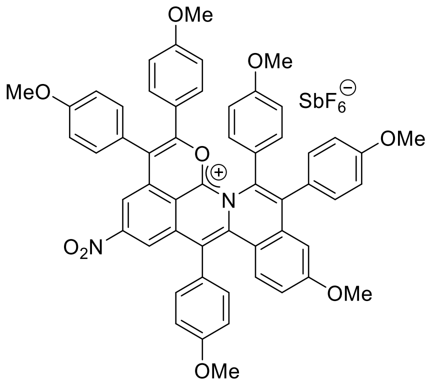

10-Methoxy-2,3,7,12,13-pentakis(4-methoxyphenyl)-5-nitroisoquinolino[2,1-*b*]pyrano[4,3,2-*ij*]isoquinolin-1-ium hexafluoroantimonate (3mc)

Following the general procedure. 4-Nitrobenzonitrile (29.6 mg, 0.2 mmol) and 1, 2-bis(4-methoxyphenyl)ethyne (71.5 mg, 0.3 mmol) were used. Purification via column chromatography on silica gel (dichloromethane/ethyl acetate = 10:1, v/v, then dichloromethane/methanol = 20:1, v/v) afforded **3mc** as a deep red solid (55.7 mg, 50% yield). ^{1}H NMR ($CDCl_3$, 400 MHz): δ (ppm) 8.32 (s, 1H), 7.84 (s, 1H), 7.54-7.48 (m, 3H), 7.23-7.20 (m, 2H), 7.15-7.11 (m, 4H), 6.92-6.84 (m, 9H), 6.78-6.76 (m, 1H), 6.65 (d, J = 8.4 Hz, 2H), 6.25 (d, J = 8.4 Hz, 2H), 3.98 (s, 3H), 3.82 (s, 3H), 3.79-3.77 (m, 6H), 3.71 (s, 3H), 3.46 (s, 3H). ^{13}C NMR ($CDCl_3$, 100 MHz): δ (ppm) 161.7,

161.076, 161.071, 160.2, 159.4, 159.3, 155.0, 154.1, 153.1, 139.0, 138.43, 138.37, 135.0, 134.6, 134.2, 132.3, 131.7, 131.5, 131.4, 130.1, 127.5, 126.6, 126.2, 123.0, 122.0, 120.9, 118.9, 117.7, 116.6, 116.2, 115.5, 115.3, 114.1, 113.8, 113.6, 113.4, 110.9, 55.7, 55.6, 55.5, 55.44, 55.36, 55.0. HRMS (ESI^+): calcd for $C_{55}H_{43}N_2O_9^+$, 875.2963; found 875.2962.

VII. Representative experiments on a 1.0 mmol scale

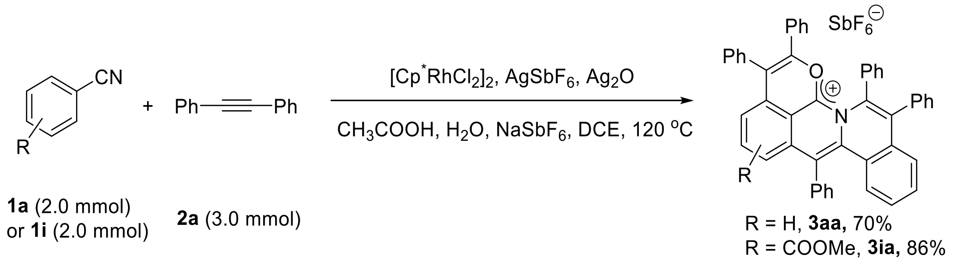

A Schlenk tube with a magnetic stir bar was charged with $AgSbF_6$ (69 mg, 200 µmol), $NaSbF_6$ (388.1 mg, 1.5 mmol), $[Cp^*RhCl_2]_2$ (30.9 mg, 50 µmol), Ag_2O (695.2 mg, 3.0 mmol), CH_3COOH (360.4 mg, 6.0 mmol), H_2O (144.2 µL, 8.0 mmol), benzonitrile or methyl 4-cyanobenzoate (2.0 mmol), diphenylacetylene (534.7 mg, 3.0 mmol), and DCE (5.0 mL) under an N_2 atmosphere. The resulting mixture was stirred at room temperature for 10 min and then at 120 ^oC for 12 h. Subsequently, it was diluted with 30 mL of dichloromethane. The mixture was evaporated under reduced pressure and the residue was absorbed to small amounts of silica gel. Purification was performed by column chromatography on silica gel to provide the desired product **3aa** (623.4 mg, 70% yield) or **3ia** (813.2 mg, 86%).

VIII. Mechanistic study

1. Control experiments

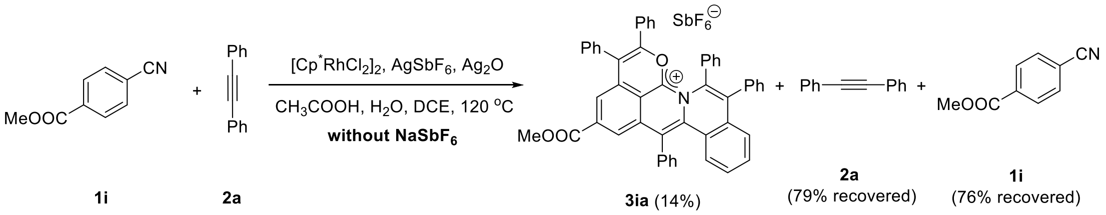

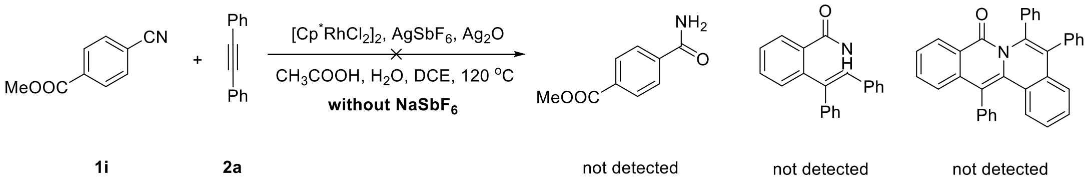

A Schlenk tube with a magnetic stir bar was charged with $AgSbF_6$ (6.9 mg, 20 μmol), $[Cp^*RhCl_2]_2$ (3.1 mg, 5.0 μmol), Ag_2O (69.5 mg, 0.30 mmol), CH_3COOH (36 μL, 0.6 mmol), H_2O (14.4 μL, 0.8 mmol), methyl 4-cyanobenzoate (32.2 mg, 0.2 mmol), diphenylacetylene (53.5 mg, 0.3 mmol), and DCE (0.5 mL) under an N_2 atmosphere. The resulting mixture was stirred at room temperature for 10 min and then at 120 ^oC for 12 h. The mixture was diluted with 10 mL of dichloromethane when it cooled down. The mixture was evaporated under reduced pressure and the residue was absorbed into small amounts of silica gel. Purification was performed by column chromatography on silica gel to provide **2a** (42.8 mg, 79% yield), **1i** (24.6 mg, 76% yield), **3ia** (13.5 mg, 14% yield). The proposed three compounds were not detected.

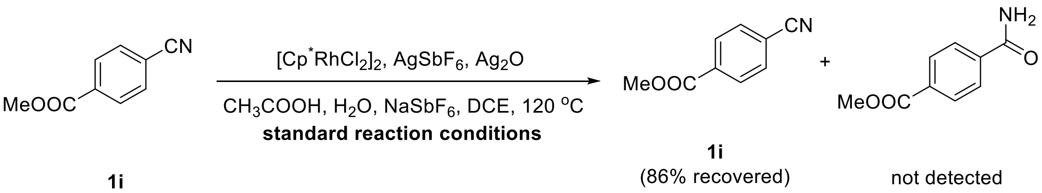

A Schlenk tube with a magnetic stir bar was charged with $AgSbF_6$ (6.9 mg, 20 μmol), $NaSbF_6$ (38.9 mg, 0.15 mmol), $[Cp^*RhCl_2]_2$ (3.1 mg, 5.0 μmol), Ag_2O (69.5 mg, 0.30 mmol), CH_3COOH (36.0 μL, 0.6 mmol), H_2O (14.4 μL, 0.8 mmol), methyl 4-cyanobenzoate (32.2 mg, 0.2 mmol), and DCE (0.5 mL) under an N_2 atmosphere. The resulting mixture was stirred at room temperature for 10 min and then at 120 ^oC for 12 h. The mixture was diluted with 10 mL of dichloromethane when it cooled down. The mixture was evaporated under reduced pressure and the residue was absorbed into small amounts of silica gel. Purification was performed by column

chromatography on silica gel to provide compound **1i** (27.7 mg, 86% yield recovered). The proposed amide was not detected.

2. The reaction of *ortho*-substituted arylnitriles with alkynes

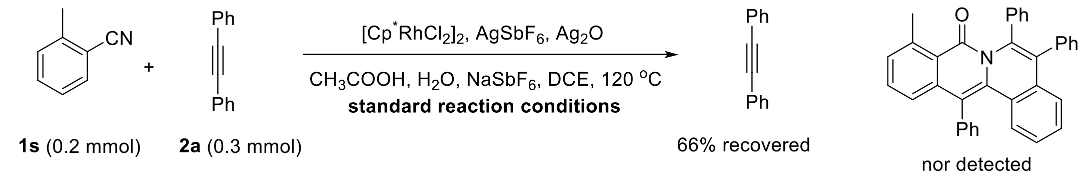

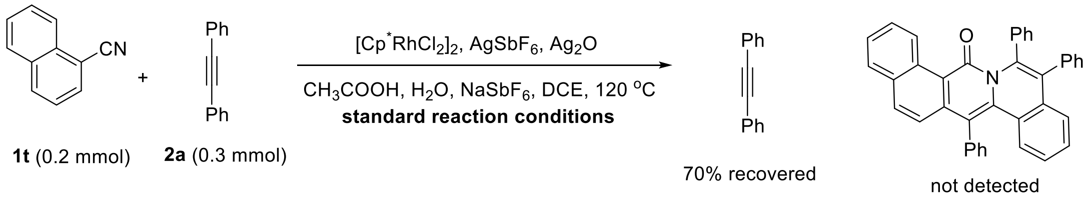

For **1s**: A Schlenk tube with a magnetic stir bar was charged with $AgSbF_6$ (6.9 mg, 20 μmol), $NaSbF_6$ (38.9 mg, 0.15 mmol), $[Cp^*RhCl_2]_2$ (3.1 mg, 5.0 μmol), Ag_2O (69.5 mg, 0.30 mmol), CH_3COOH (36.0 μL, 0.6 mmol), H_2O (14.4 μL, 0.8 mmol), 2-methylbenzonitrile (23.4 mg, 0.2 mmol), diphenylacetylene (53.5 mg, 0.3 mmol) and DCE (0.5 mL) under an N_2 atmosphere. The resulting mixture was stirred at room temperature for 10 min and then at 120 ^{o}C for 12 h. The mixture was diluted with 10 mL of dichloromethane when it cooled down. The mixture was evaporated under reduced pressure and the residue was absorbed into small amounts of silica gel. The purification was performed by column chromatography on silica gel to provide compound **1s** (35.4 mg, 66% yield recovered). The proposed annulation intermediate was not detected.

For **1t**: A Schlenk tube with a magnetic stir bar was charged with $AgSbF_6$ (6.9 mg, 20 μmol), $NaSbF_6$ (38.9 mg, 0.15 mmol), $[Cp^*RhCl_2]_2$ (3.1 mg, 5.0 μmol), Ag_2O (69.5 mg, 0.30 mmol), CH_3COOH (36.0 μL, 0.6 mmol), H_2O (14.4 μL, 0.8 mmol), 1-naphthonitrile (30.6 mg, 0.2 mmol), diphenylacetylene (53.5 mg, 0.3 mmol) and DCE (0.5 mL) under an N_2 atmosphere. The resulting mixture was stirred at room temperature for 10 min and then at 120 ^{o}C for 12 h. The mixture was diluted with 10 mL of dichloromethane when it cooled down. The mixture was evaporated under reduced pressure and the residue was absorbed into small amounts of silica gel.

Purification was performed by column chromatography on silica gel to provide compound **1s** (37.4 mg, 70% yield recovered). The proposed annulation intermediate was not detected.

3. Intermolecular competition experiment

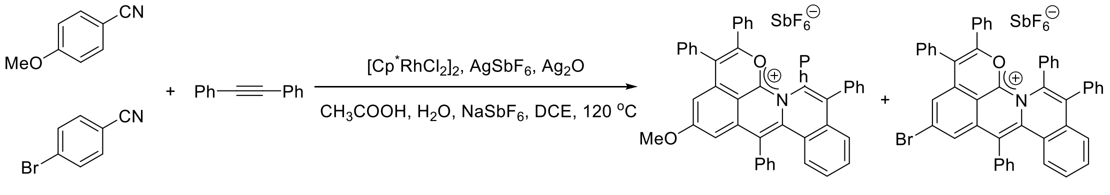

A Schlenk tube with a magnetic stir bar was charged with $AgSbF_6$ (6.9 mg, 20 μmol), $NaSbF_6$ (38.9 mg, 0.15 mmol), $[Cp^*RhCl_2]_2$ (3.1 mg, 5.0 μmol), Ag_2O (69.5 mg, 0.30 mmol), CH_3COOH (36 μL, 0.6 mmol), H_2O (14.4 μL, 0.8 mmol), 4-methoxybenzonitrile (26.6 mg, 0.2 mmol), 4-bromobenzonitrile (36.4 mg, 0.2 mmol), diphenylacetylene (53.5 mg, 0.3 mmol) and DCE (0.5 mL) under an N_2 atmosphere. The resulting mixture was stirred at room temperature for 10 min and then at 120 ^{o}C for 12 h. The mixture was diluted with 10 mL of dichloromethane when it cooled down. The mixture was evaporated under reduced pressure and the residue was absorbed into small amounts of silica gel. Purification was performed by column chromatography on silica gel to provide a mixture containing **3ca** and **3ga** 57.6 mg (**3ca: 3ga** ≈ 1: 3.3 according to the ^{1}H NMR spectrum).

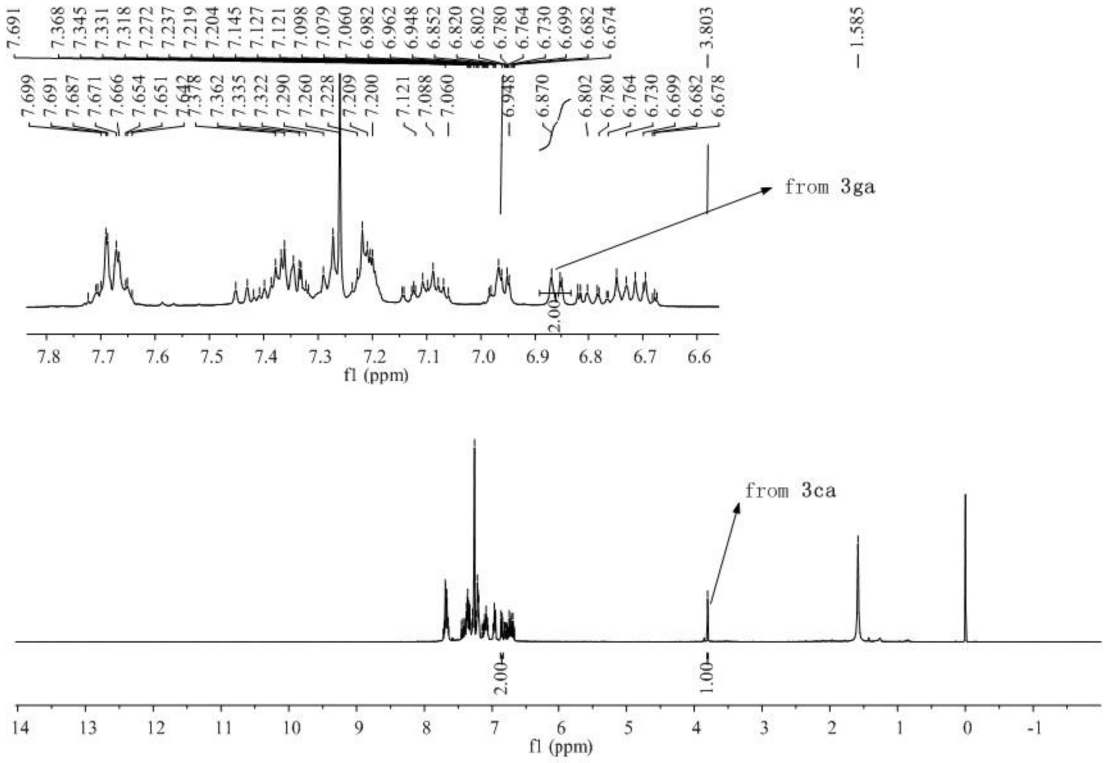

Figure S1. ^{1}H NMR spectrum of the mixture obtained from the intermolecular competition experiment.

4. Spectra of MAIDL-TOF-MS

General procedure for the preparation of the sample: A Schlenk tube with a magnetic stir bar was charged with $AgSbF_6$ (6.9 mg, 20 μmol), $NaSbF_6$ (38.9 mg, 0.15 mmol), $[Cp^*RhCl_2]_2$ (3.1 mg, 5.0 μmol), Ag_2O (69.5 mg, 0.30 mmol), CH_3COOH (36.0 μL, 0.6 mmol), H_2O (14.4 μL, 0.8 mmol), arylnitrile (0.20 mmol), diphenylacetylene (53.5 mg, 0.3 mmol) and DCE (0.5 mL) under an N_2 atmosphere. The resulting mixture was stirred at room temperature for 10 min and then at 120 ^oC for 1 h. The reaction mixture was used as the sample for the **MAIDL-TOF-MS** detection.

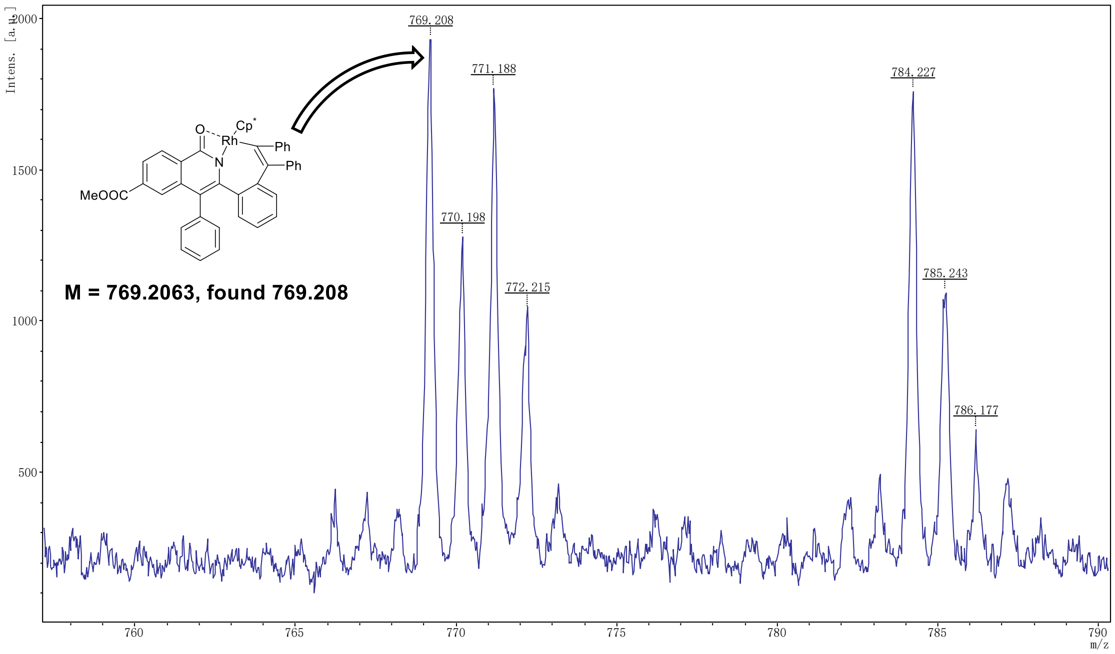

Figure S2. MAIDL-TOF-MS spectrum of the intermediate E.

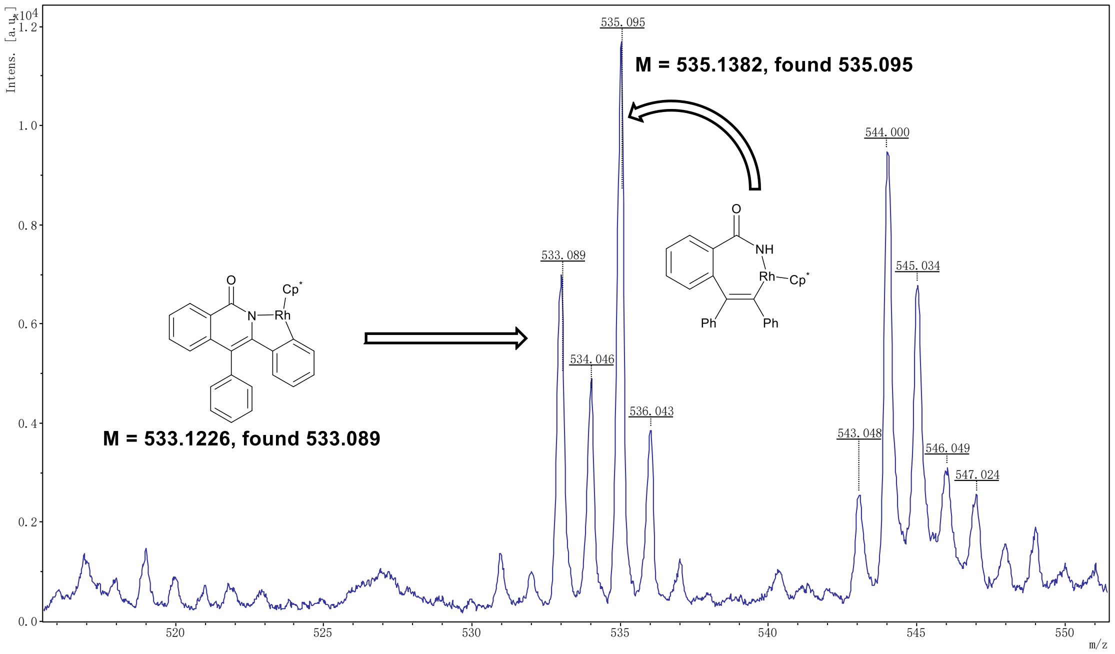

Figure S3. MAIDL-TOF-MS spectra of the intermediates C and D.

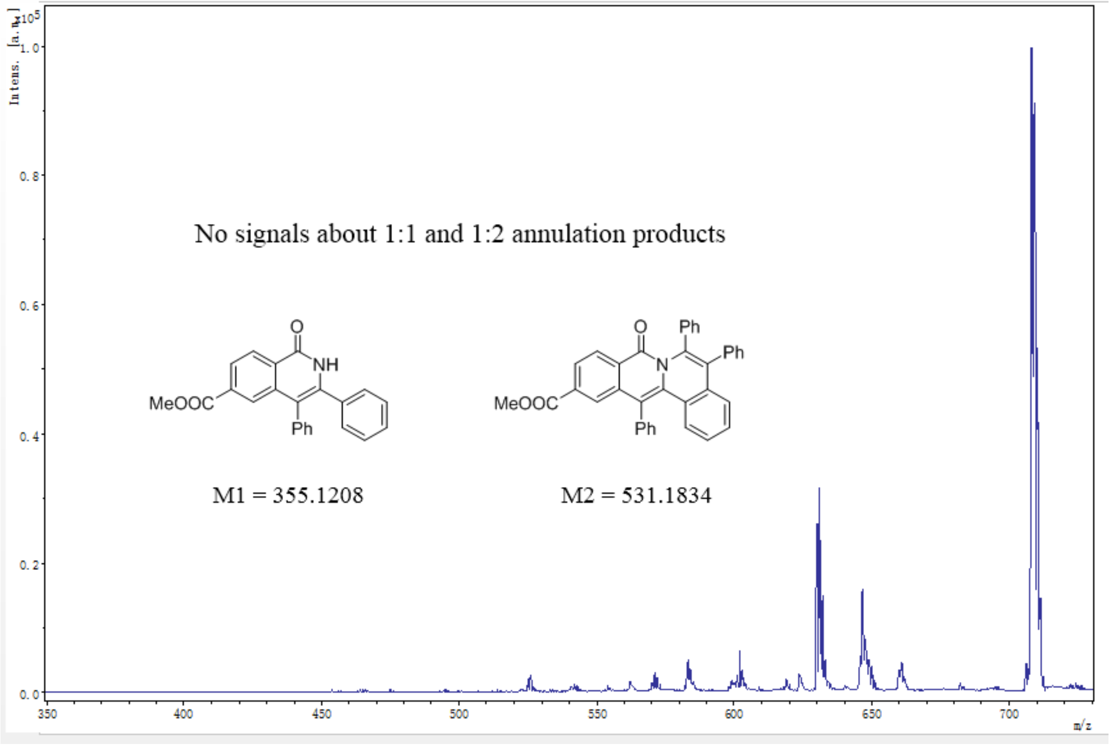

Figure S4. MAIDL-TOF-MS spectrum for the detection of the 1:1 and 1:2 annulation products.

IX. Photophysical properties of the representative products

Table S2. Photophysical data of the representative products[a]

Compound	λ_{abs}/nm	λ_{ex}/nm	λ_{em}/nm	Stokes shift (cm^{-1})	Φ_F
**3aa**	501	502	575	2569	0.11
**3ca**	468	472	549	3153	0.23
**3da**	476	478	561	3183	0.08
**3ha**	518	518	592	2413	0.14
**3ia**	534	535	599	2032	0.21
**3la**	548	548	608	1801	0.27
**3ma**	559	563	622	1812	0.23
**3ac**	492	492	604	3769	0.10
**3ae**	503	504	576	2520	0.11

**3mc**	587	595	674	2199	< 0.01

[a] Emission maximum in CH_2Cl_2 at 10.0 μM.

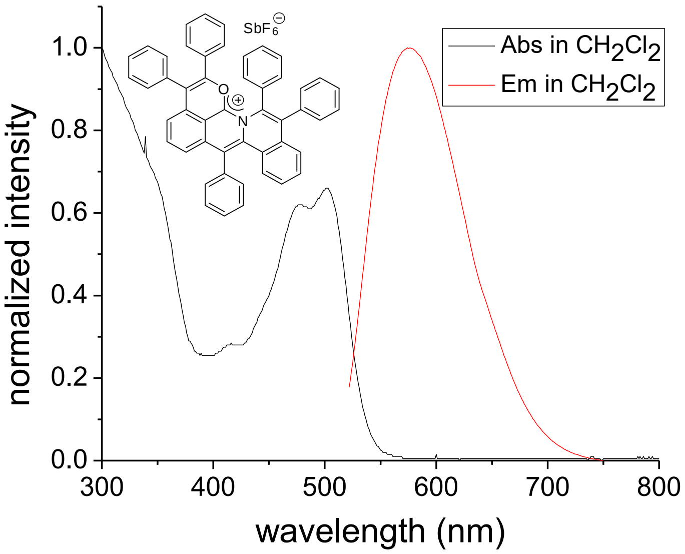

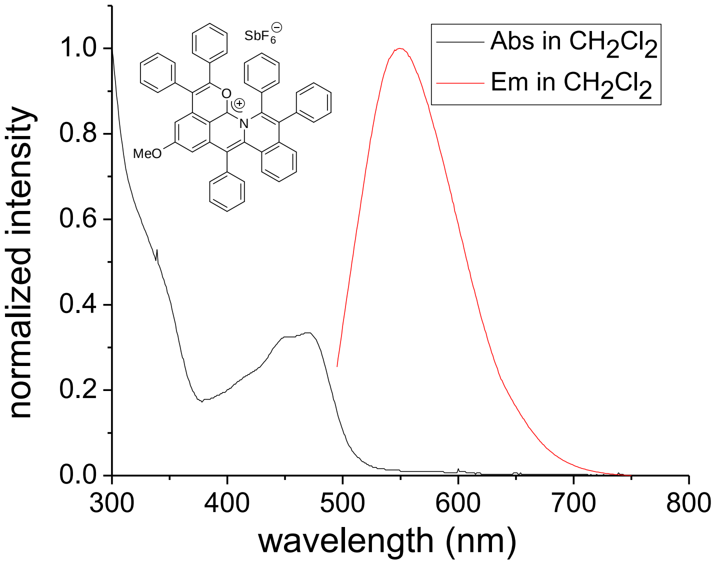

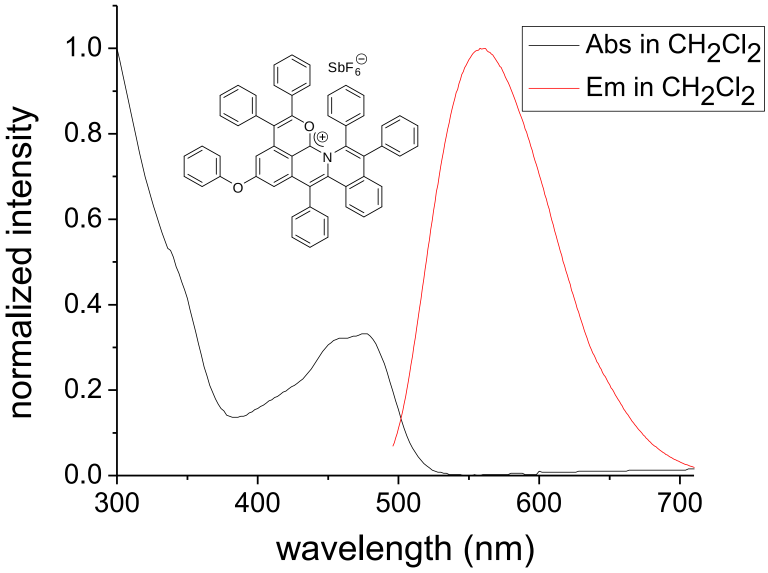
Abs in CH_2Cl_2
Em in CH_2Cl_2
normalized intensity
wavelength (nm)
$SbF_6^{\ominus}$
O
N

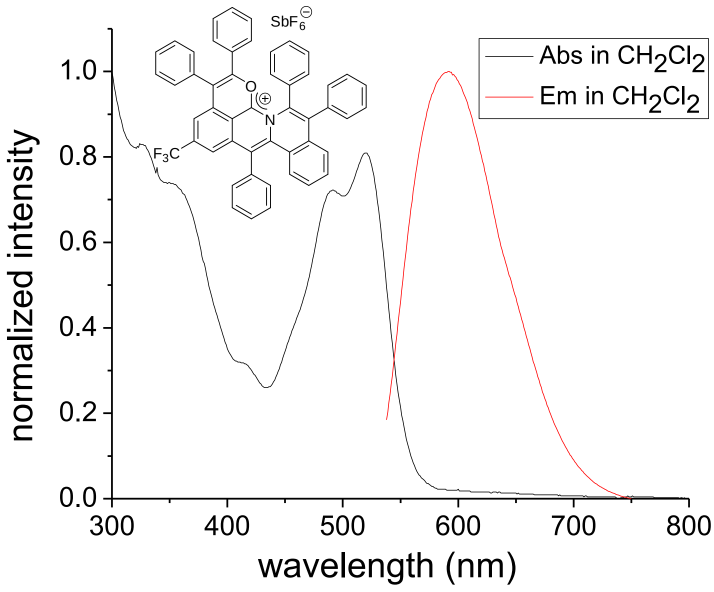
Abs in CH_2Cl_2
Em in CH_2Cl_2
normalized intensity
wavelength (nm)
$SbF_6^{\ominus}$
F_3C
O
N

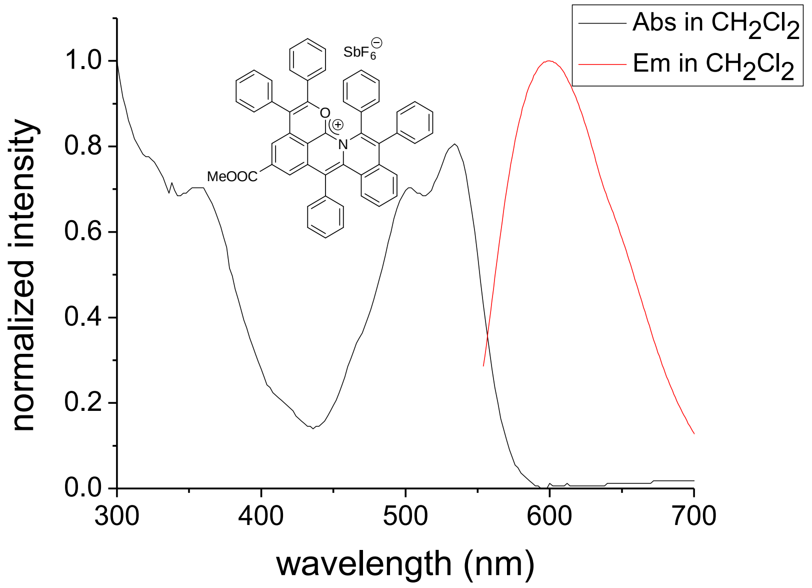
Abs in CH_2Cl_2
Em in CH_2Cl_2
normalized intensity
wavelength (nm)
MeOOC
O
N
$SbF_6^{\ominus}$
1.0
0.8
0.6
0.4
0.2
0.0
300
400
500
600
700

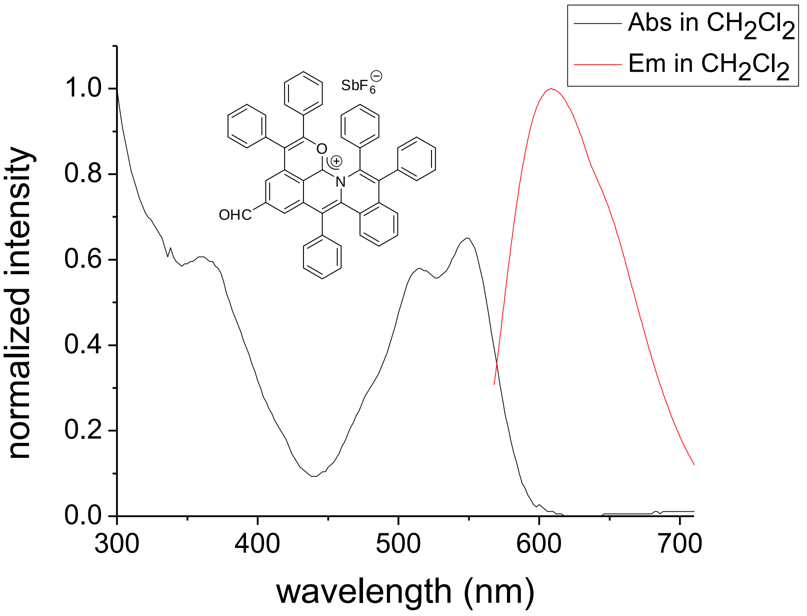
Abs in CH_2Cl_2
Em in CH_2Cl_2
normalized intensity
wavelength (nm)
OHC
O
N
$SbF_6^{\ominus}$
1.0
0.8
0.6
0.4
0.2
0.0
300
400
500
600
700

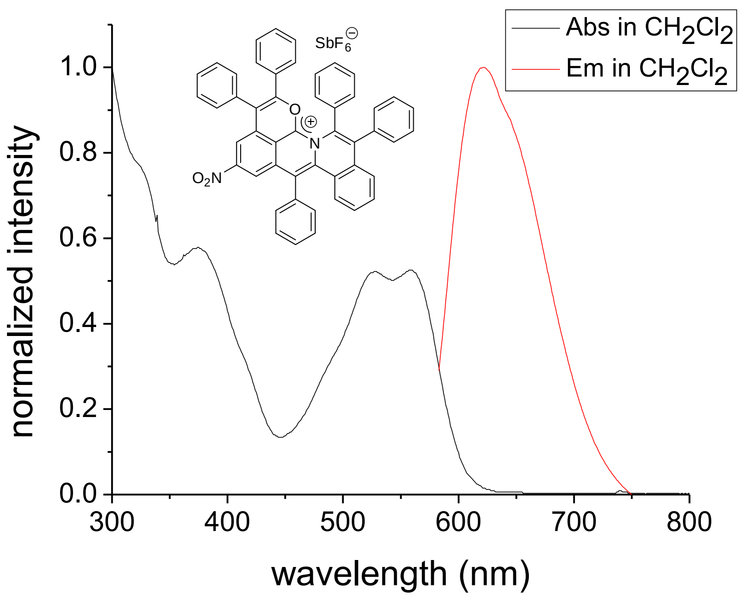

Abs in CH_2Cl_2
Em in CH_2Cl_2
normalized intensity
wavelength (nm)
$SbF_6^{\ominus}$
O
N
O_2N

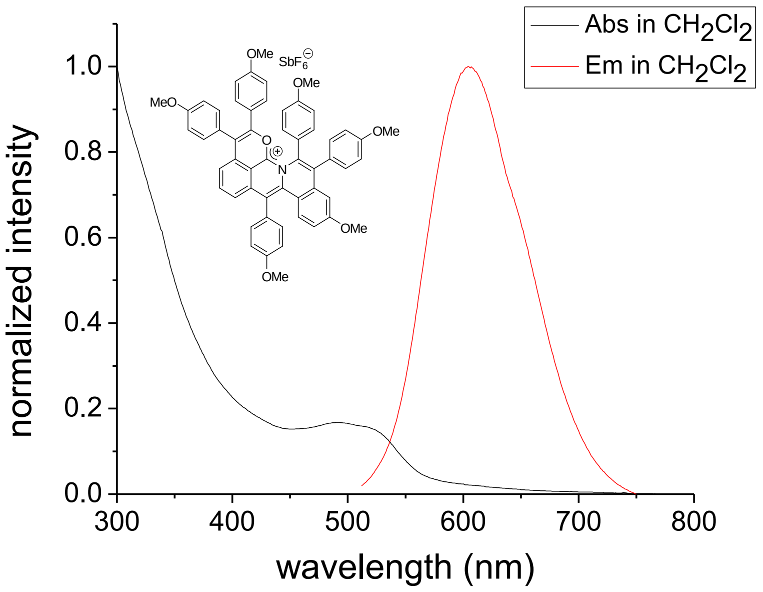

Abs in CH_2Cl_2
Em in CH_2Cl_2
normalized intensity
wavelength (nm)
$SbF_6^{\ominus}$
OMe
MeO
O
N

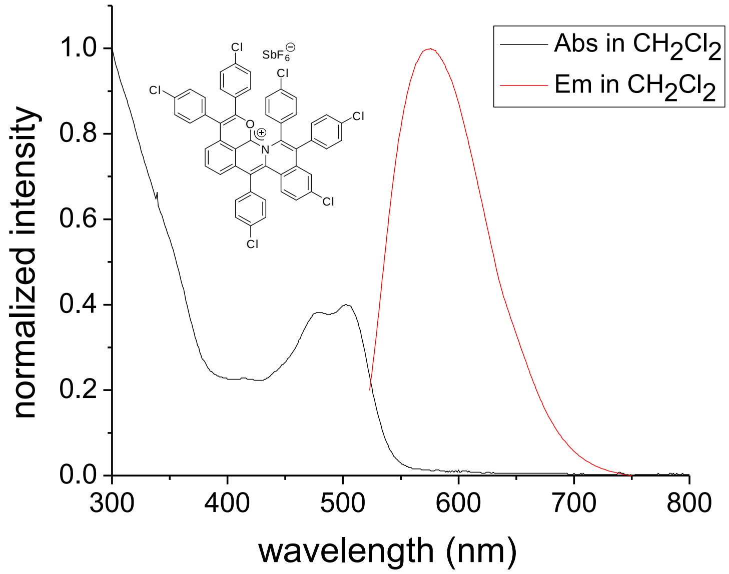

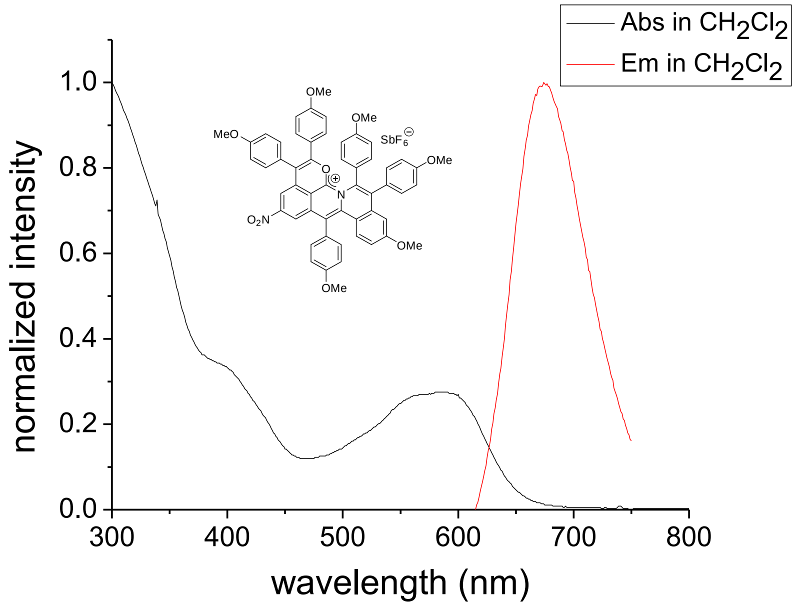

Figure S5. The absorption and emission spectra of the representative products.

X. Cytotoxicity assay and cell imaging experiment

1. Cell culture

The used HepG2 cells were incubated in DMEM (Dublecco's Minimum Eagle's Medium) in which 10% (v/v) FBS (fetal bovine serum), penicillin (100 units/mL) and streptomycin (100 mg/mL) was supplemented at 37 ^{o}C in a 5% CO_2 containing

humidified atmosphere.

2. Cytotoxicity assay

The cytotoxicity experiments of **3da** and **3ia** were investigated by CellTiler 96®AQueous One Solution Cell Proliferation Assay. HepG2 cells were seeded at 1×104 cells/well in 96-well culture plates for a stationary culture. After being incubated for 24 h, the medium was replaced with fresh complete medium, and the sample was then added to achieve final concentrations at 0, 0.625, 1.25, 2.5, 5.0, 10.0 and 20.0 μM. After 24 hours of incubation, 20 μL of CellTiler 96®AQueous One Solution in PBS was added to each well and the plates were incubated for additional one hour. Afterwards, the absorbance of each sample was recorded on the ELISA plate reader (model 680, BioRad) at a wavelength of 490 nm. The cell viability was calculated by the following formula: (mean optical density (OD) in treated wells/mean OD in control wells) ×100%.

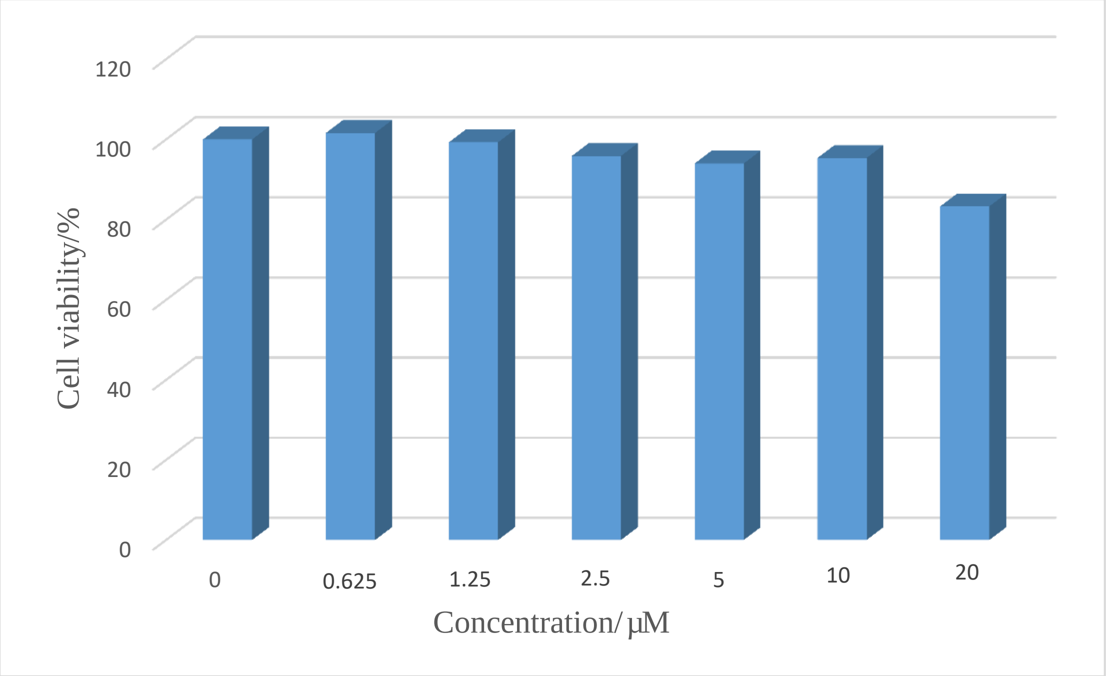

Figure S6. **Cell viability values (%) estimated by CellTiler 96®AQueous One Solution Cell Proliferation Assay employing HepG2 cells, stained with 0−20 μM of 3ia, at 37 ℃ for 24 h.**

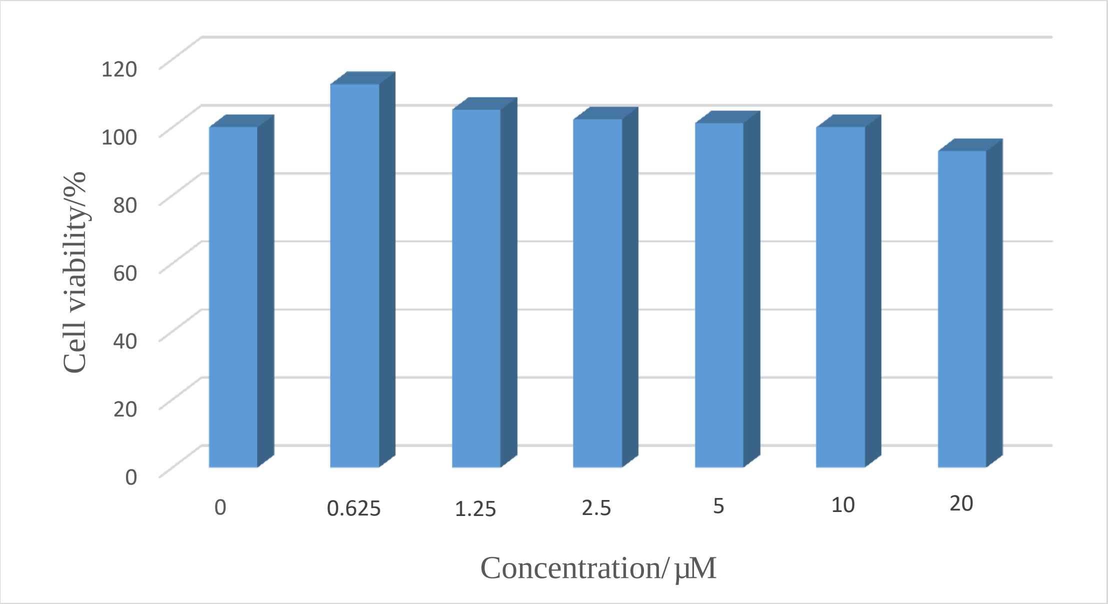

Figure S7. **Cell viability values (%) estimated by CellTiler 96®AQueous One Solution Cell Proliferation Assay employing HepG2 cells, stained with 0−20 μM of 3da, at 37 ℃ for 24 h.**

3. Confocal imaging experiment

For subcellular localization experiments, HepG2 cells were incubated with 1 μM **3da** or **3ia** in PBS (phosphate buffered solution) containing 1‰ DMSO for 15 min at 37 ℃. After incubation HepG2 cells were washed twice with PBS and 1.0 μM LysoTrackers were then added to incubate for 30 min, respectively. Finally, the cells were washed twice with PBS before imaging. The cells were observed with a LSM 780 (Zeiss) confocal laser scanning microscope.

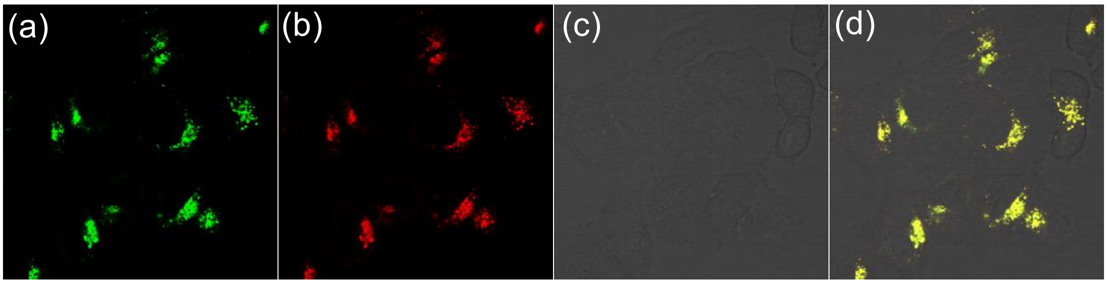

Figure S8. Co-staining of HepG2 cells with 3ia (1.0 μM) and LTG (LysoTracker® Green DND-26) (1.0 μM): a) fluorescent image of HepG2cells stained with LTG for 30 min (λ_{ex} = 488 nm, λ_{em} = 460−560 nm); b) fluorescent image of HepG2 cells stained with 3ia for 15 min

(λ_{ex} = 552 nm, λ_{em} = 550−650 nm); c) bright-field image; and d) merged images of (a), (b) and (c).

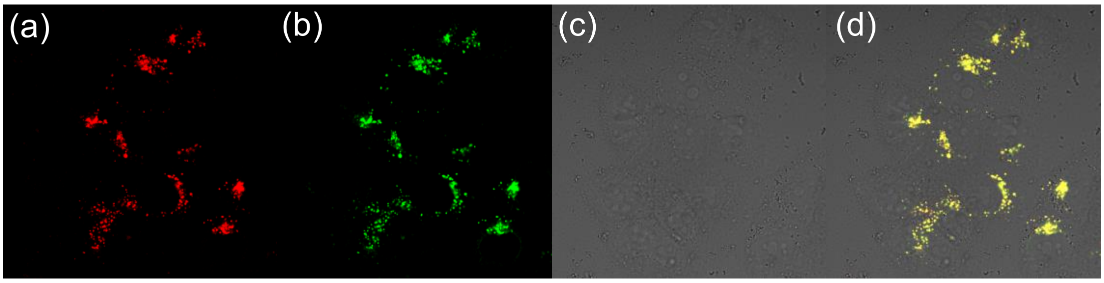

Figure S9. Co-staining of HepG2 cells with 3da (1.0 μM) and LTR (LysoTracker™ Red DND-99) (1.0 μM): a) fluorescent image of HepG2cells stained with LTR for 30 min (λ_{ex} = 546 nm, λ_{em} = 550−650 nm); b) fluorescent image of HepG2 cells stained with 3da for 15 min (λ_{ex} = 488 nm, λ_{em} = 500−600 nm); c) bright-field image; and d) merged images of (a), (b) and (c).

XI. Single crystal X-ray structures of 3ea and 3ia

Table S3. Crystal data and structure refinement for 3ea

Identification code	**3ea**
Empirical formula	$C_{50}H_{33}Cl_2F_7NOSb$
Formula weight	989.42
Temperature/K	293.15
Crystal system	trigonal
Space group	R-3
a/Å	28.6364(8)
b/Å	28.6364(8)
c/Å	29.7717(8)
α/°	90
β/°	90
γ/°	120
Volume/Å^3	21143.1(14)
Z	18
ρ_{calc}g/cm^3	1.399
μ/mm^{-1}	0.765
F(000)	8928.0
Crystal size/mm^3	0.4 × 0.35 × 0.3
Radiation	MoKα (λ = 0.71073)
2Θ range for data collection/°	6.08 to 52.744
Index ranges	$-35 \le h \le 21$, $-19 \le k \le 35$, $-23 \le l \le 37$
Reflections collected	19566
Independent reflections	9597 [R_{int} = 0.0228, R_{sigma} = 0.0499]
Data/restraints/parameters	9597/0/559
Goodness-of-fit on F^2	1.044
Final R indexes [I>=2σ (I)]	R_1 = 0.0613, wR_2 = 0.1712
Final R indexes [all data]	R_1 = 0.1073, wR_2 = 0.1921
Largest diff. peak/hole / e Å^{-3}	0.75/-0.71

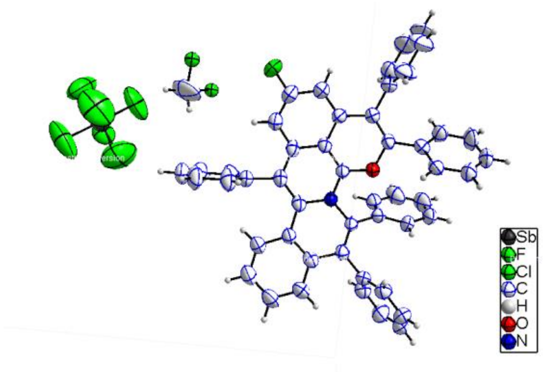

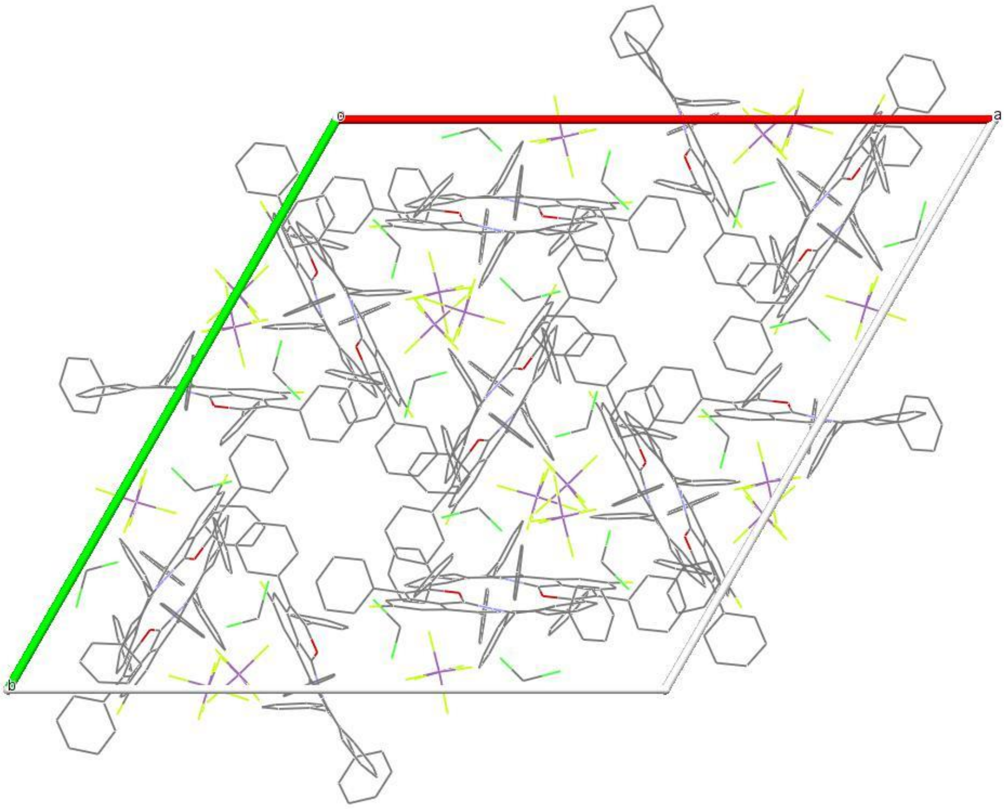

o
a
b

Table S4. Crystal data and structure refinement for 3ia.

Identification code	**3ia**
Empirical formula	$C_{51.5}H_{35}ClF_6NO_3Sb$
Formula weight	987.00
Temperature/K	296.7(8)
Crystal system	monoclinic
Space group	$P2_1/n$
a/Å	9.14367(15)
b/Å	44.3544(8)
c/Å	12.23171(19)
α/°	90
β/°	109.9481(17)
γ/°	90
Volume/Å^3	4663.08(14)
Z	4
ρ_{calc}g/cm^3	1.406
μ/mm^{-1}	5.787
F(000)	1988.0
Crystal size/mm^3	0.7 × 0.45 × 0.35
Radiation	CuKα (λ = 1.54184)
2Θ range for data collection/°	9.744 to 145.286
Index ranges	$-9 \leq h \leq 11$, $-54 \leq k \leq 53$, $-15 \leq l \leq 10$
Reflections collected	25902
Independent reflections	9073 [R_{int} = 0.0435, R_{sigma} = 0.0397]
Data/restraints/parameters	9073/0/587
Goodness-of-fit on F^2	1.069
Final R indexes [I>=2σ (I)]	R_1 = 0.0801, wR_2 = 0.2260
Final R indexes [all data]	R_1 = 0.0896, wR_2 = 0.2355
Largest diff. peak/hole / e Å^{-3}	2.19/-1.40

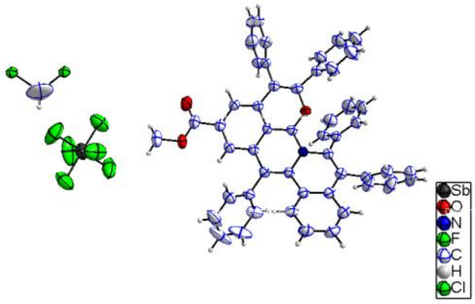

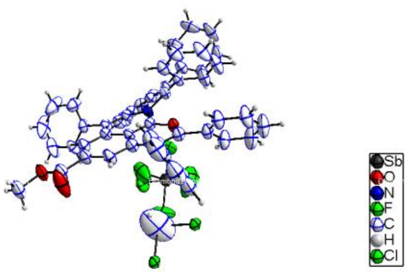

XII. DFT calculation[4, 5]

1. Complete Reference for Gaussian 09

Gaussian 09, Revision D.01, M. J. Frisch, G. W. Trucks, H. B. Schlegel, G. E. Scuseria, M. A. Robb, J. R. Cheeseman, G. Scalmani, V. Barone, B. Mennucci, G. A. Petersson, H. Nakatsuji, M. Caricato, X. Li, H. P. Hratchian, A. F. Izmaylov, J. Bloino, G. Zheng, J. L. Sonnenberg, M. Hada, M. Ehara, K. Toyota, R. Fukuda, J. Hasegawa, M. Ishida, T. Nakajima, Y. Honda, O. Kitao, H. Nakai, T. Vreven, J. A. Montgomery, Jr., J. E. Peralta, F. Ogliaro, M. Bearpark, J. J. Heyd, E. Brothers, K. N. Kudin, V. N. Staroverov, R. Kobayashi, J. Normand, K. Raghavachari, A. Rendell, J. C. Burant, S. S. Iyengar, J. Tomasi, M. Cossi, N. Rega, N. J. Millam, M. Klene, J. E. Knox, J. B. Cross, V. Bakken, C. Adamo, J. Jaramillo, R. Gomperts, R. E. Stratmann, O. Yazyev, A. J. Austin, R. Cammi, C. Pomelli, J. W. Ochterski, R. L. Martin, K. Morokuma, V. G. Zakrzewski, G. A. Voth, P. Salvador, J. J. Dannenberg, S. Dapprich, A. D. Daniels, Ö. Farkas, J. B. Foresman, J. V. Ortiz, J. Cioslowski, D. J. Fox, Gaussian, Inc., Wallingford CT, **2013**.

2. Computational Methods

The DFT calculations were carried out with the GAUSSIAN 09 series of programs. Density functional theory B3LYP[1] with a standard 6-31+G(d) basis set was used for geometry optimizations. Harmonic frequency calculations were performed for stationary point to confirm them as a local minima.

3. B3LYP Geometries for the Optimized Compounds and Transition State (3ia)

O	-0.27815800	-1.44429700	-0.55475400
C	-1.53074800	-2.06582900	-0.55306200
C	-0.13796500	-0.14729700	-0.31263000
O	-4.32345100	4.43851000	0.91510400
C	-4.61852400	3.14229000	0.72798500
C	-5.43928000	5.30403300	1.22750600
N	1.13859200	0.32054600	-0.22474500
C	1.36812200	1.71535900	-0.41357600
C	2.21787300	-0.56048500	0.14918400
C	0.32037900	2.59799500	-0.16923300

C	2.70089700	2.03122300	-0.94763600
C	-1.23710600	0.68844400	-0.13882600
C	-1.01006100	2.08442600	0.04790100
C	-2.54054300	0.10062400	-0.09598300
C	1.87102200	-1.68549600	1.06608900
C	3.48876800	-0.19944500	-0.17084000
C	-2.13026500	2.88177100	0.35846500
C	0.51445600	4.07816000	-0.10502600
C	1.25150800	-1.38765500	2.29307200
C	2.19670400	-3.01708800	0.77422500
C	-2.66015200	-1.33324800	-0.33069900
C	-1.37948300	-3.50254900	-0.84725100
C	-3.61787600	0.94435800	0.16684200
C	3.73636300	1.05788300	-0.86932300
C	2.96824500	3.23858500	-1.62870800
C	-0.10172500	4.93372800	-1.03445200
C	1.28971100	4.63670200	0.92364700
C	4.65482000	-1.03701600	0.25705300
C	-4.01773100	-1.95688400	-0.37963500
O	-5.73883400	2.67835500	0.80327700
C	-2.11319700	-4.47612700	-0.14760200
C	-0.46855000	-3.91287300	-1.83923800
H	-2.01188800	3.94088500	0.54234300
C	-3.40261800	2.31316900	0.41149200
C	-4.55972600	-2.37654700	-1.60366500
C	-4.77802900	-2.10177400	0.79215100
C	5.00359000	1.34672300	-1.41320100
C	5.23343000	-1.95752700	-0.63002000
C	5.20746500	-0.88050600	1.53621200
H	-4.63143800	0.56411800	0.20204100
H	2.19148200	3.97851500	-1.75031100
C	4.21662900	3.49038300	-2.18372600
H	1.01048300	-0.35650400	2.54368300
C	0.97175700	-2.40151600	3.21101700
H	-0.70593700	4.51501400	-1.83569500
C	0.07509100	6.31652000	-0.94993800
C	5.24581800	2.54807800	-2.06541000
H	6.22649600	2.74904000	-2.48687700
H	2.66526700	-3.26223700	-0.17287000
C	1.91612500	-4.02973300	1.69325100
H	4.81744300	-2.07976100	-1.62765500
C	6.33972700	-2.71692500	-0.23947800
H	5.79522200	0.61176200	-1.32345300
H	4.38518700	4.42309400	-2.71406300

H	1.76149200	3.98517300	1.65478500
C	1.45796800	6.02048100	1.01271900
H	-2.80590600	-4.17879700	0.63163900
C	-1.94356500	-5.82898400	-0.44195100
H	-3.98113400	-2.26212500	-2.51667600
C	-5.83630500	-2.94183700	-1.65339700
H	0.10355600	-3.17063800	-2.38805900
C	-0.31190800	-5.26592100	-2.13714600
H	4.77001100	-0.16543400	2.22832600
C	6.31176400	-1.64232000	1.92677200
H	-4.36943400	-1.77633400	1.74614200
C	-6.05304900	-2.66990500	0.74066800
H	2.17219000	-5.05766200	1.45247000
C	1.30433400	-3.72661600	2.91275300
H	-2.51162400	-6.57194400	0.11075300
C	-1.04804300	-6.22824500	-1.43868900
H	0.50719900	-2.15446900	4.16181100
H	-6.24549700	-3.26324700	-2.60719500
C	-6.58410800	-3.09181600	-0.48217100
H	-6.16724800	5.28389100	0.41317100
H	-5.00656500	6.29750600	1.34003200
H	-5.91701700	4.97833600	2.15443100
H	6.72749500	-1.51577900	2.92268700
C	6.87961600	-2.56232200	1.04077600
H	1.09052400	-4.51647100	3.62744200
H	-6.63096500	-2.77965000	1.65418200
H	6.77788100	-3.42786700	-0.93486700
H	-0.92481400	-7.28262400	-1.67012000
H	7.73917800	-3.15314800	1.34489000
H	-0.39775400	6.96533800	-1.68217500
C	0.85599800	6.86332200	0.07367600
H	0.38162900	-5.56872700	-2.91682100
H	-7.57726600	-3.53038700	-0.52198000
H	2.05863000	6.43888800	1.81564800
H	0.99077700	7.93923700	0.14086500

XIII. References

(1) K. W. Kang, K. Moseley, P. M. Maitlis, *J. Am. Chem. Soc.* **1969**, *91*, 5970.

(2) C. S. Sevov, J. Zhou, J. F. Hartwig, *J. Am. Chem. Soc.* **2014**, *136*, 3200.

(3) K. Park, G. Bae, J. Moon, J. Choe, K. H. Song, S. Lee, *J. Org. Chem.* **2010**, *75*,

6244.

(4) A. D. Becke, *J. Chem. Phys*. **1993**, *98*, 5648;

(5) C. Lee, W. Yang, R. G. Parr, *Phys. Rev. B* **1988**, *37*, 785.

XIV. Copies of NMR spectra

^{1}H NMR spectra of **3aa** ($CDCl_3$)

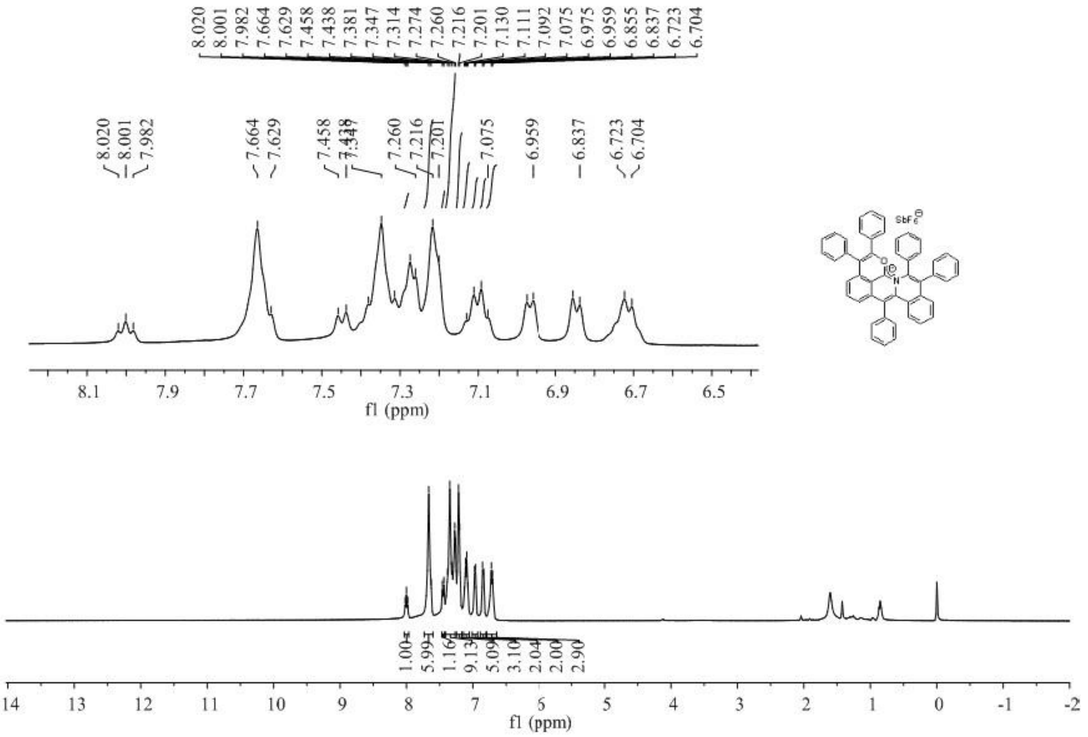

^{13}C NMR spectra of **3aa** ($CDCl_3$)

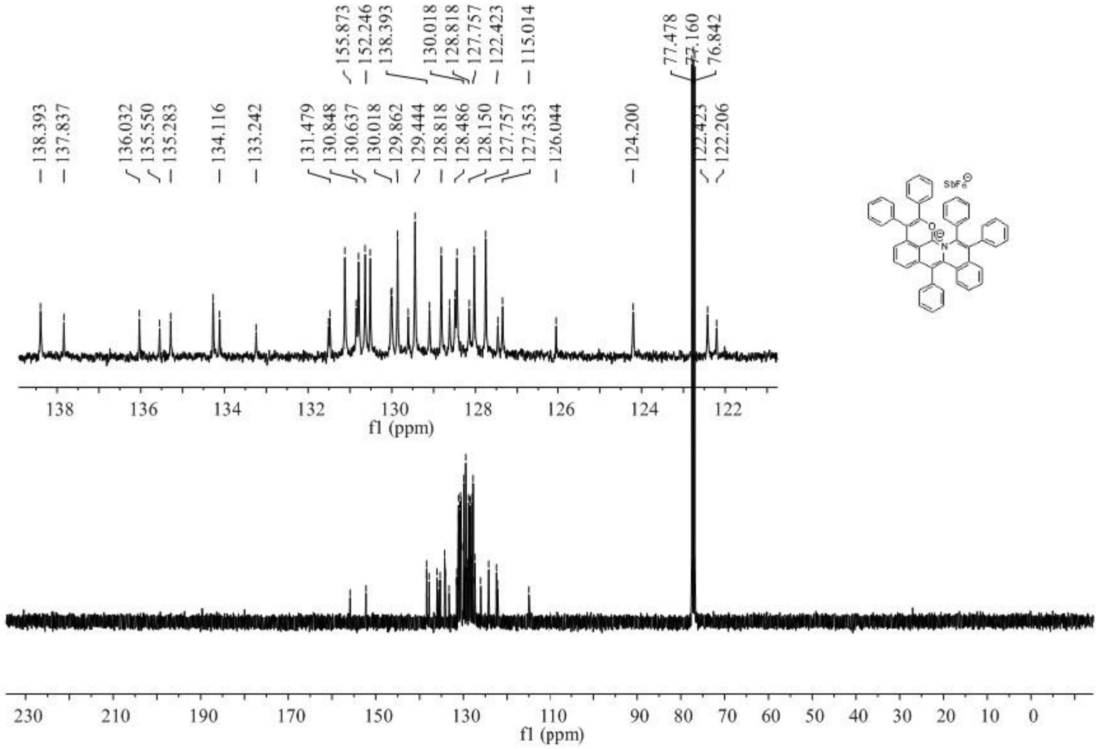

^{1}H NMR spectra of **3ba** ($CDCl_3$)

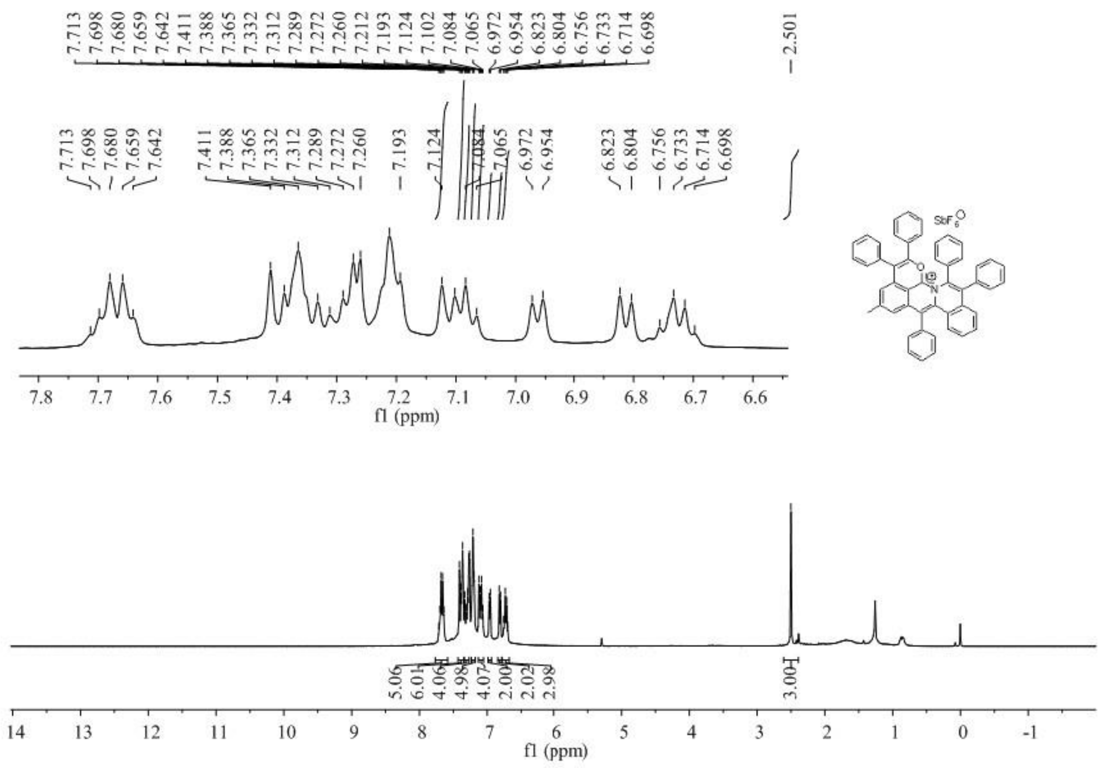

^{13}C NMR spectra of **3ba** ($CDCl_3$)

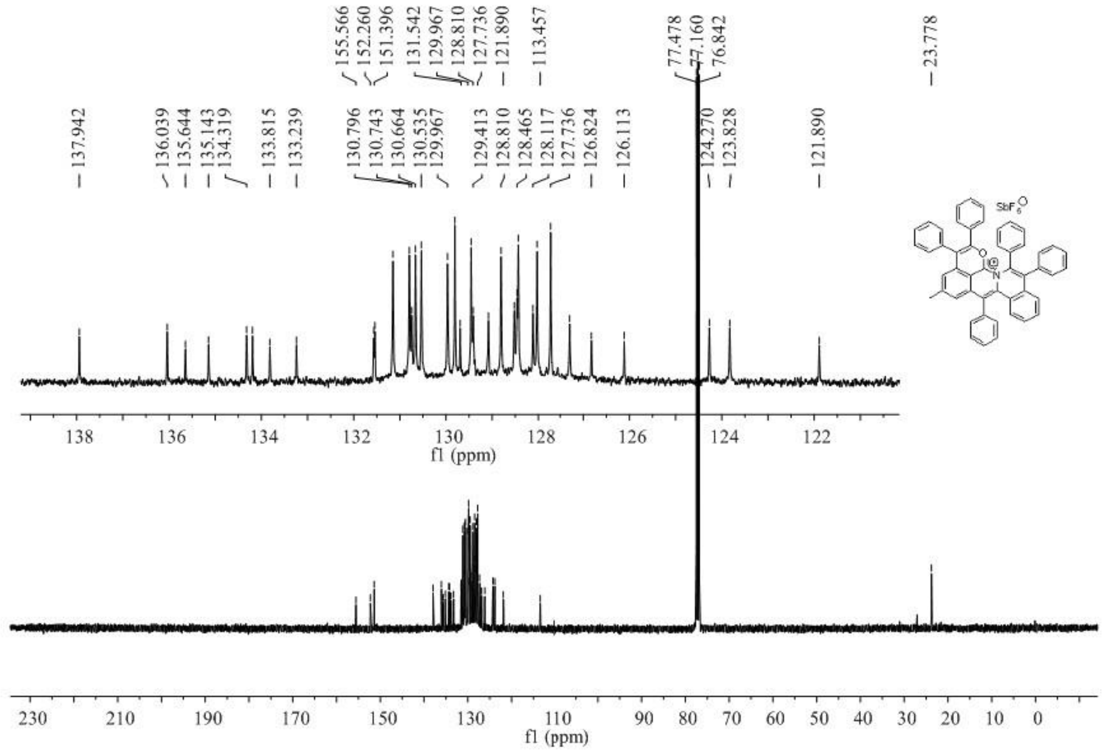

^{1}H NMR spectra of **3ca** ($CDCl_3$)

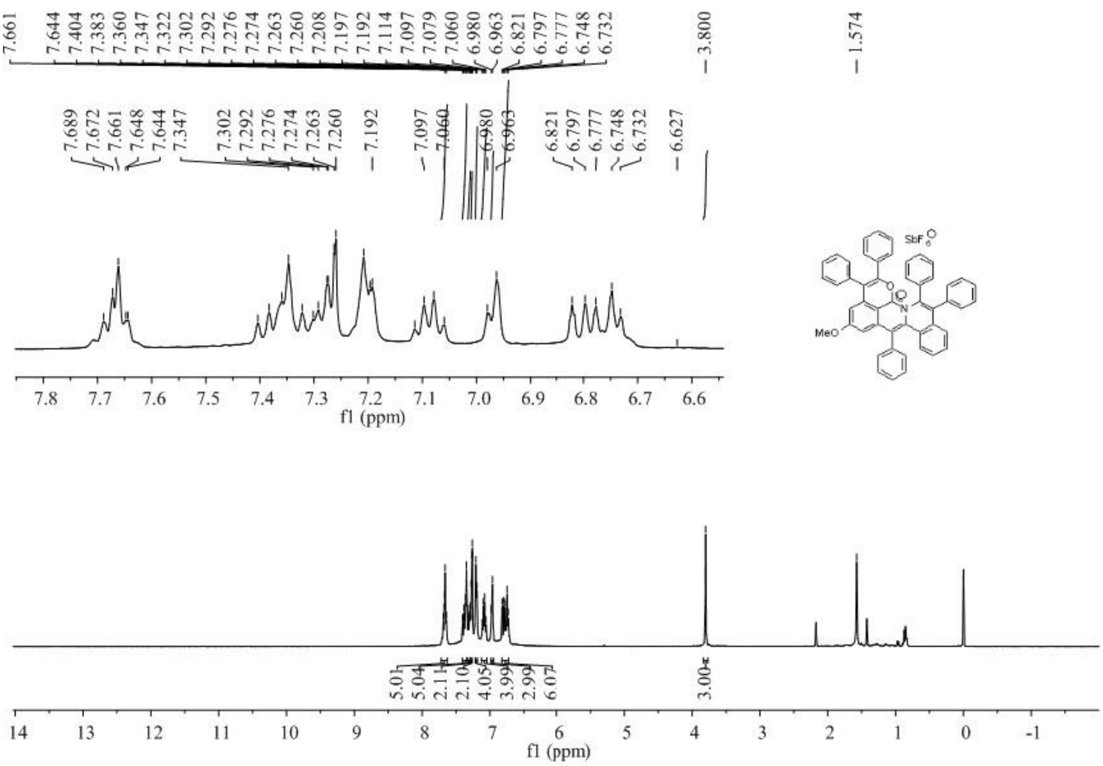

^{13}C NMR spectra of **3ca** ($CDCl_3$)

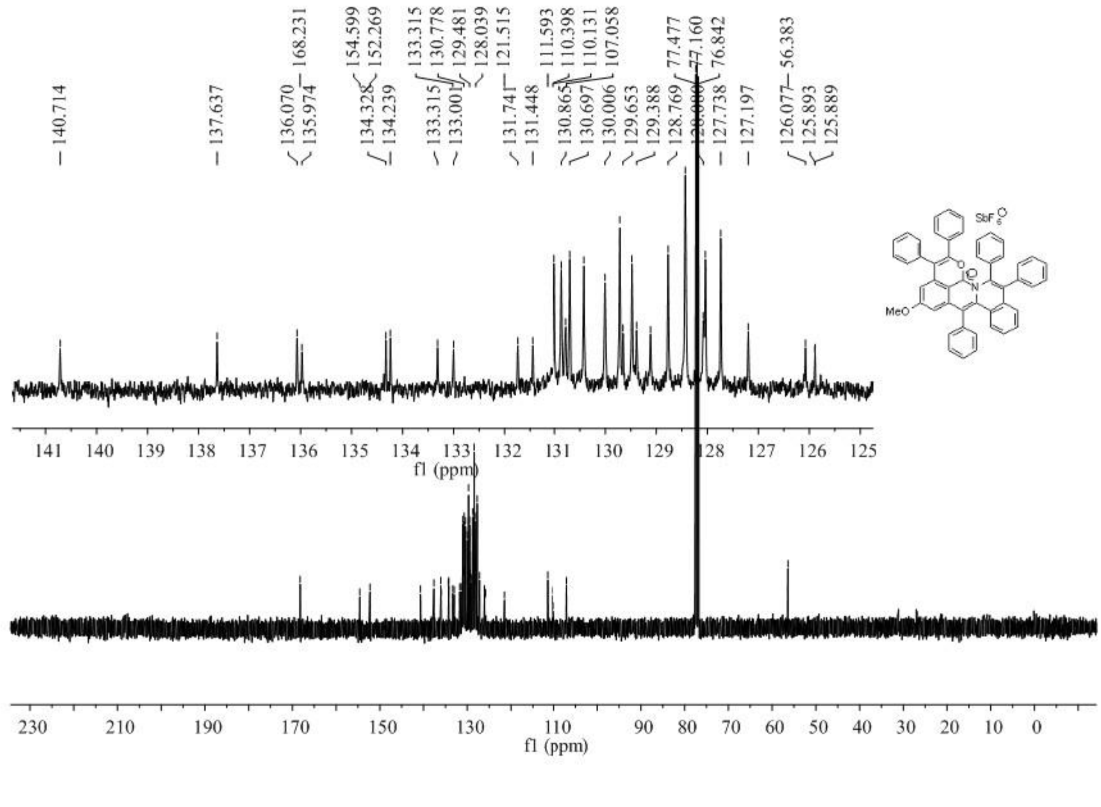

^{1}H NMR spectra of **3da** (CD_3CN)

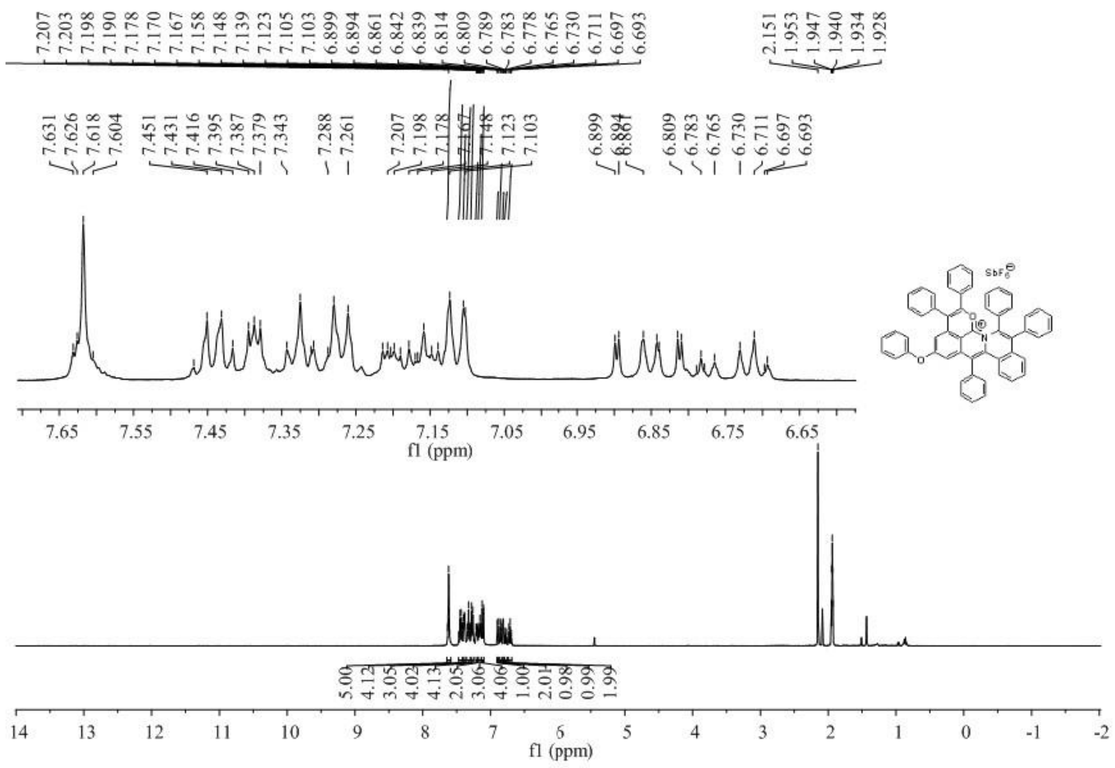

^{13}C NMR spectra of **3da** (CD_3CN)

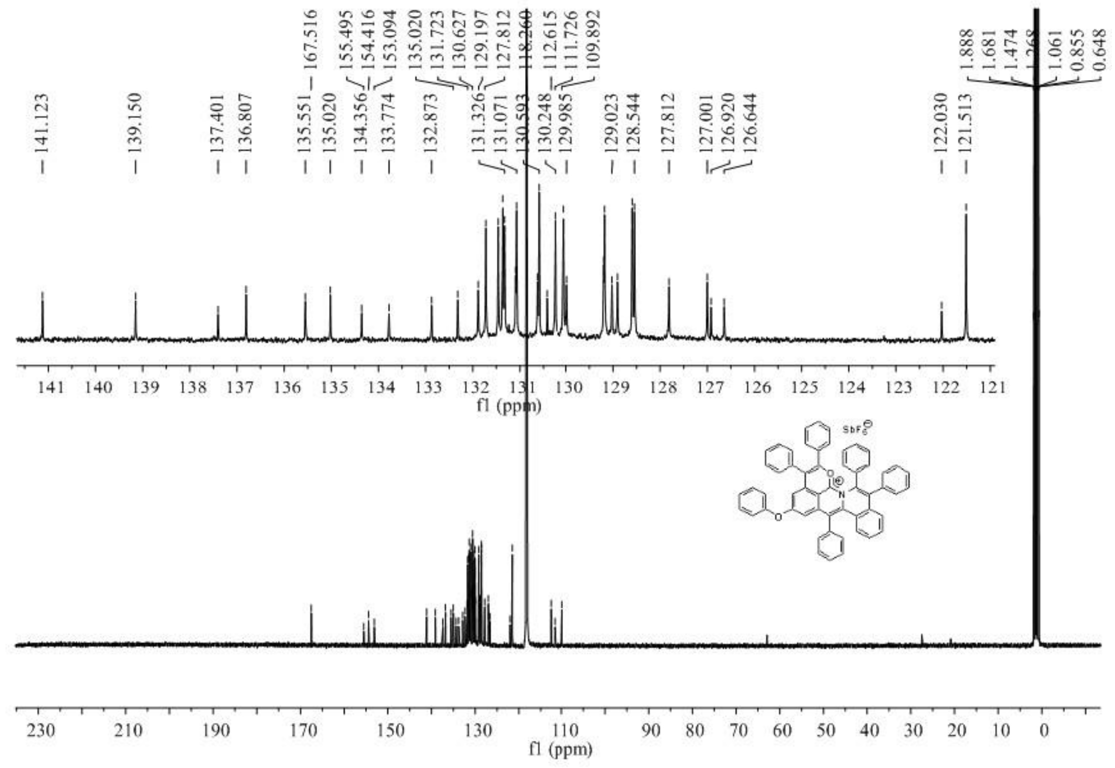

^{1}H NMR spectra of **3ea** ($CDCl_3$)

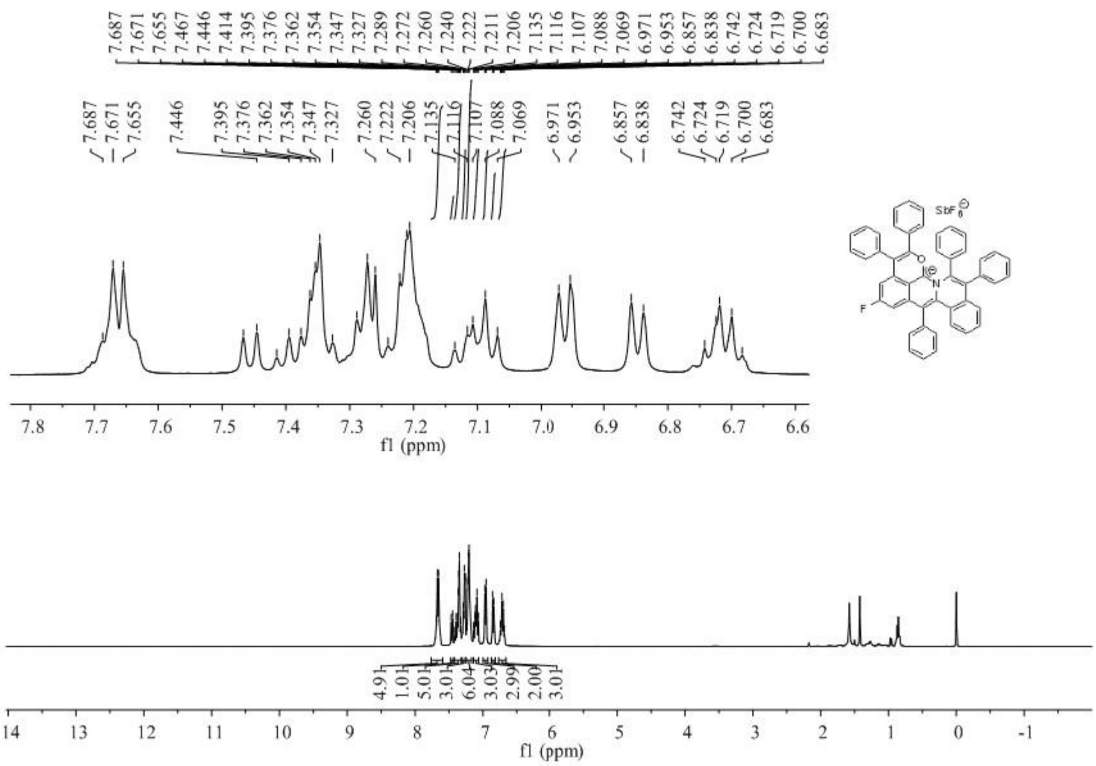

^{13}C NMR spectra of **3ea** ($CDCl_3$)

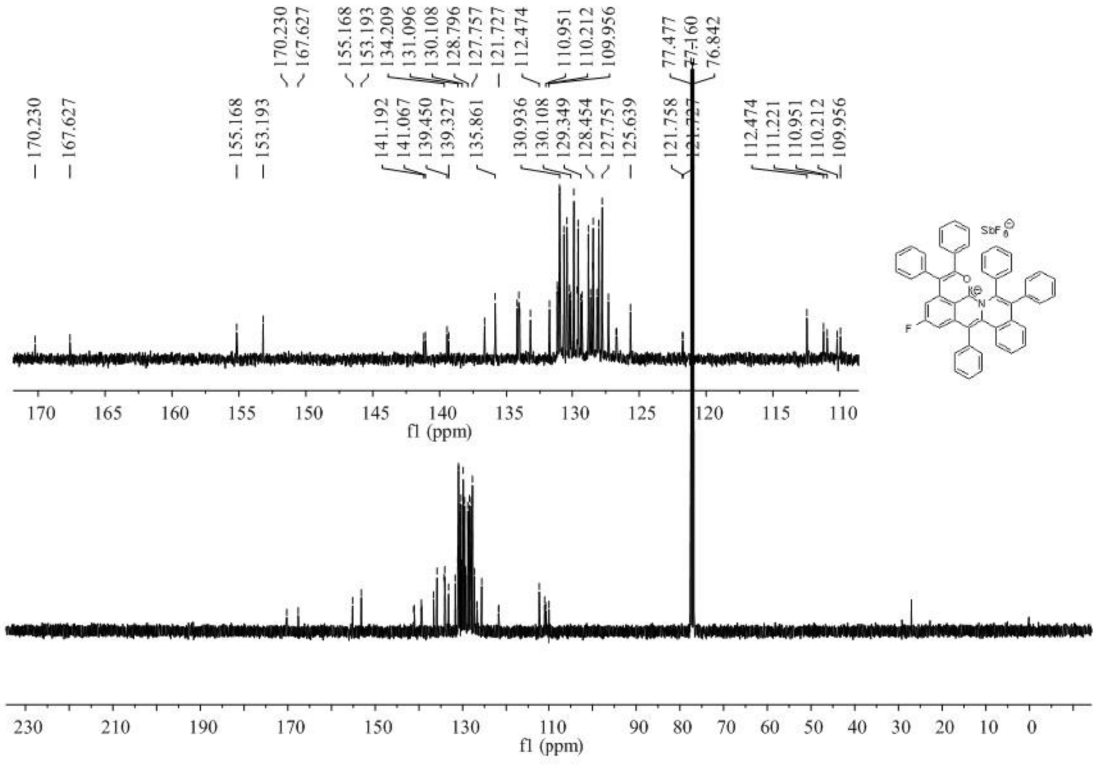

^{19}F NMR spectra of **3ea** ($CDCl_3$)

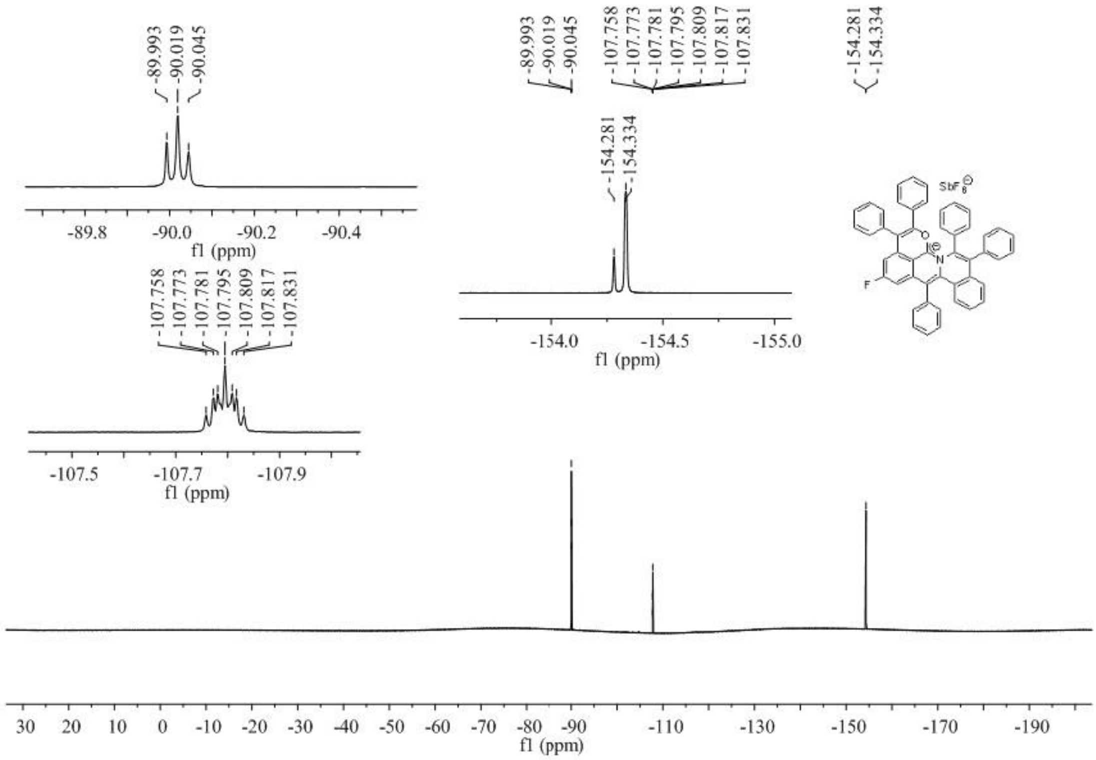

^{1}H NMR spectra of **3fa** ($CDCl_3$)

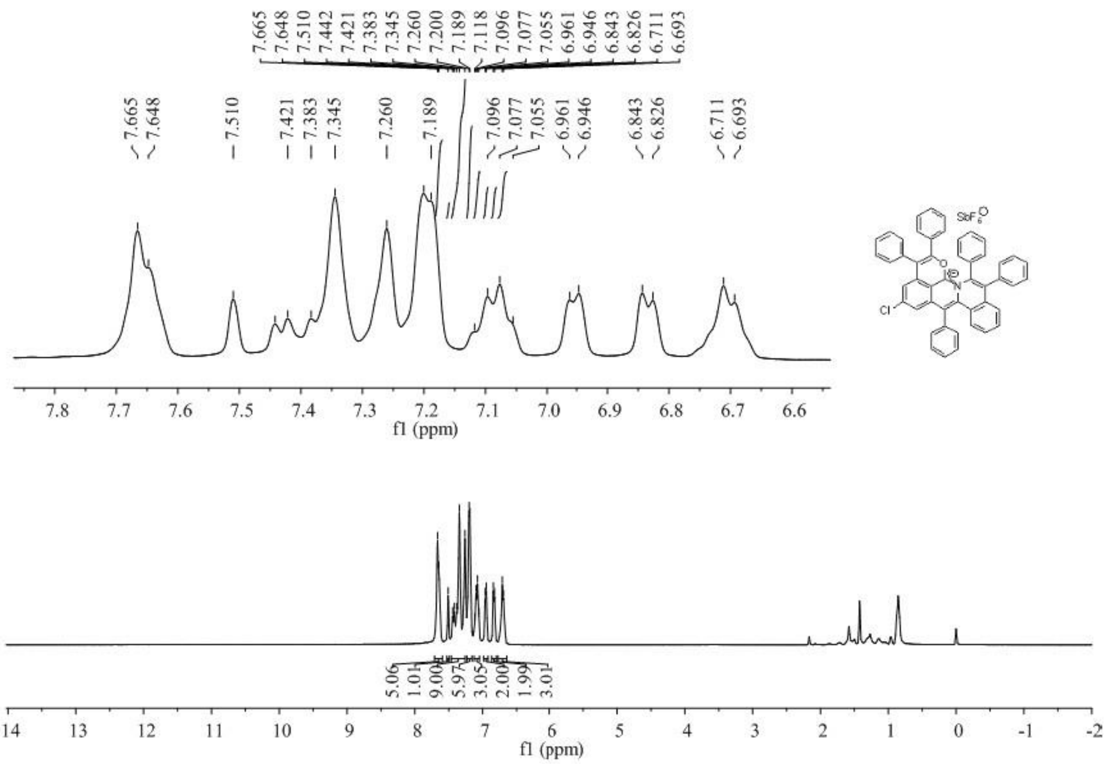

^{13}C NMR spectra of **3fa** ($CDCl_3$)

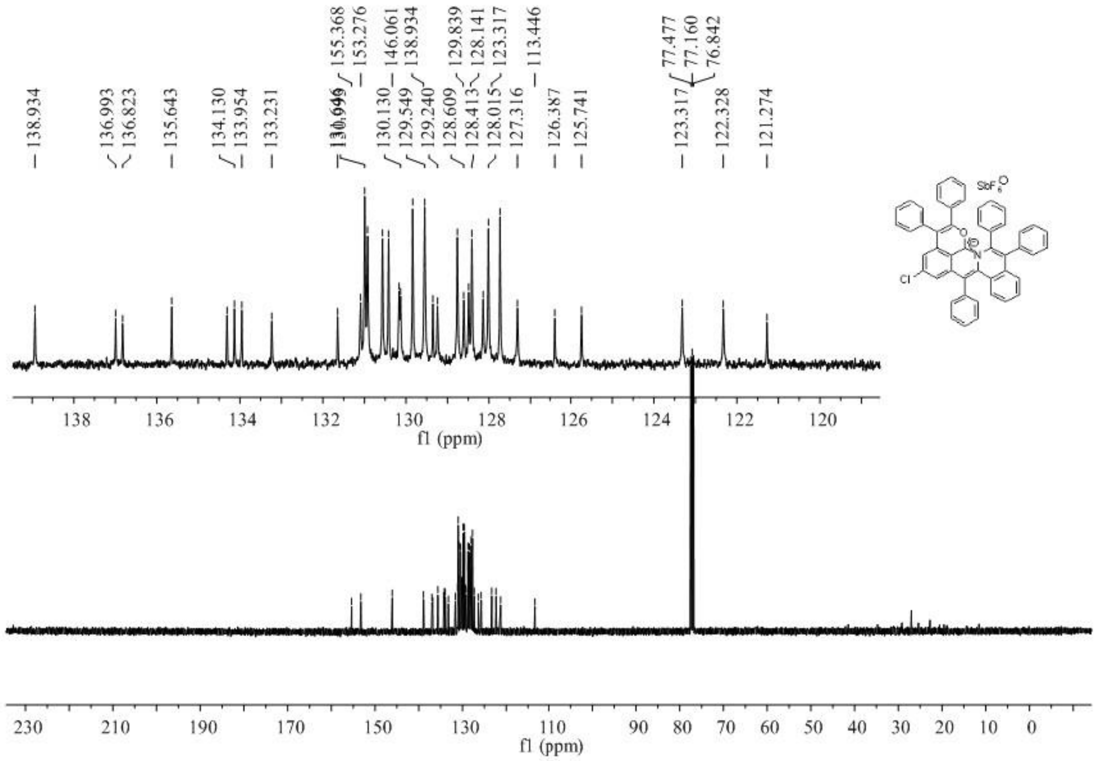

^{1}H NMR spectra of **3ga** ($CDCl_3$)

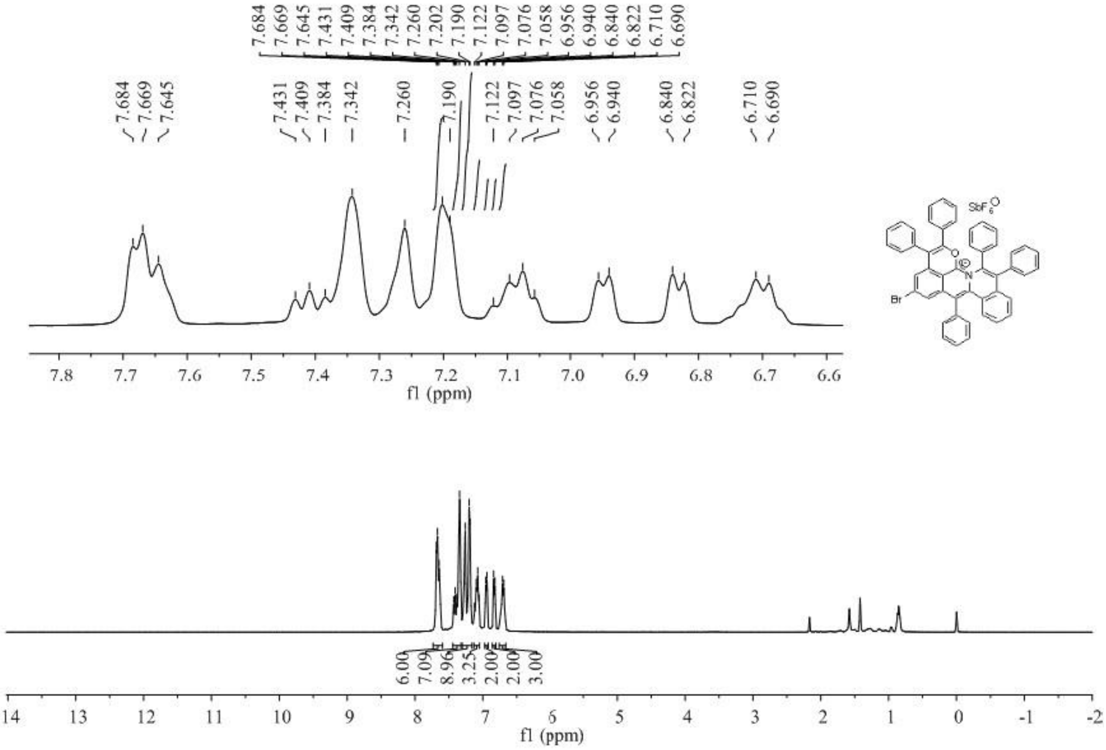

^{13}C NMR spectra of **3ga** ($CDCl_3$)

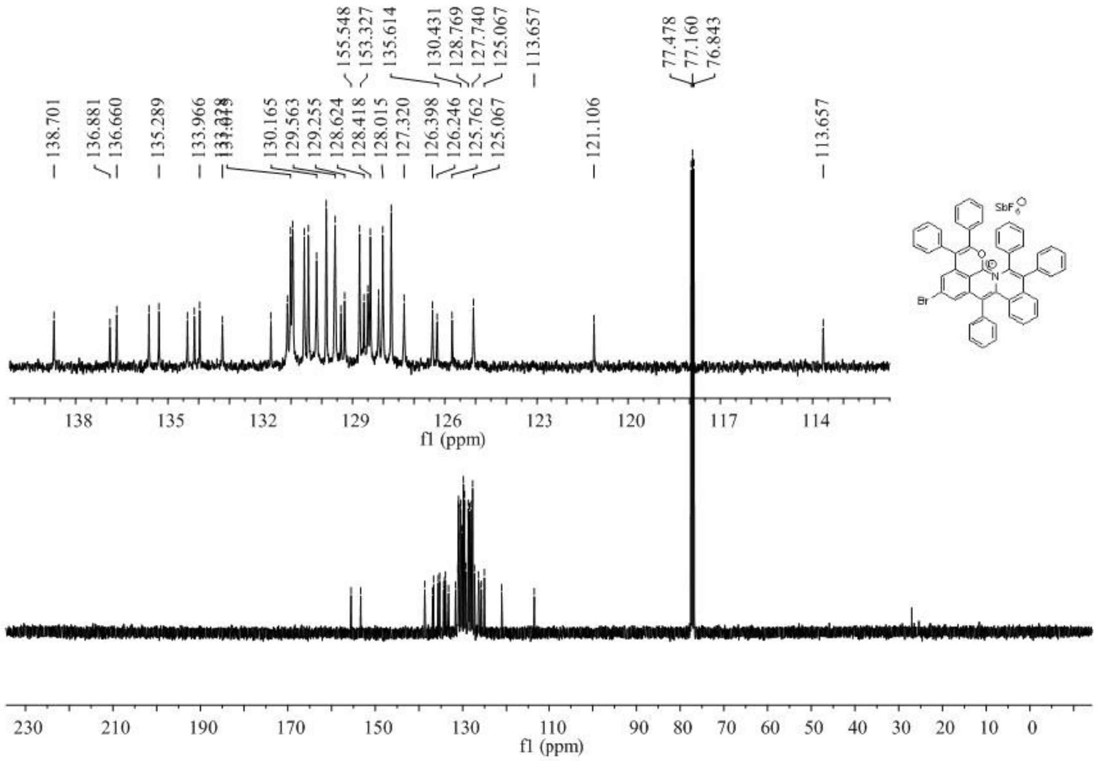

^{1}H NMR spectra of **3ha** ($CDCl_3$)

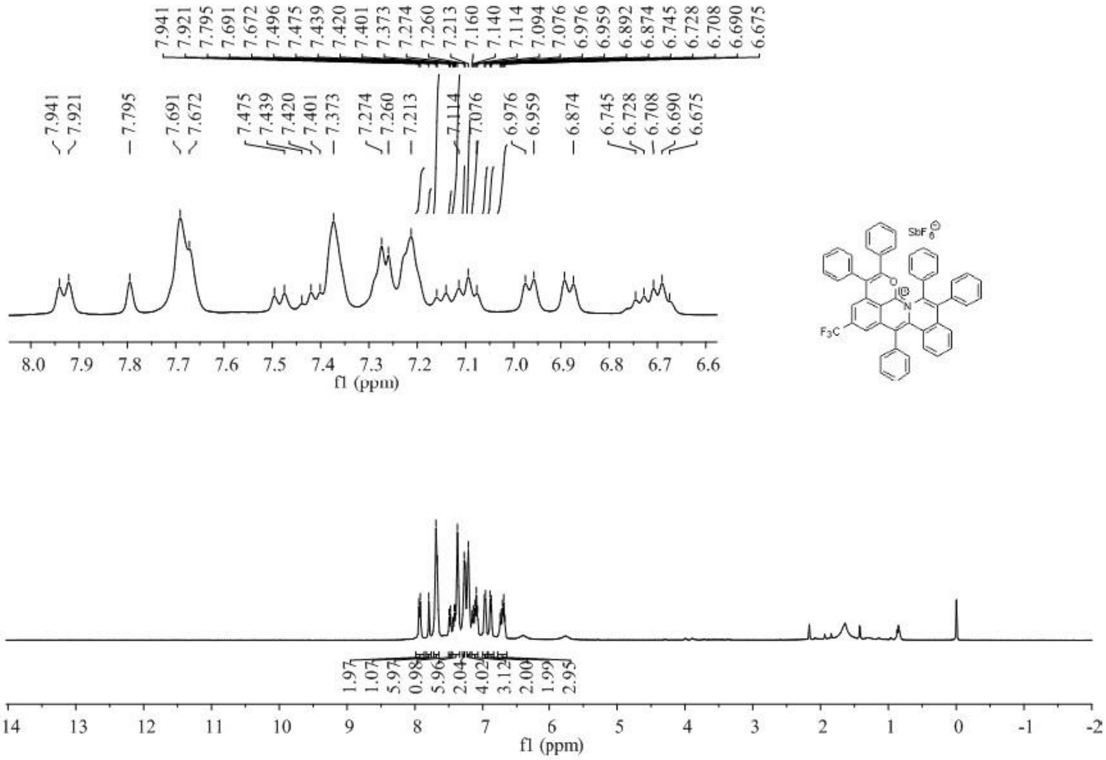

^{13}C NMR spectra of **3ha** ($CDCl_3$)

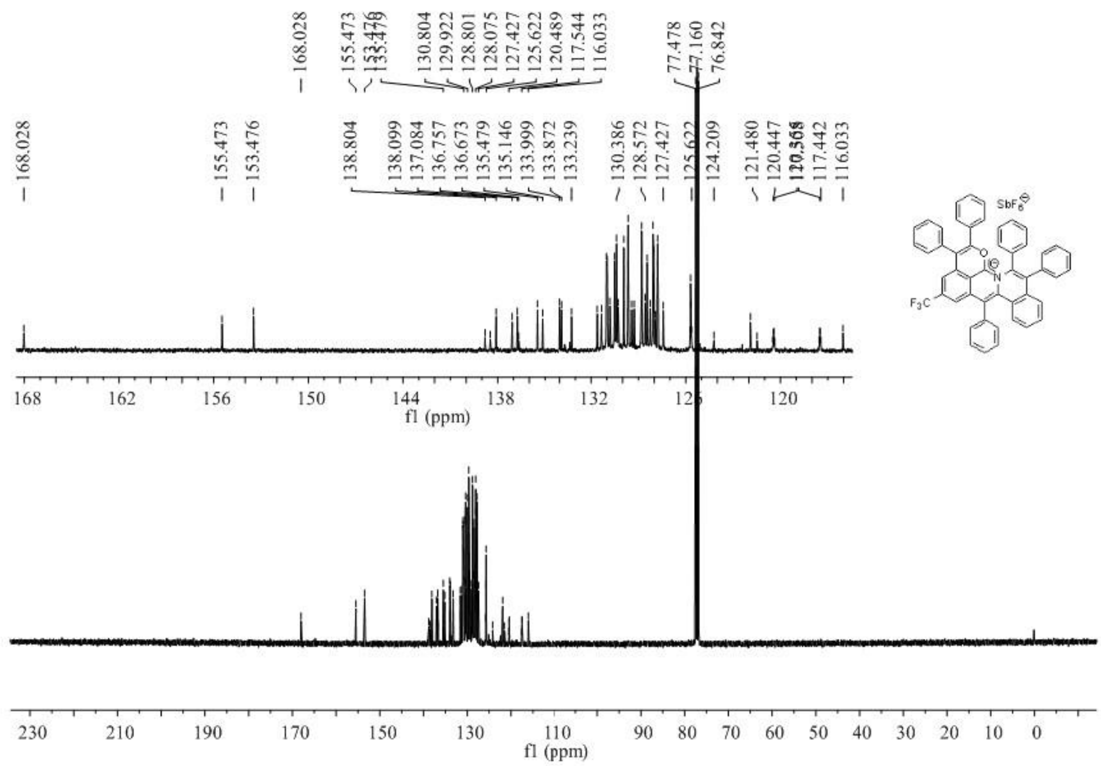

^{19}F NMR spectra of **3ha** ($CDCl_3$)

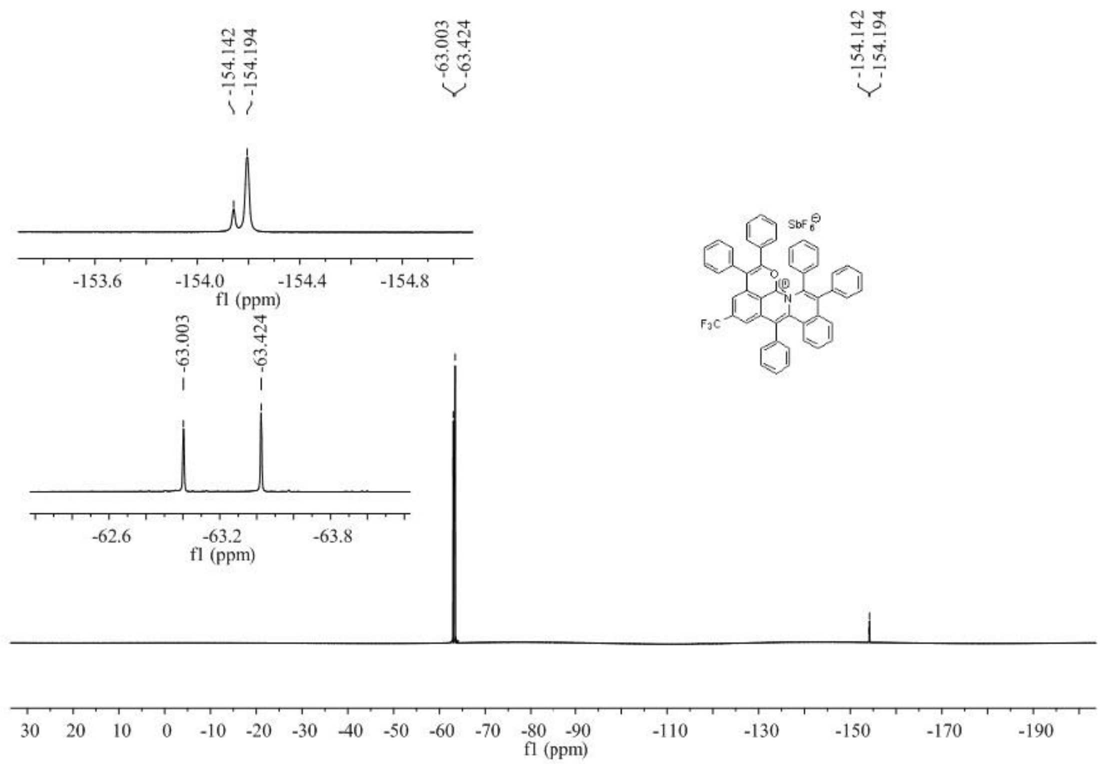

^{1}H NMR spectra of **3ia** ($CDCl_3$)

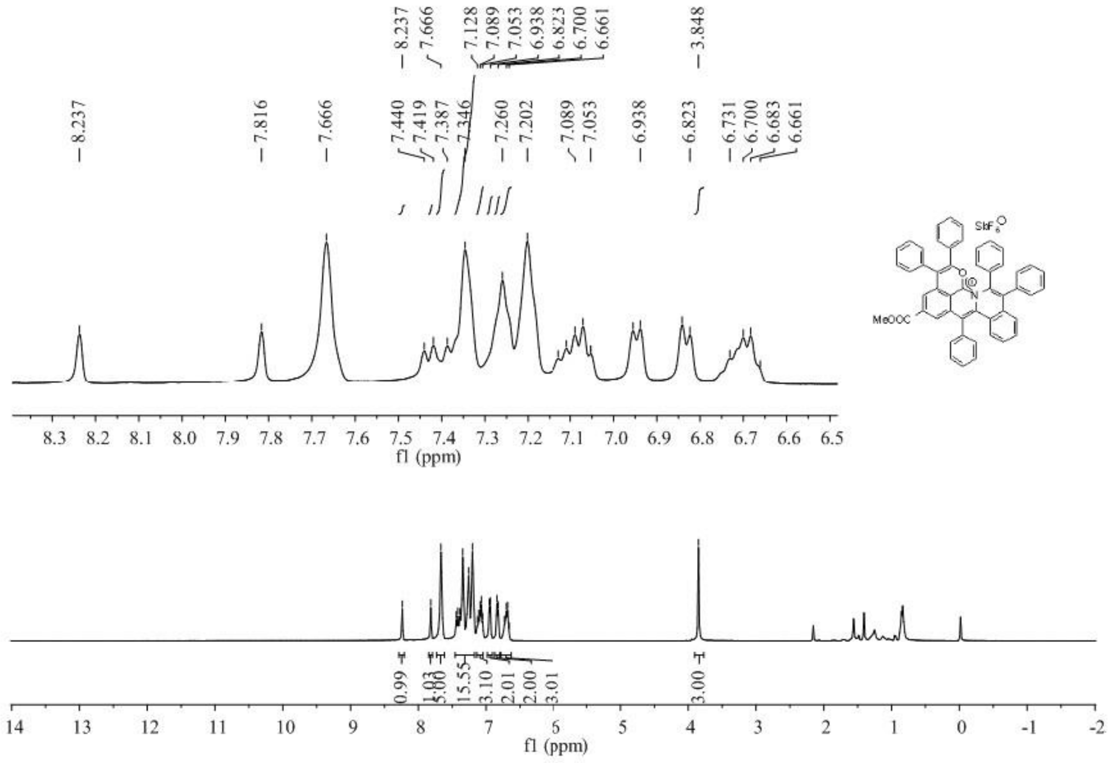

^{13}C NMR spectra of **3ia** ($CDCl_3$)

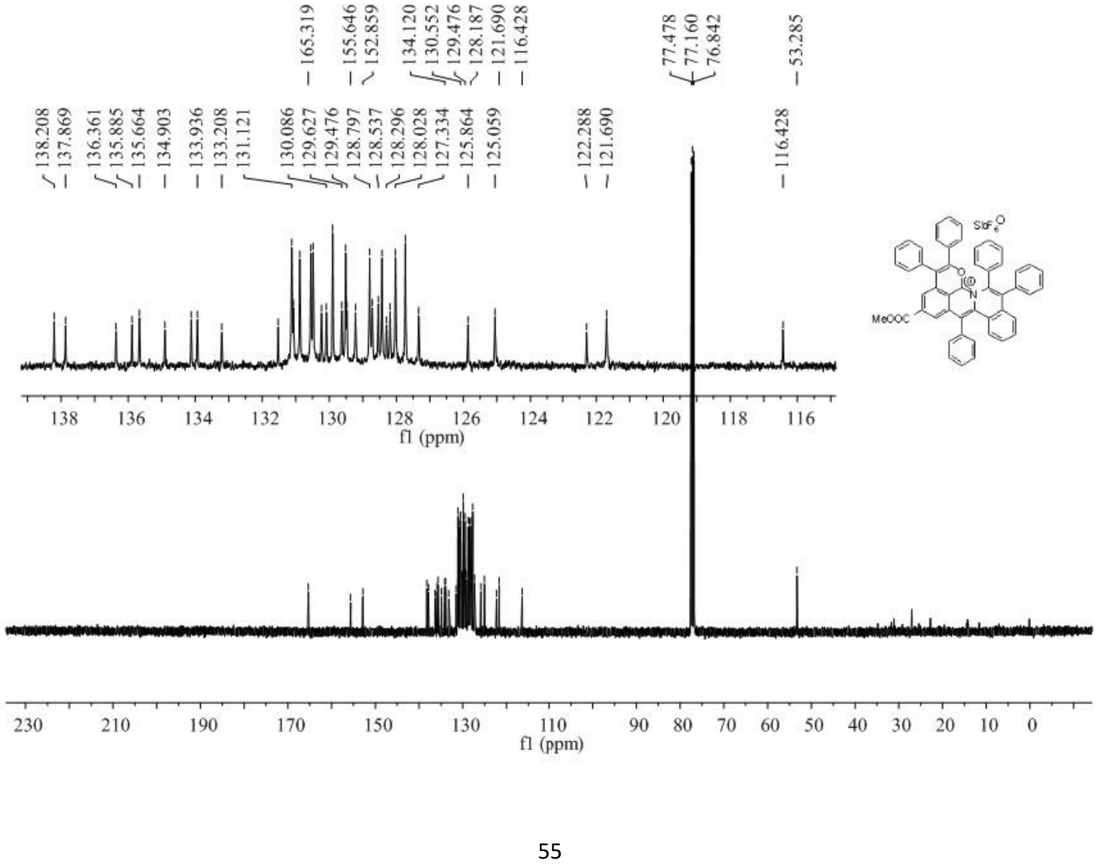

^{1}H NMR spectra of **3ja** ($CDCl_3$)

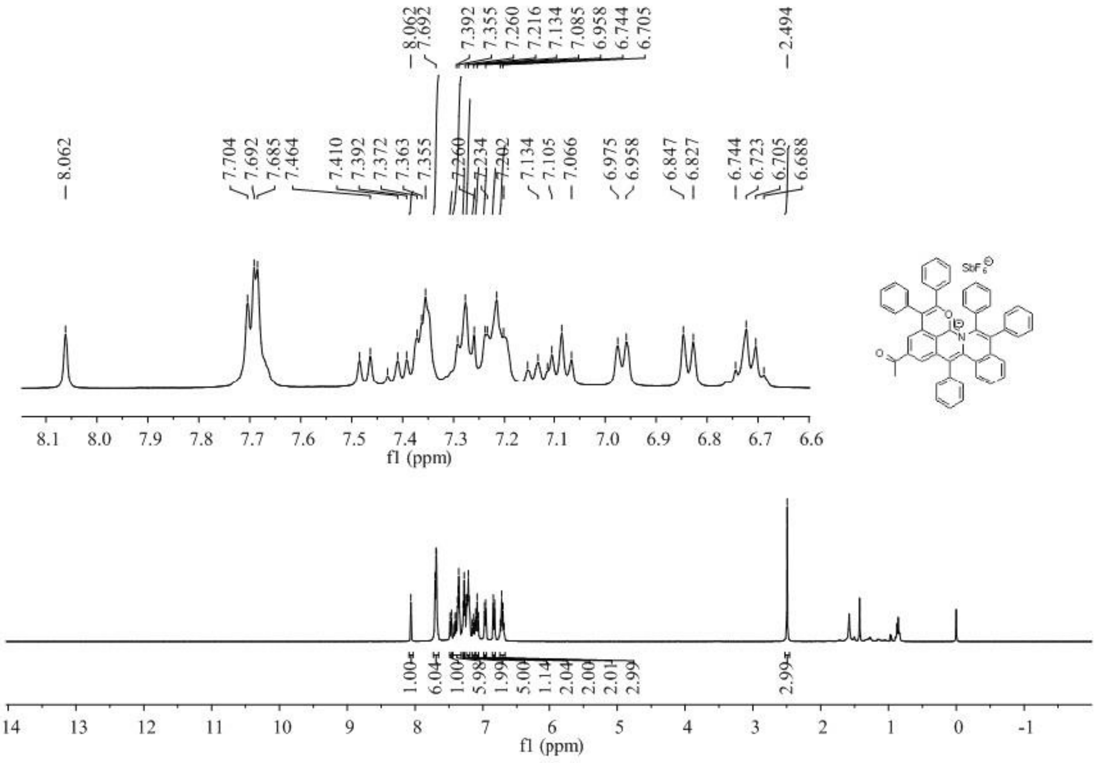

^{13}C NMR spectra of **3ja** ($CDCl_3$)

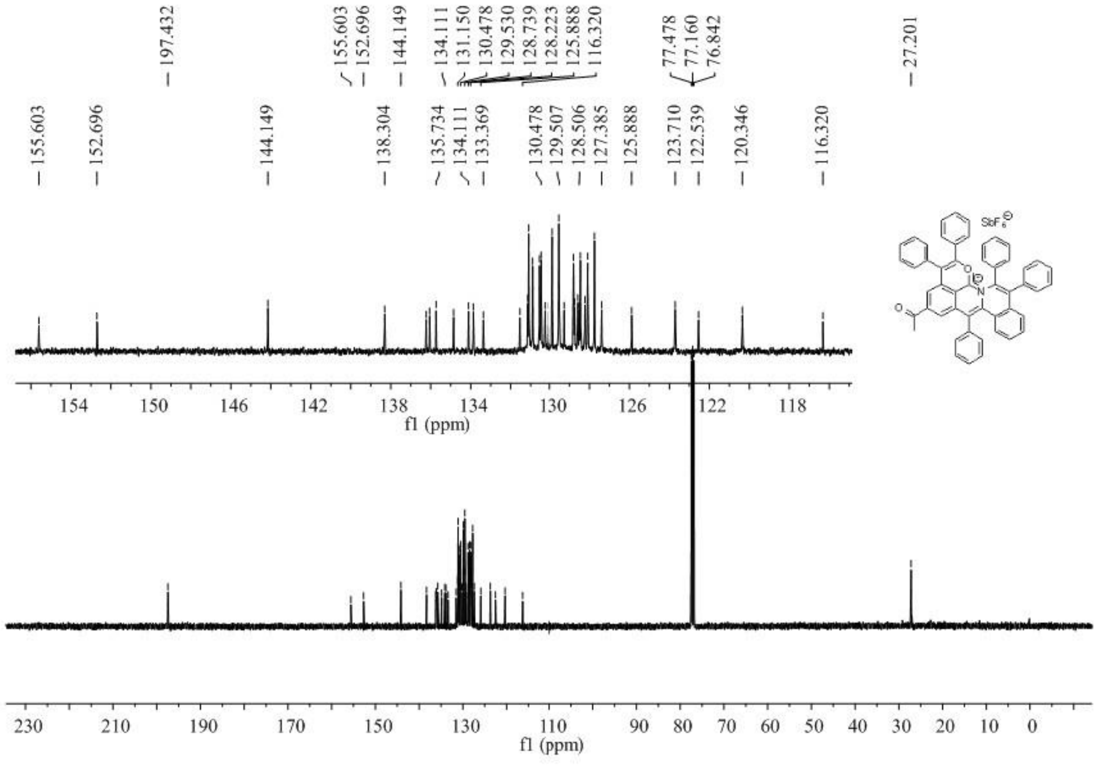

^{1}H NMR spectra of **3ka** ($CDCl_3$)

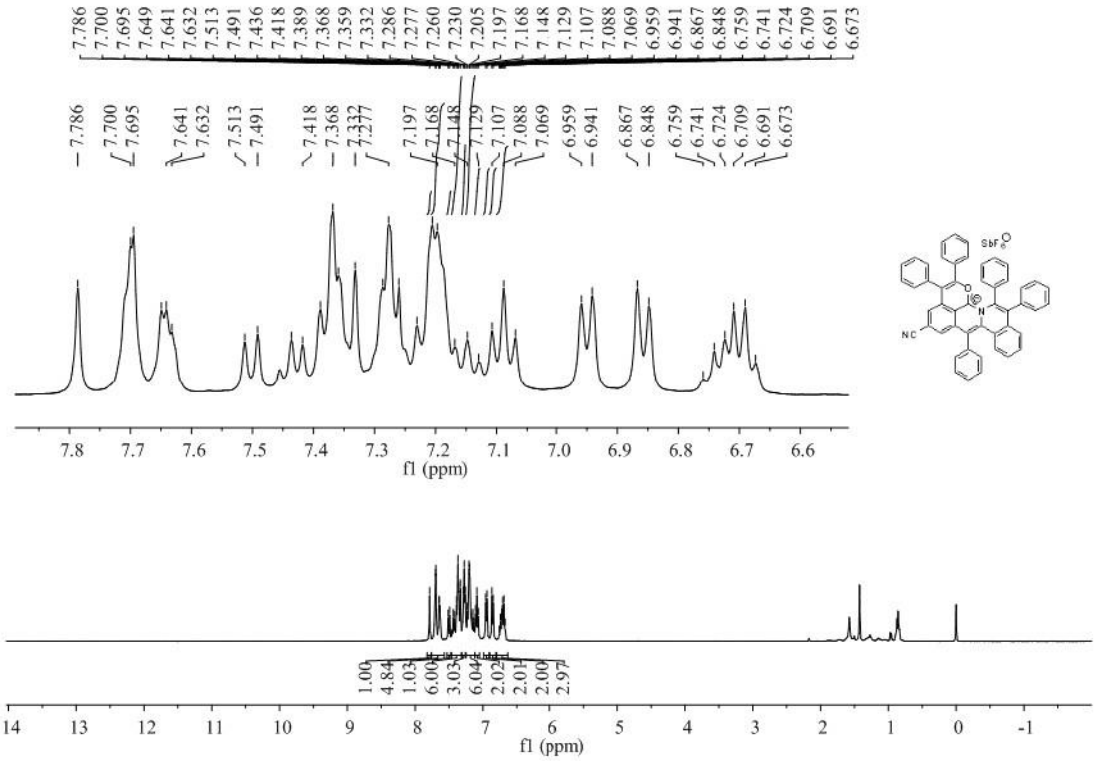

^{13}C NMR spectra of **3ka** ($CDCl_3$)

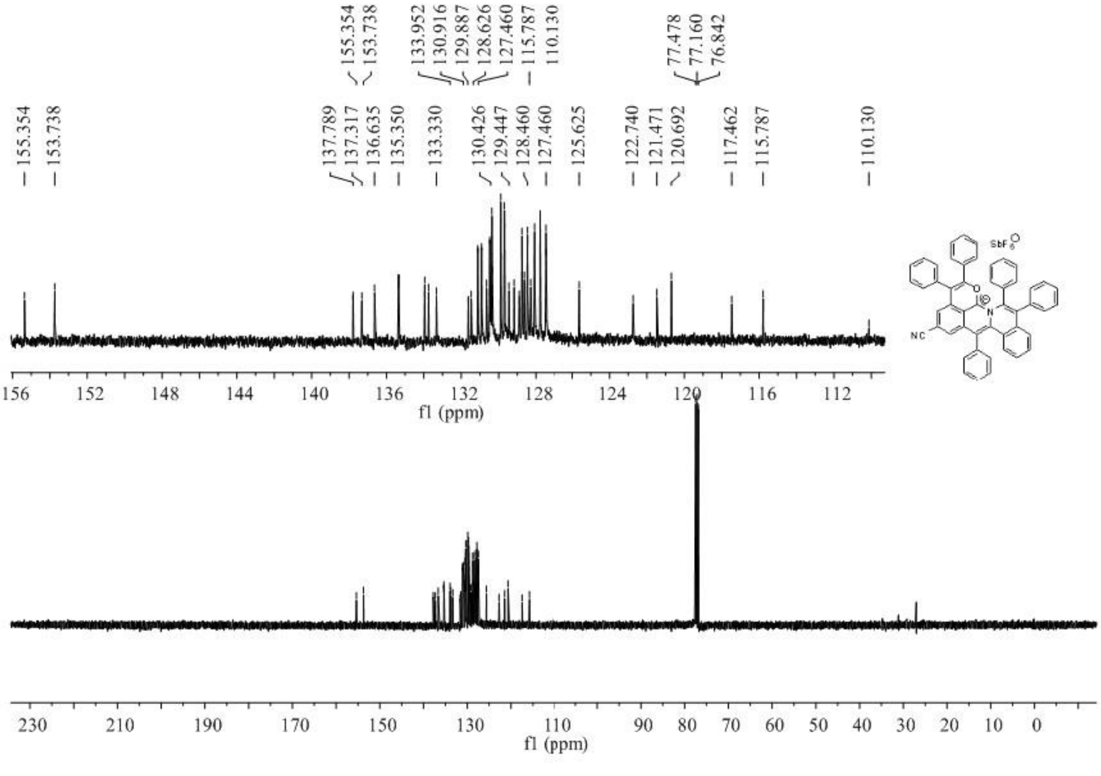

^{1}H NMR spectra of **3la** ($CDCl_3$)

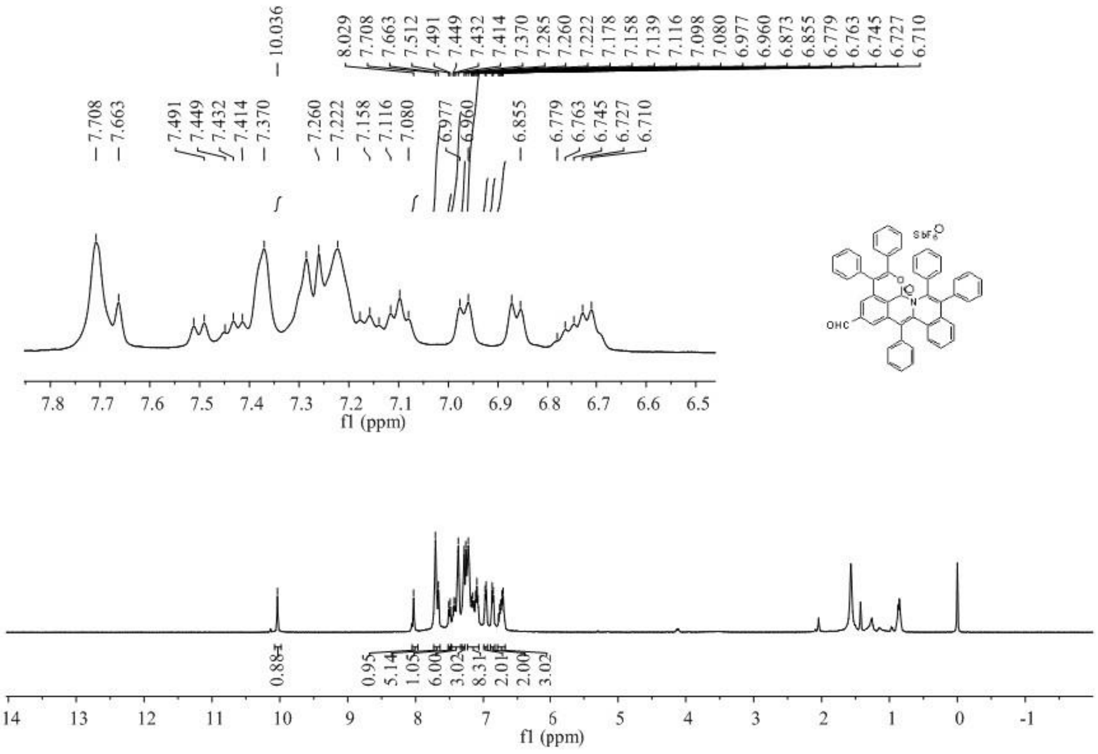

^{13}C NMR spectra of **3la** ($CDCl_3$)

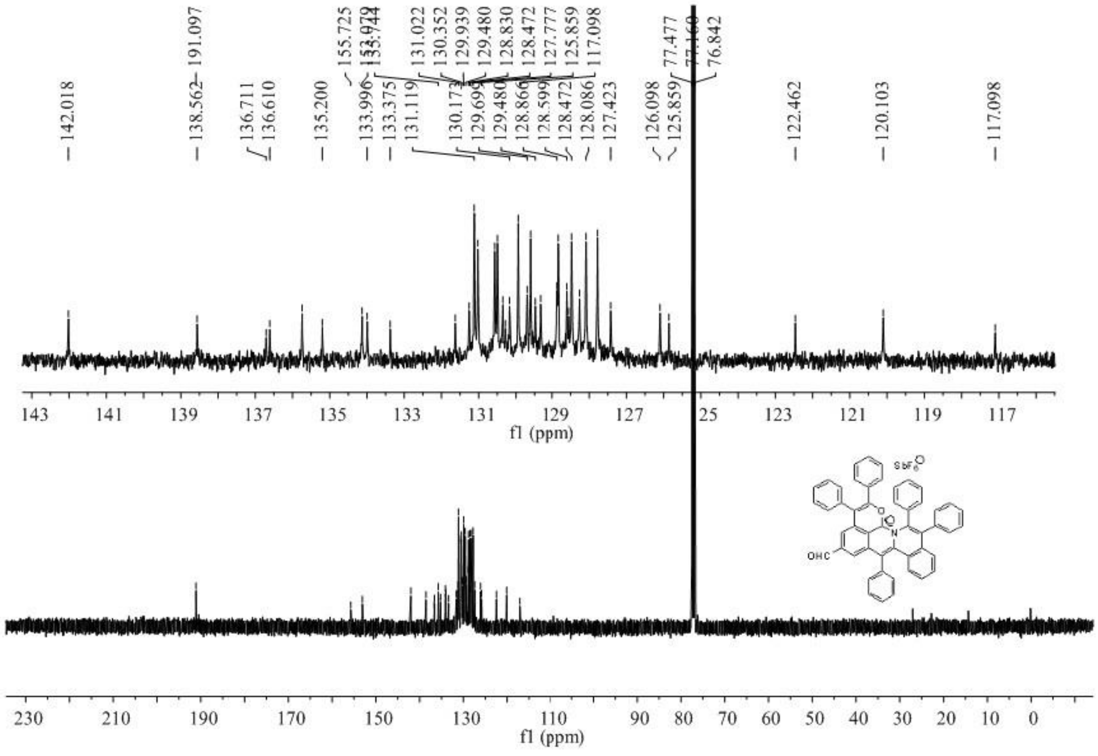

^{1}H NMR spectra of **3ma** ($CDCl_3$)

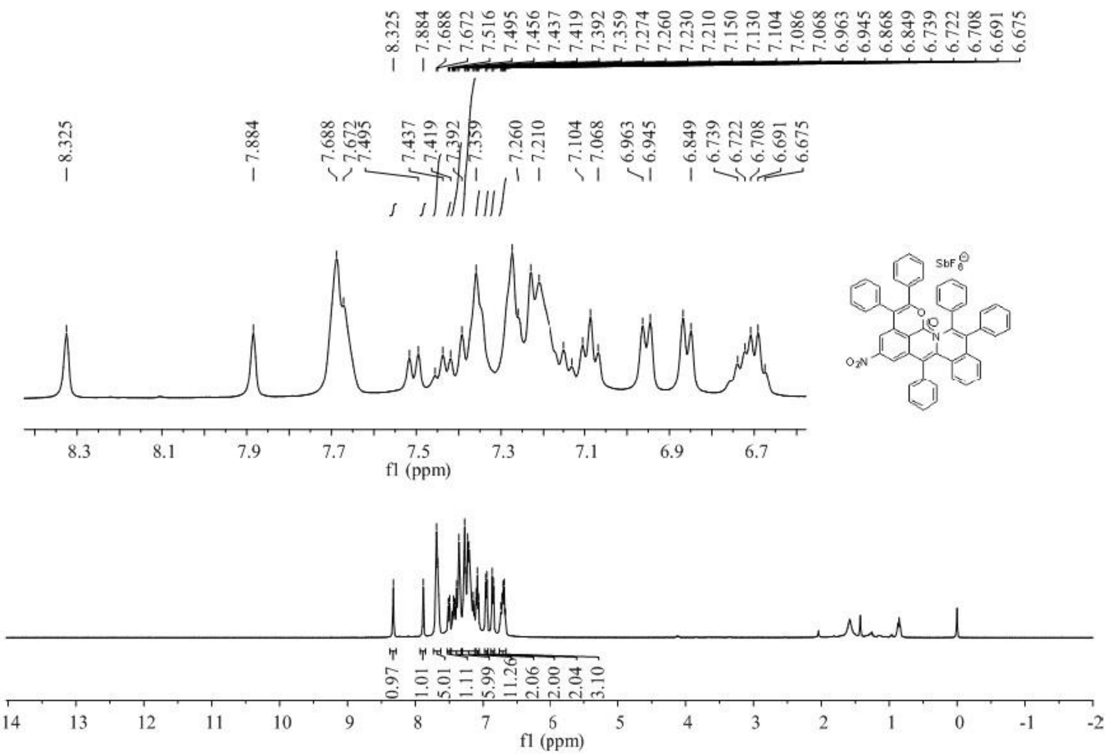

^{13}C NMR spectra of **3ma** ($CDCl_3$)

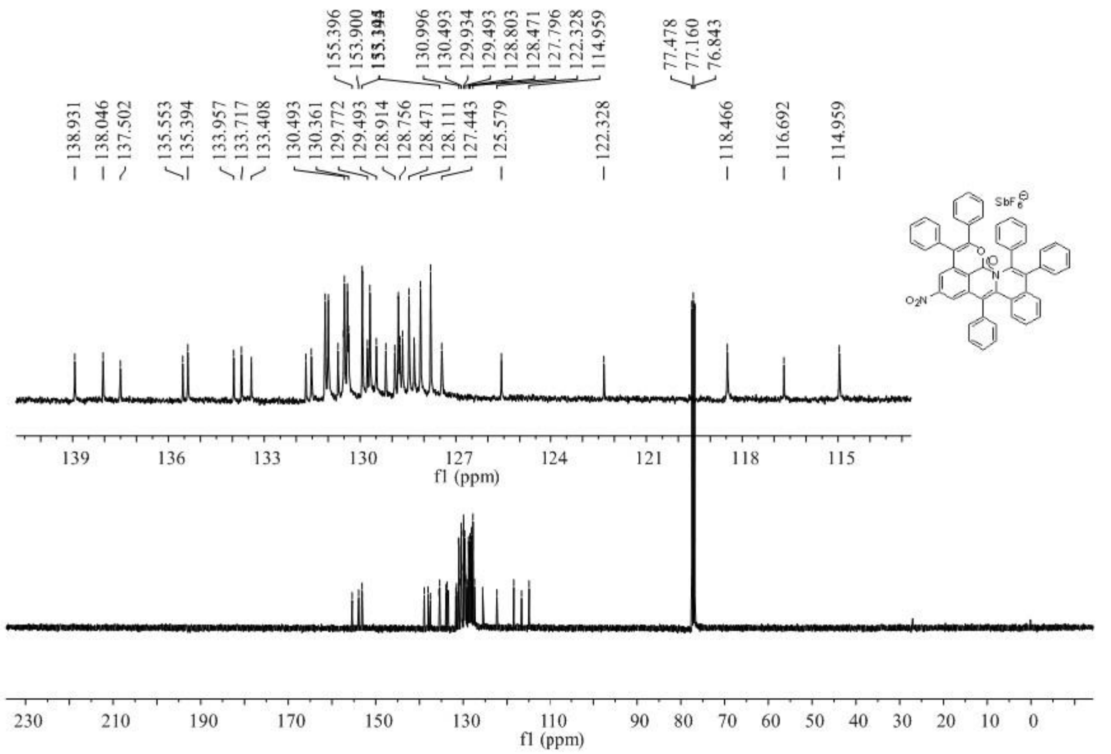

^{1}H NMR spectra of **3na** ($CDCl_3$)

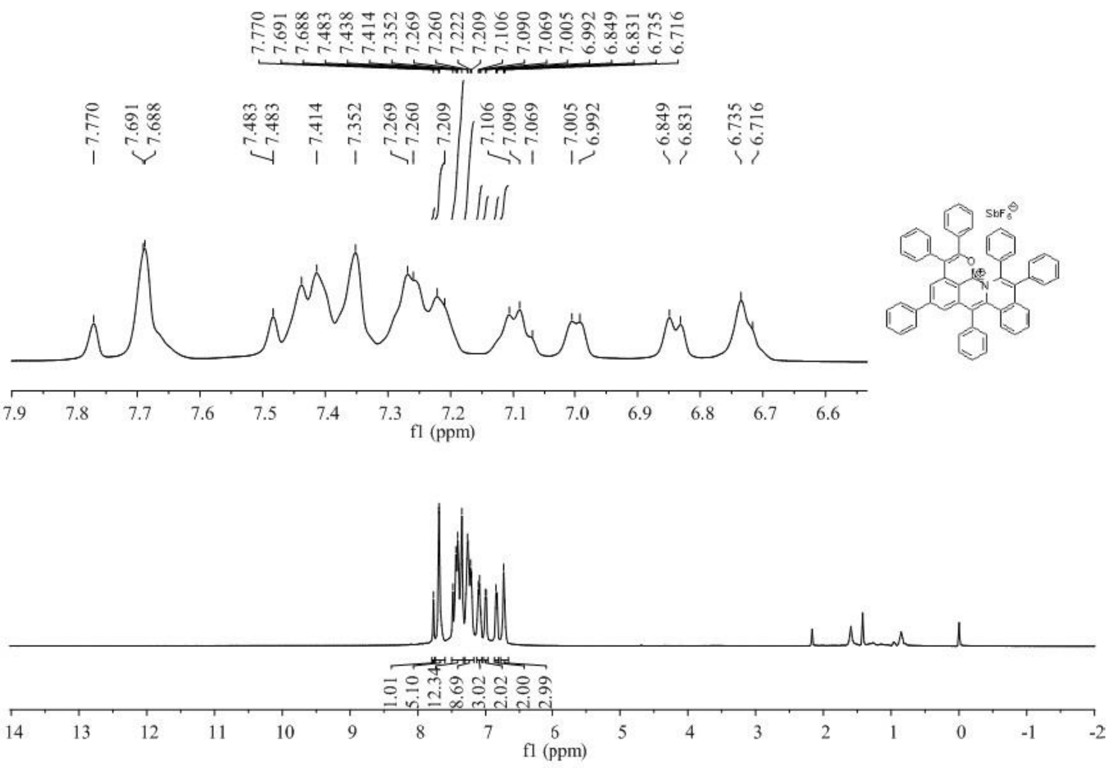

^{13}C NMR spectra of **3na** ($CDCl_3$)

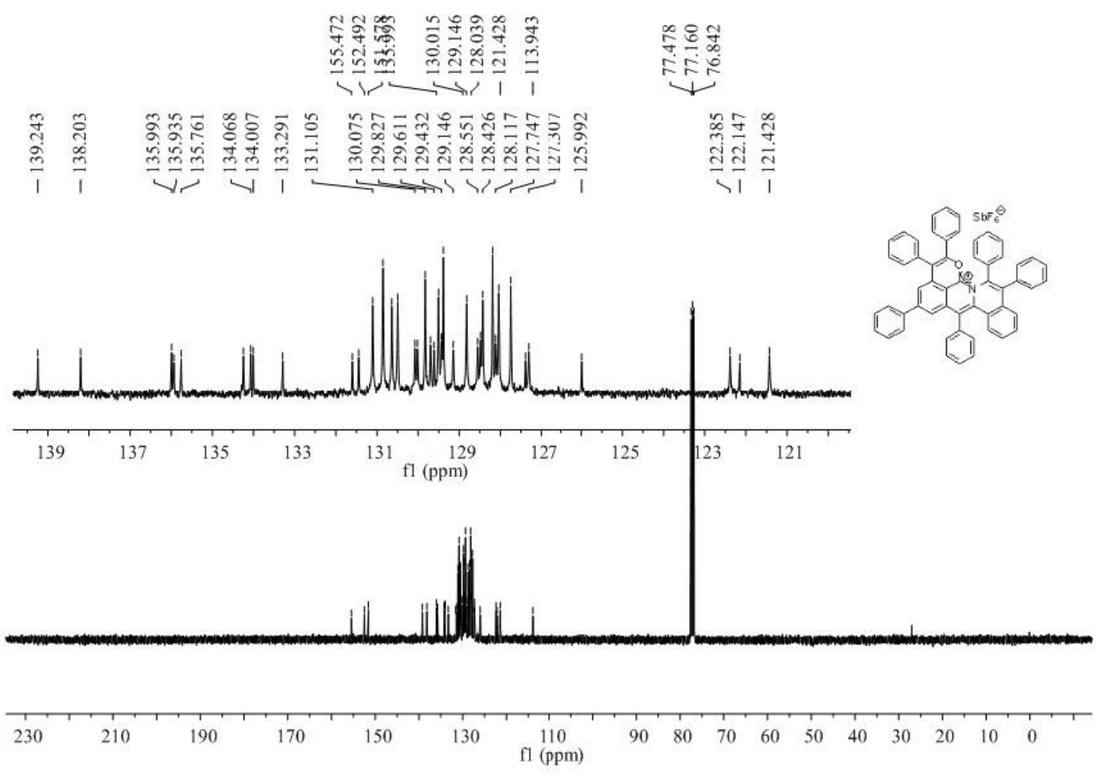

^{1}H NMR spectra of **3oa** ($CDCl_3$)

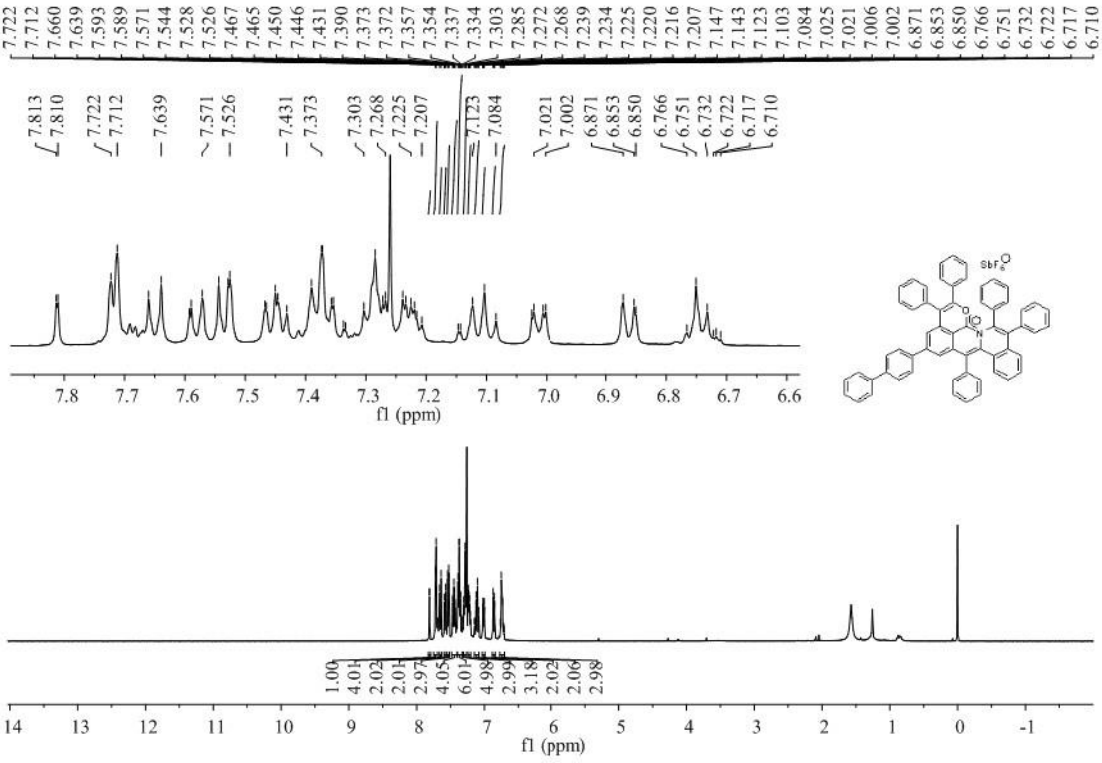

^{13}C NMR spectra of **3oa** ($CDCl_3$)

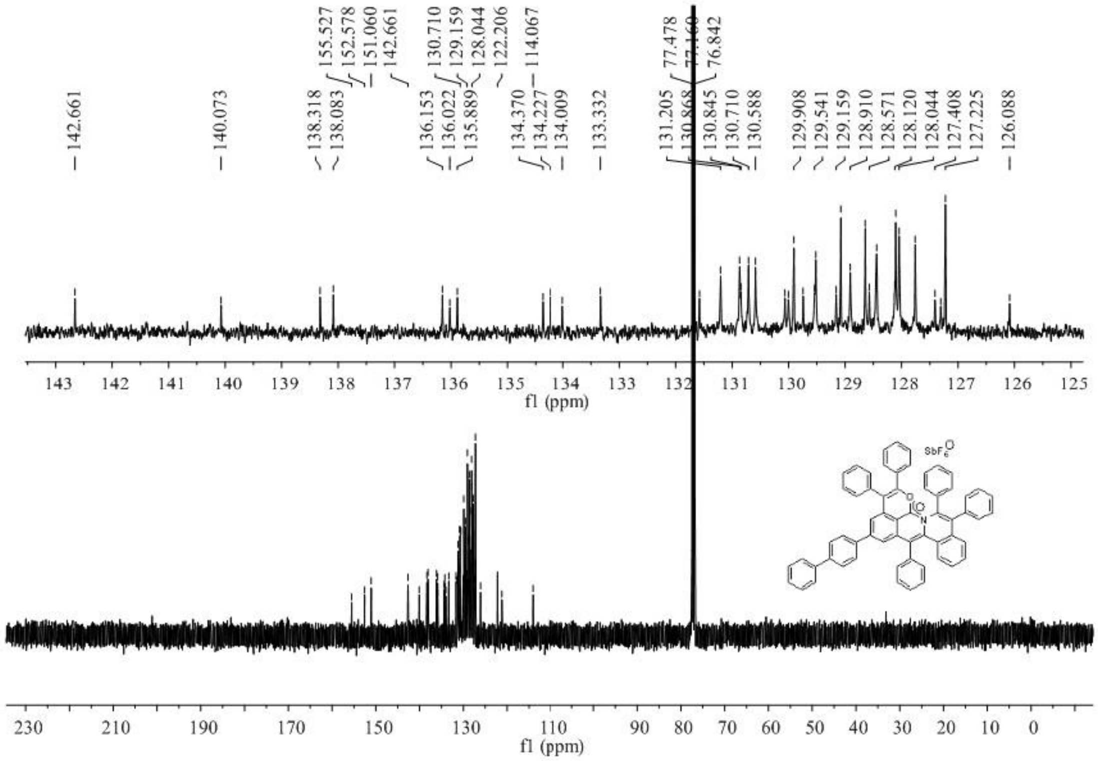

^{1}H NMR spectra of **3pa** ($CDCl_3$)

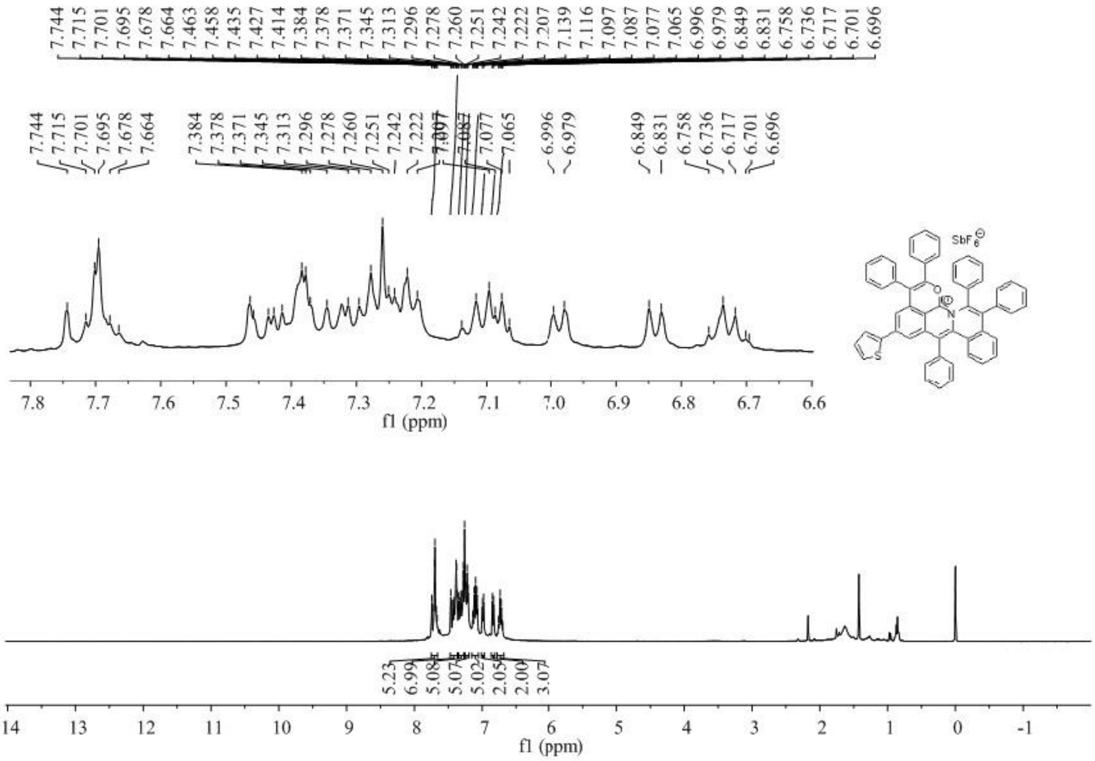

^{13}C NMR spectra of **3pa** ($CDCl_3$)

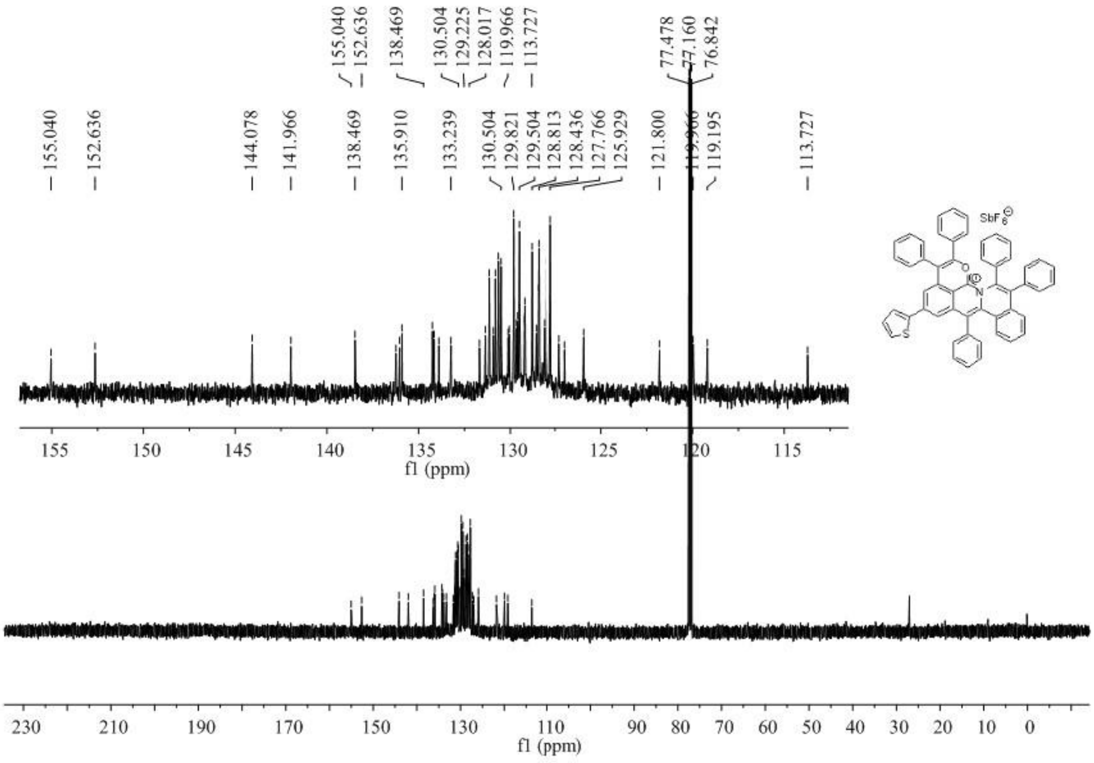

^{1}H NMR spectra of **3qa** ($CDCl_3$)

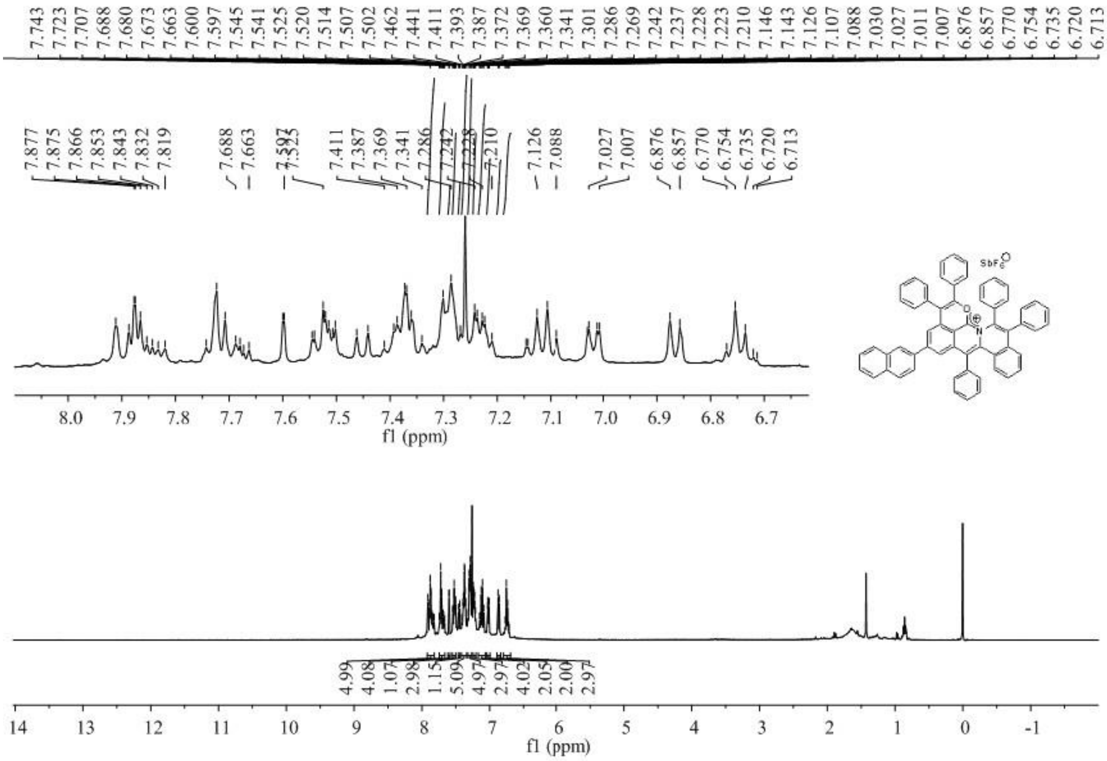

^{13}C NMR spectra of **3qa** ($CDCl_3$)

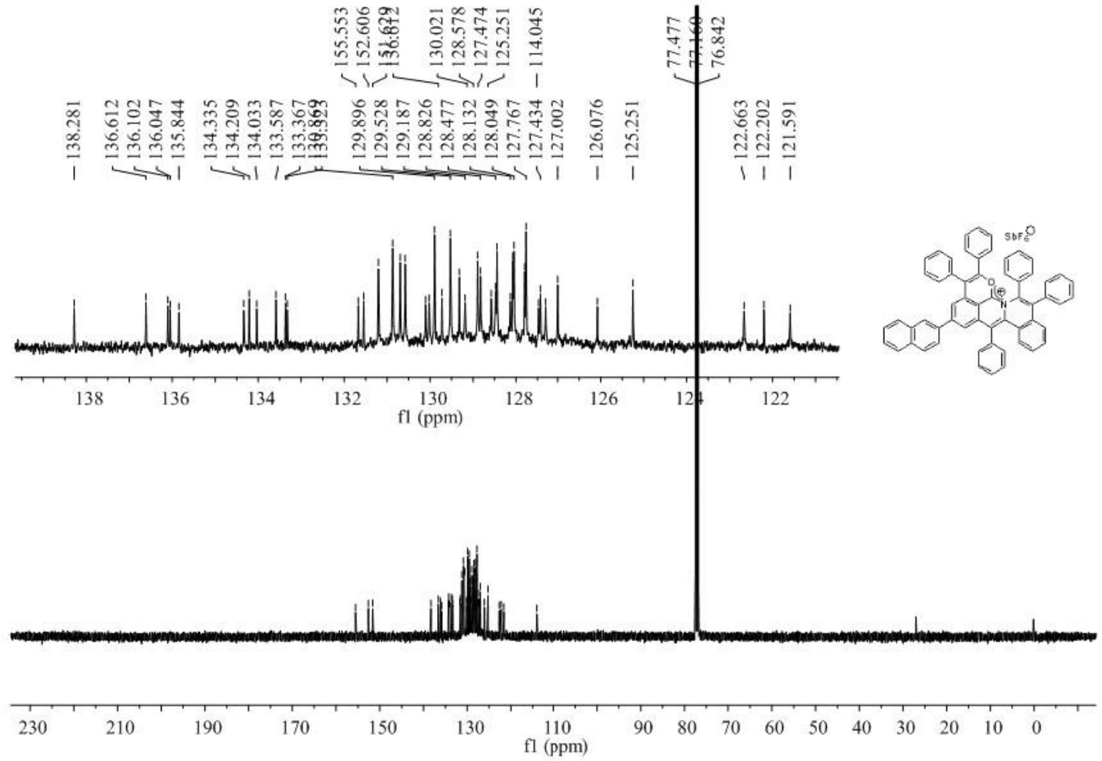

^{1}H NMR spectra of **3ra** and its isomer ($CDCl_3$)

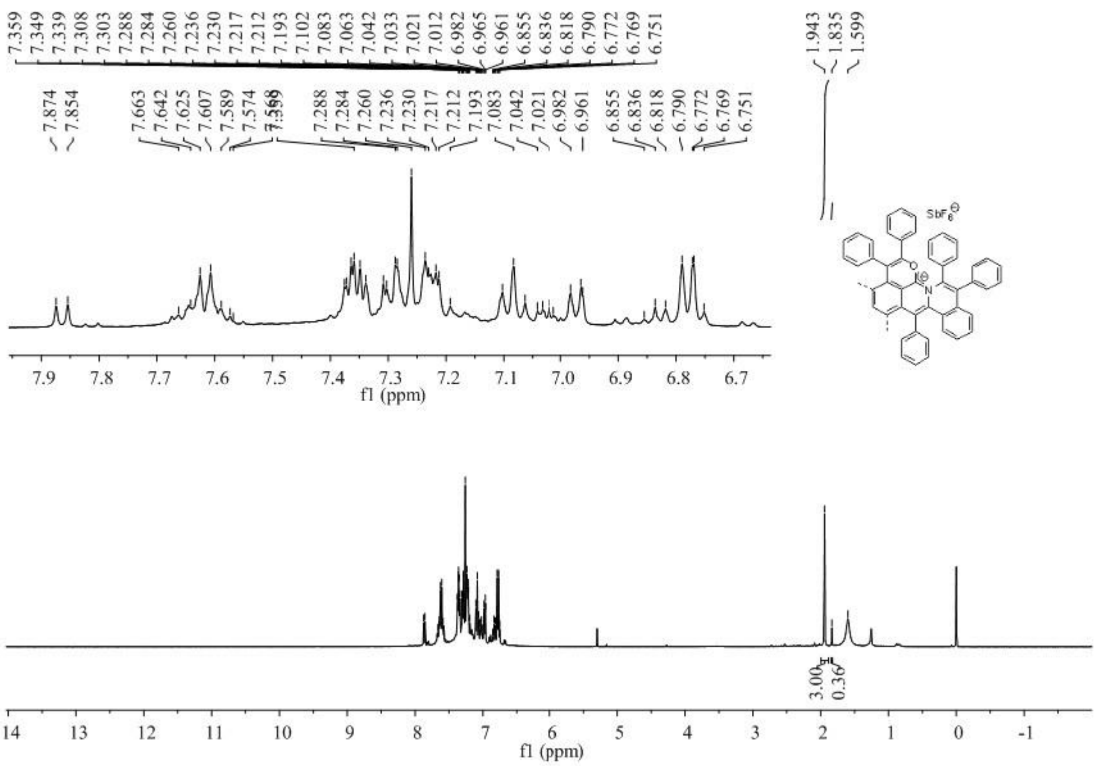

^{1}H NMR spectra of **3ab** ($CDCl_3$)

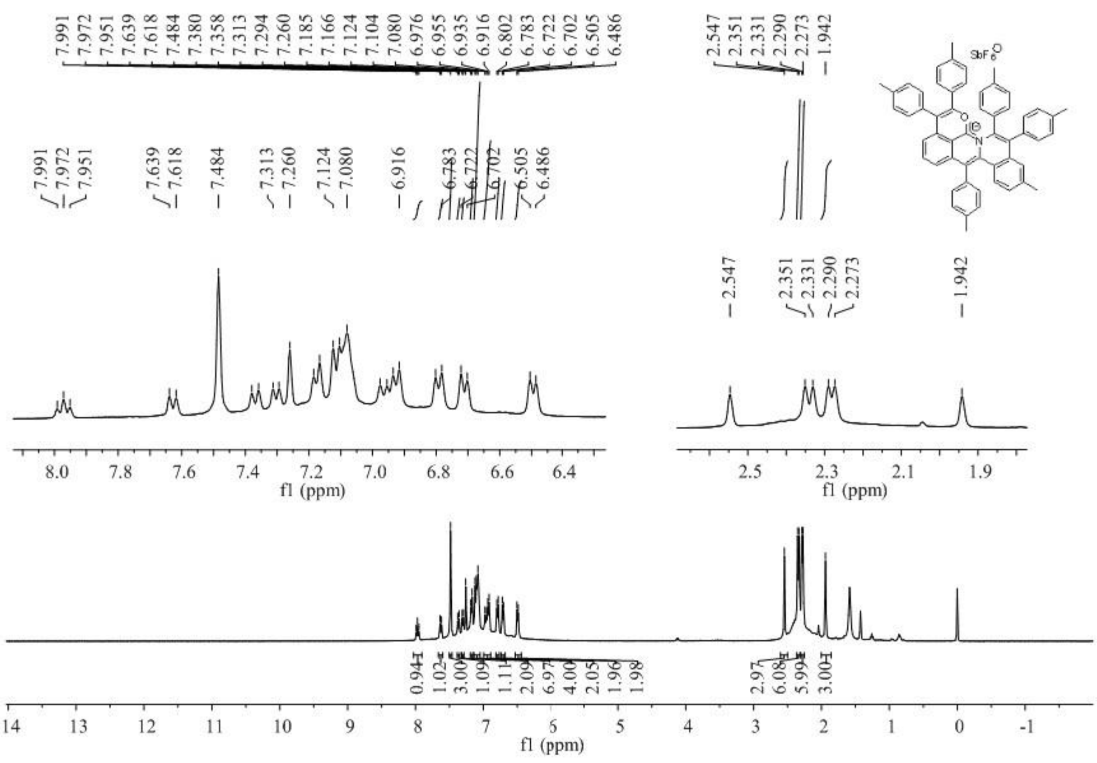

^{13}C NMR spectra of **3ab** ($CDCl_3$)

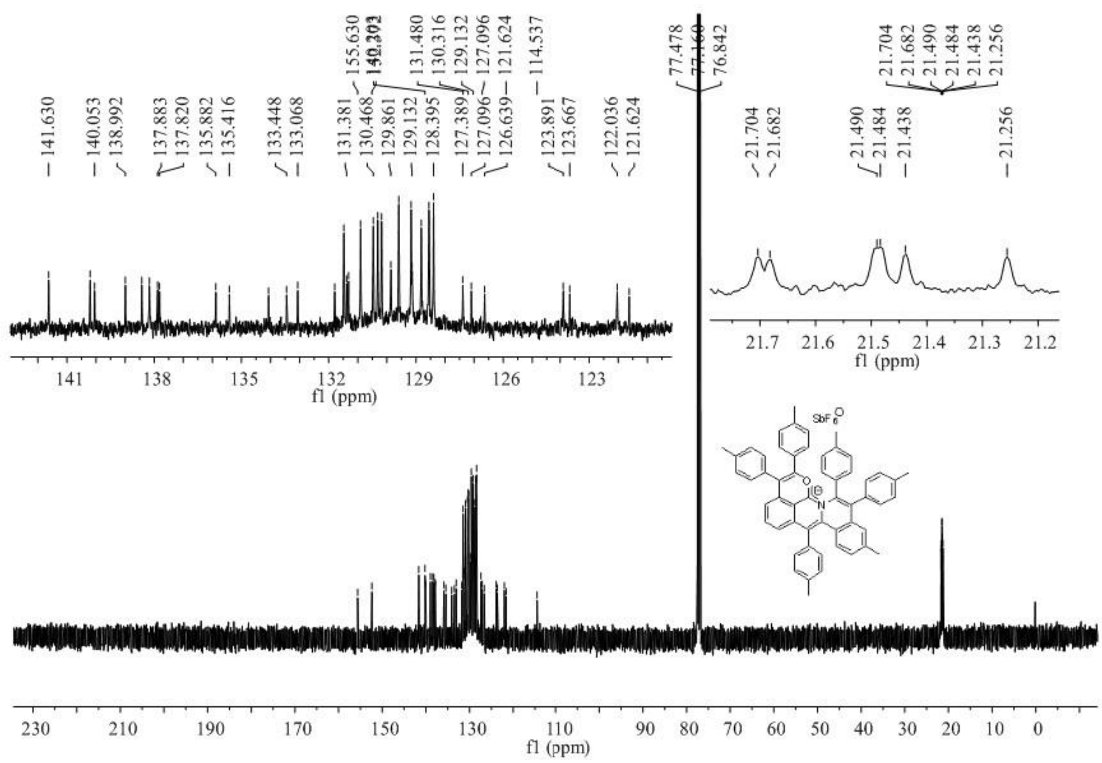

^{1}H NMR spectra of **3ac** ($CDCl_3$)

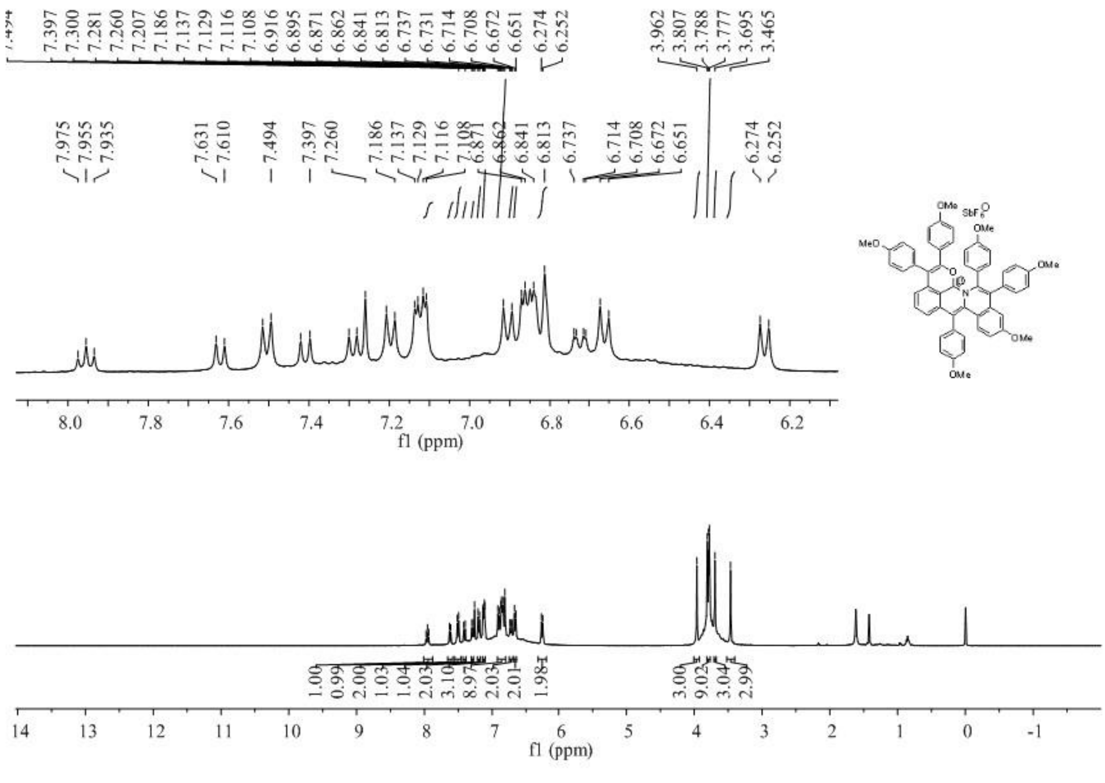

^{13}C NMR spectra of **3ac** ($CDCl_3$)

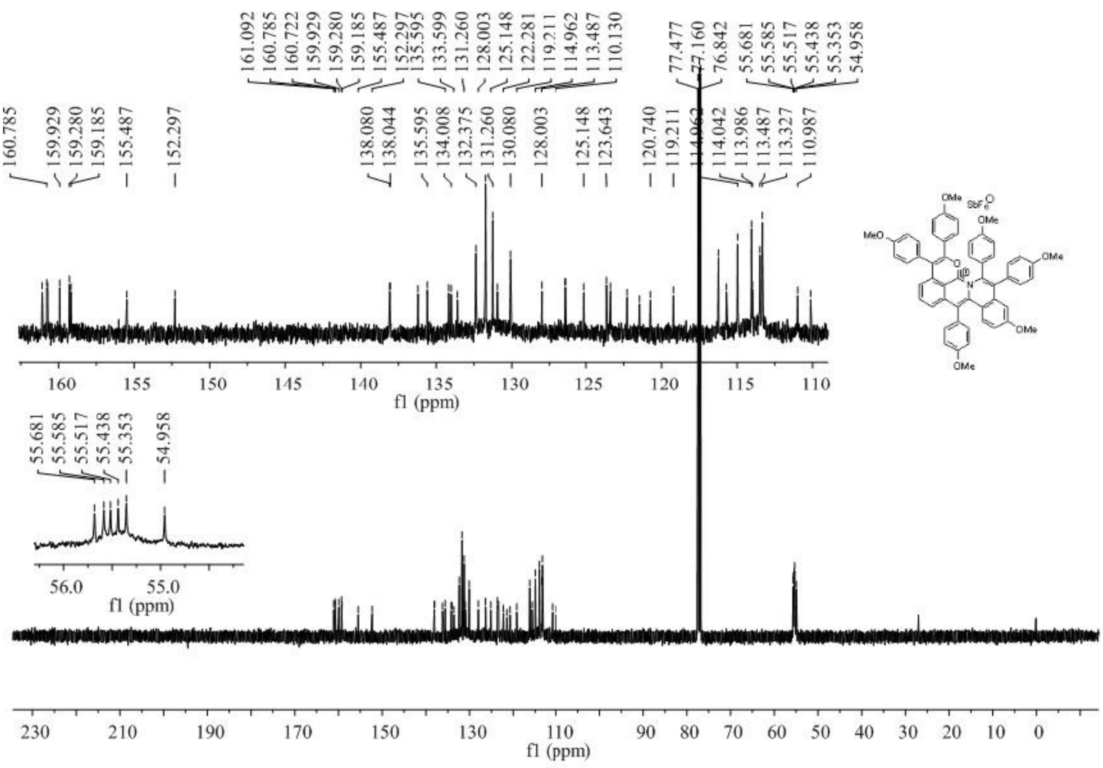

^{1}H NMR spectra of **3ad** ($CDCl_3$)

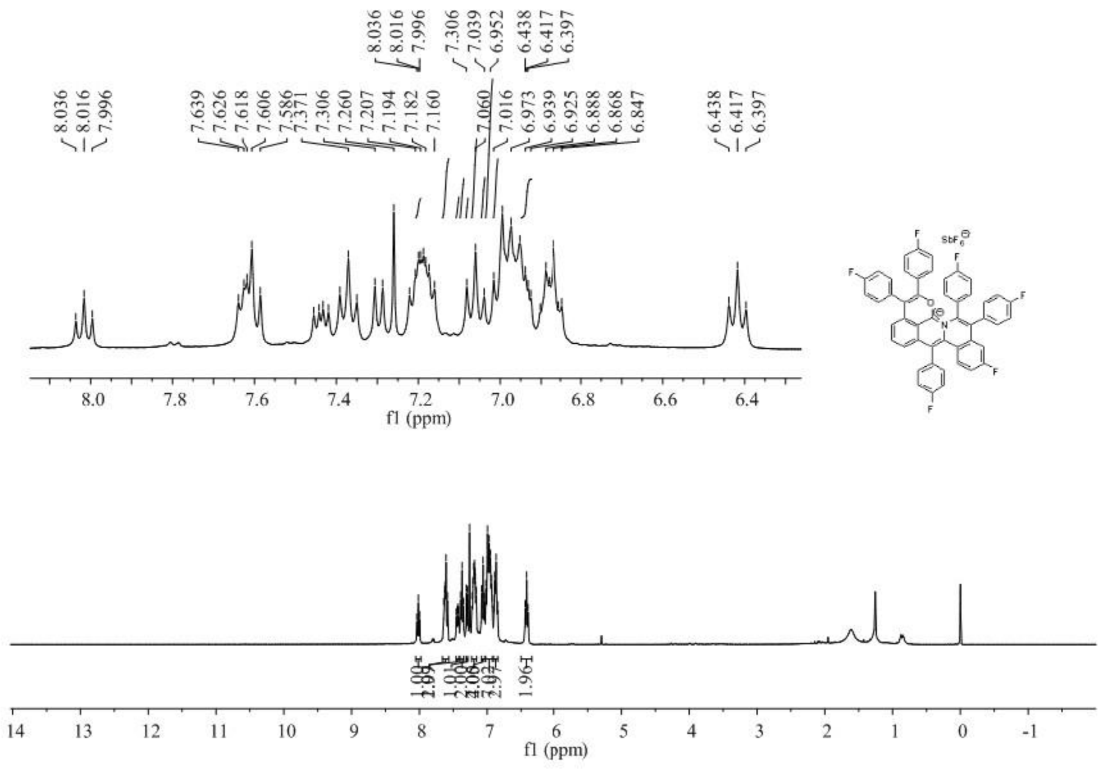

^{13}C NMR spectra of **3ad** ($CDCl_3$)

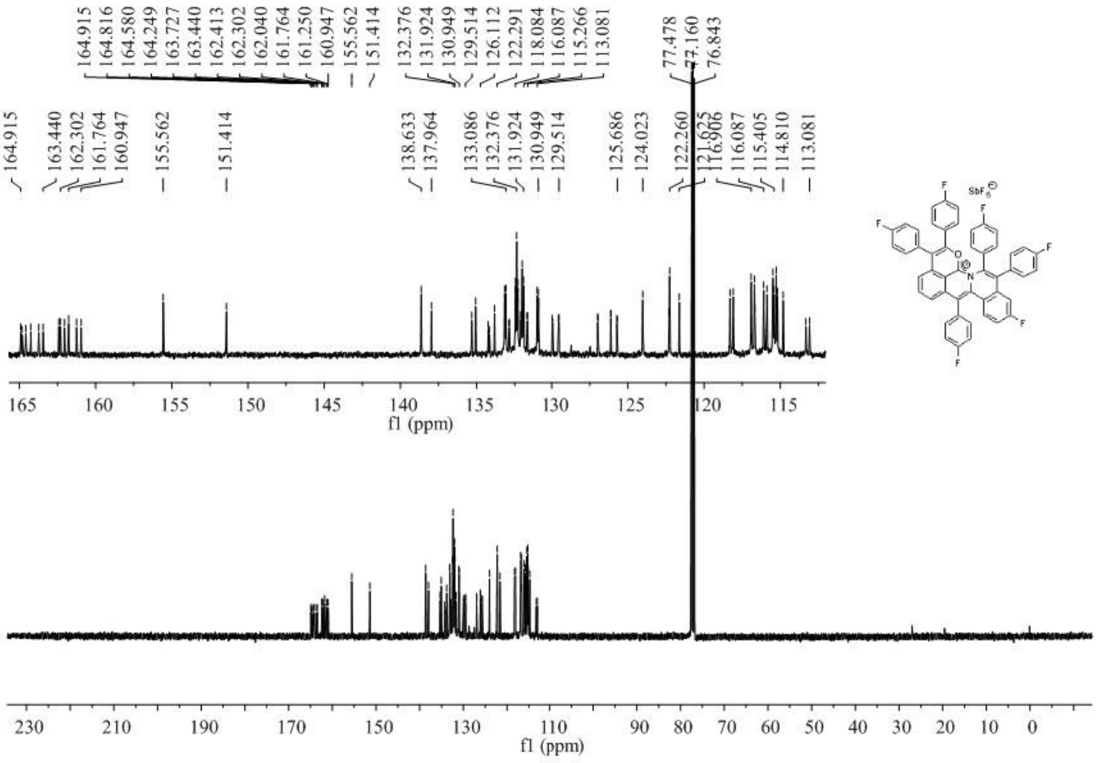

^{19}F NMR spectra of **3ad** ($CDCl_3$)

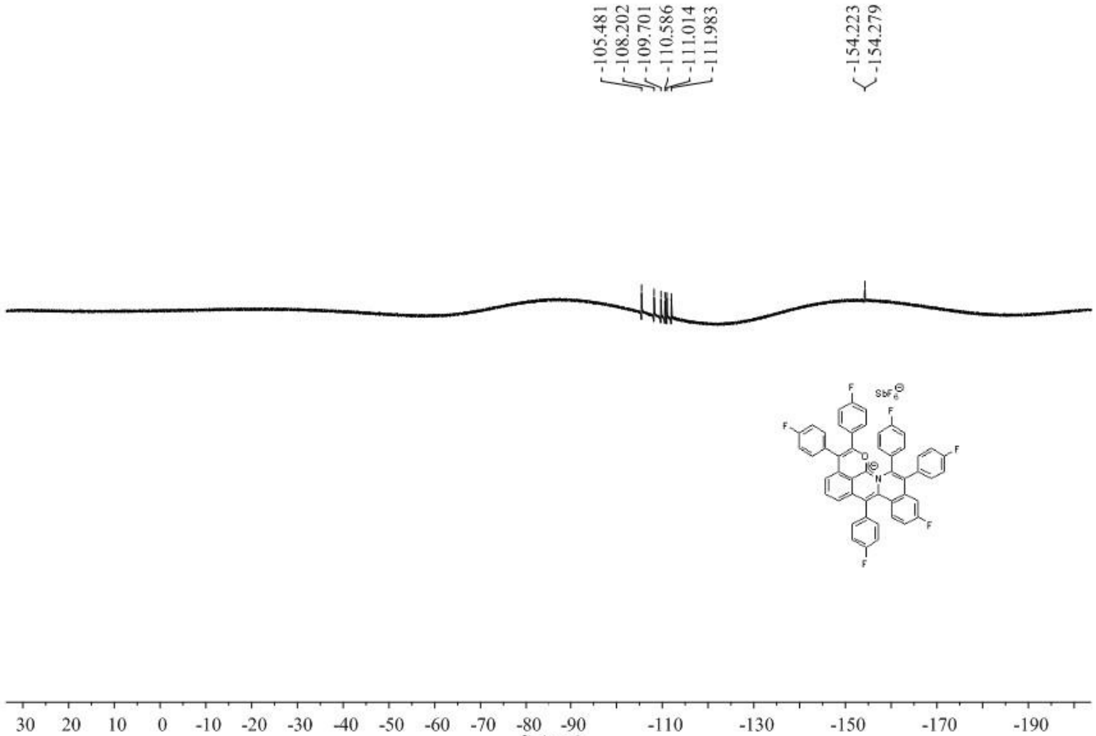

^{1}H NMR spectra of **3ae** ($CDCl_3$)

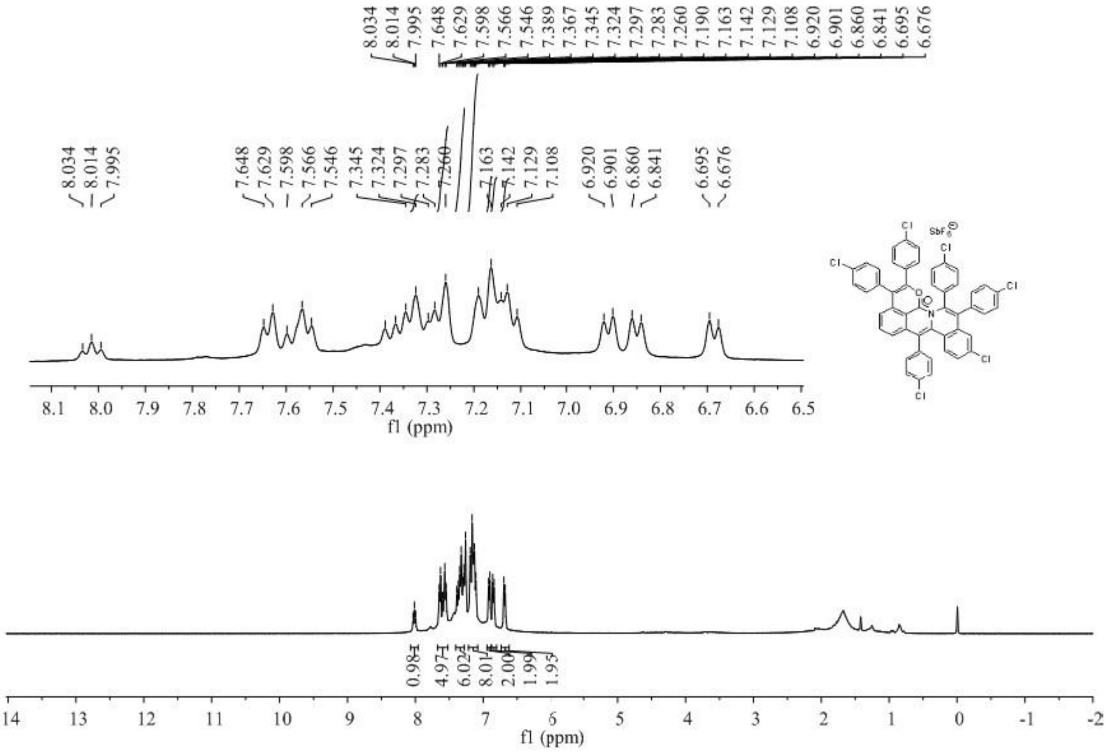

^{13}C NMR spectra of **3ae** ($CDCl_3$)

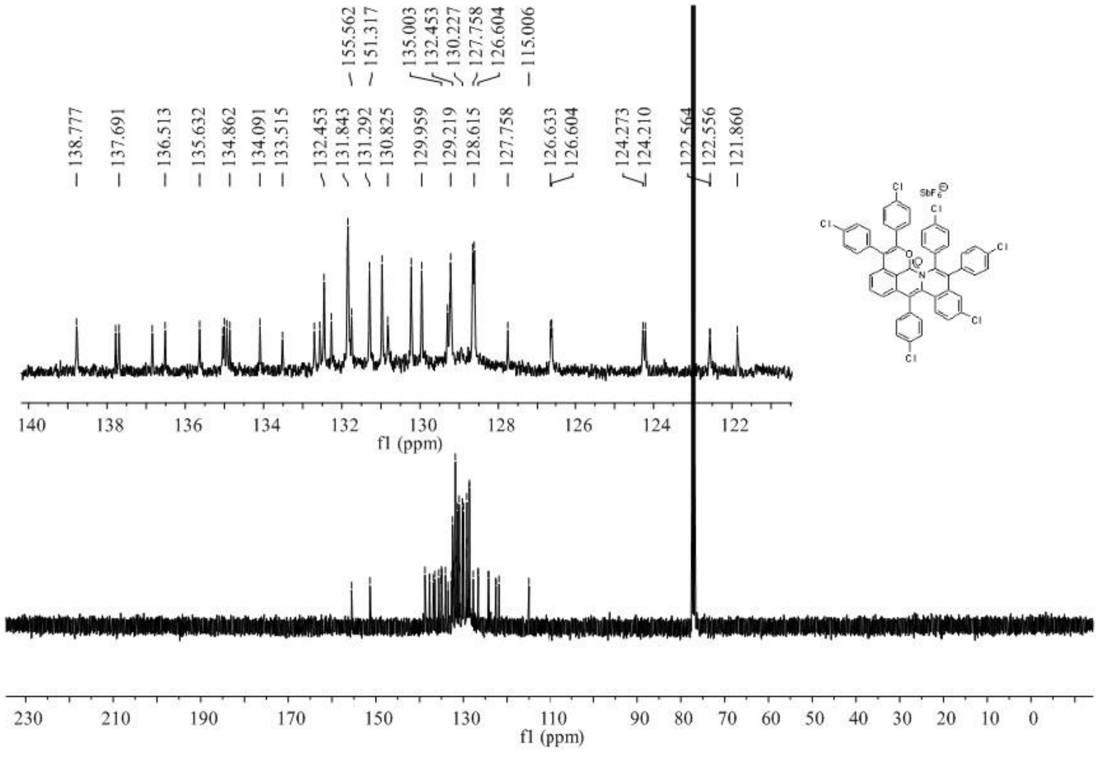

^{1}H NMR spectra of **3af** ($CDCl_3$)

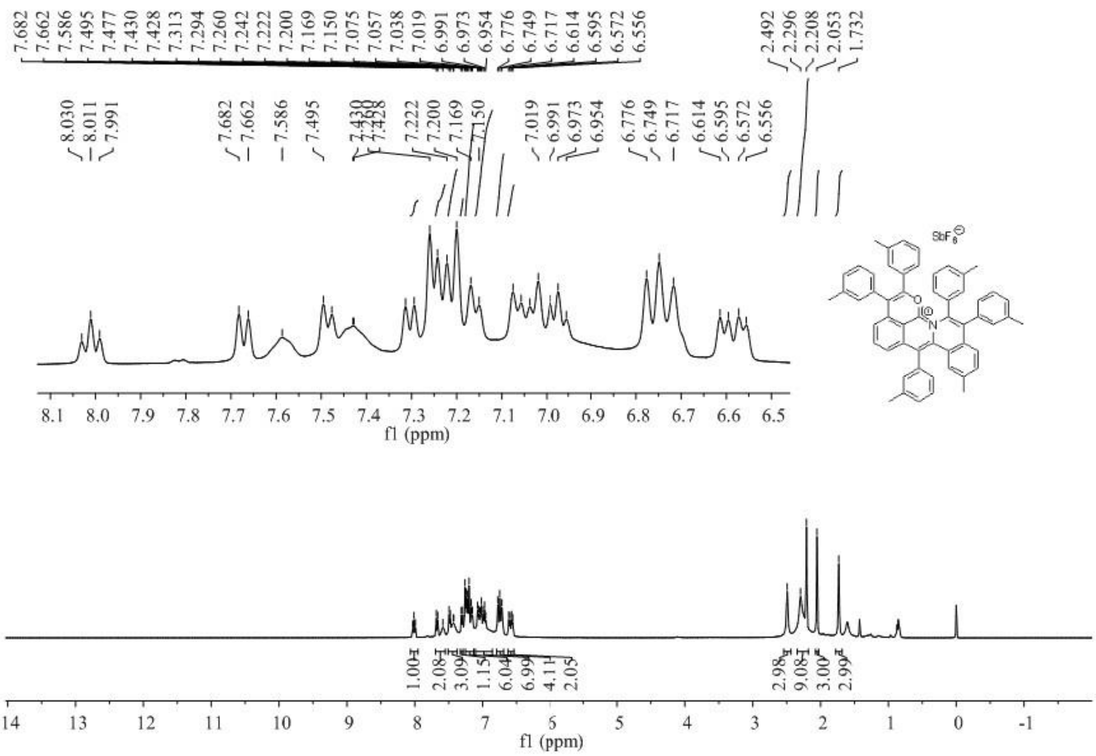

^{13}C NMR spectra of **3af** ($CDCl_3$)

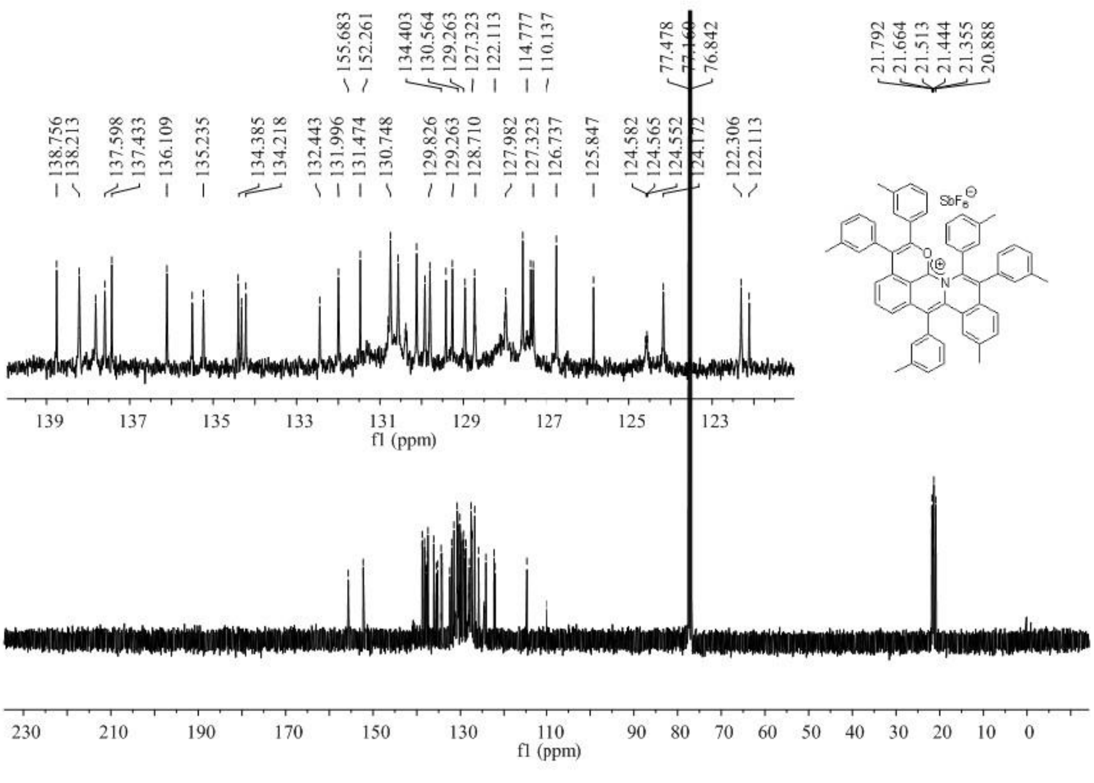

^{1}H NMR spectra of **3mc** ($CDCl_3$)

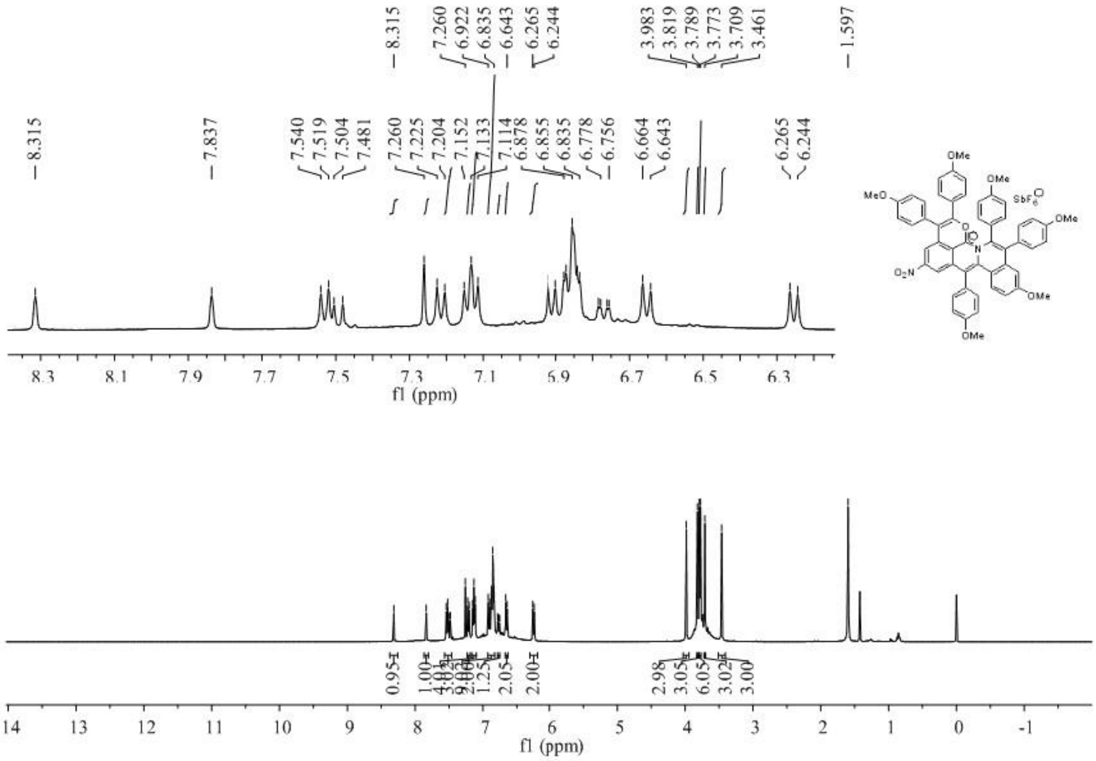

^{13}C NMR spectra of **3mc** ($CDCl_3$)

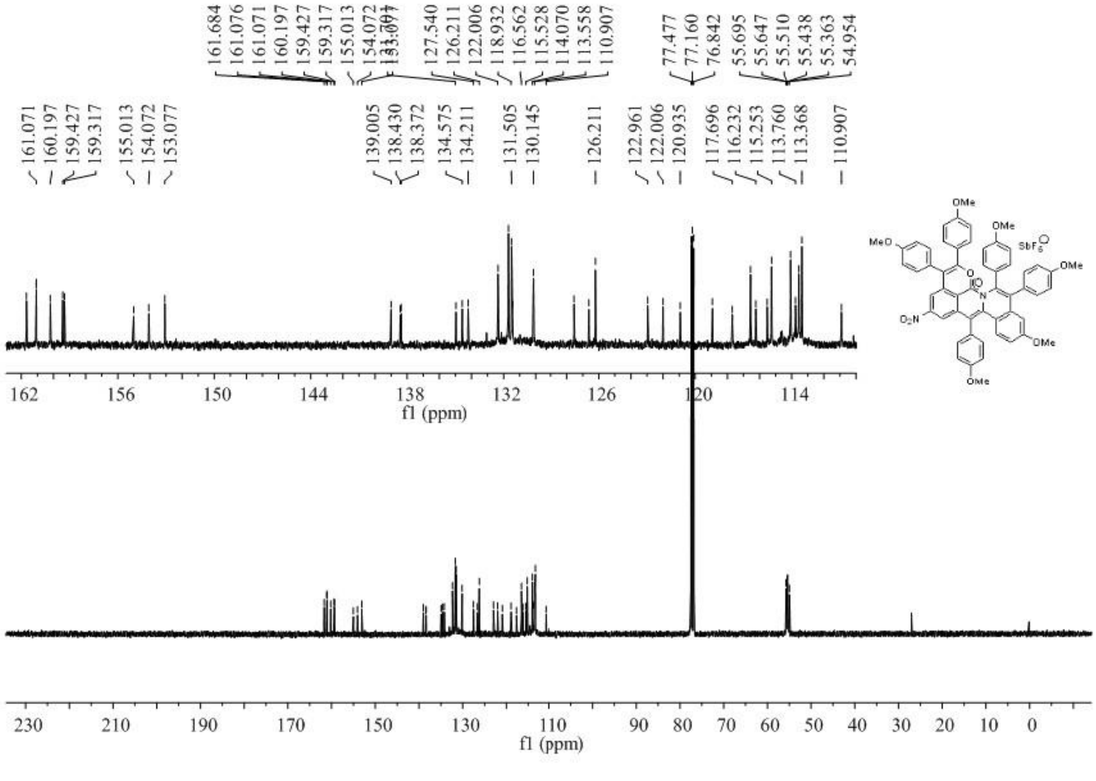

Supplement: Supplementary file 1 [file SC-009-C8SC01963K-s001.pdf]
